# Supplementary material for: Platelet‐derived lipids promote insulin secretion of pancreatic β cells
Source: EMBO Mol Med. 2023 Jul 25;15(9):e16858. doi: 10.15252/emmm.202216858 (PMC10493578; doi:10.15252/emmm.202216858)
Supplement: Supplementary file 1 — Appendix [file EMMM-15-e16858-s002.pdf]

Table of content

Appendix Figure S1 2

Appendix Figure S2. 4

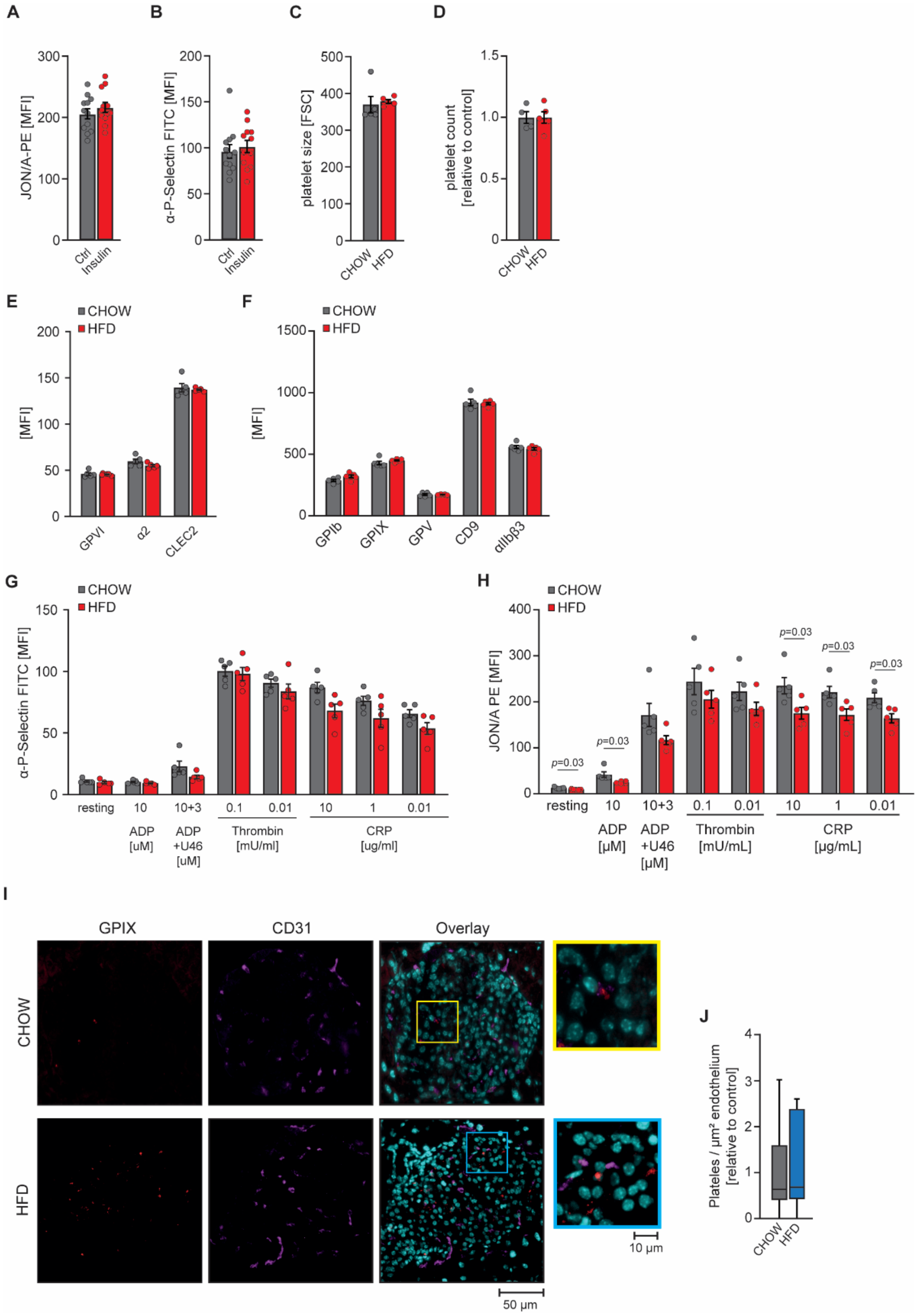

Appendix Figure S1. Insulin and high-fat diet (HFD) feeding do not affect platelet activity.

(**A** and **B**) Integrin activation (**A**) and P-selectin exposure (**B**) of C57BL/6JRj mouse platelets preincubated for 12 min. with 5 ng/ml insulin and 15 mM glucose determined by flow cytometry (n=12). Each n represents the measurement of a sample from a different experiment.

(**C** and **D**) Size (**C**)**,** and count (**D**) of platelets from 16 weeks old male C57BL/6JRj mice fed for the last 12 weeks with regular chow or high fat diet (HFD). (C) CHOW, n=4; HFD, n=5. (D) n=5. Each n represents the measurement of a sample from distinct mice.

(**E** and **F)** Surface expression of indicated integrins, glycoproteins, and receptors on platelets isolated from the mice described above (n=5). Each n represents the measurement of a sample from distinct mice. Glycoprotein Ib (GPIb), Glycoprotein IX (GPIX), Glycoprotein V (GPV), CD9 antigen (CD9), integrin alpha IIb/beta3 (GPIIb/IIIa), Glycoprotein VI (GPVI), Integrin alpha-2 (α2), C-type lectin-like receptor 2 (CLEC2).

(**G** and **H**) P-selectin exposure (**G**) and integrin (**H**) activation assessed by JON/A PE antibody which recognizes activated form of mouse platelet GPIIb/IIIa of platelets isolated from mice described in C and D (n=5). Each n represents the measurement of a sample from distinct mice. U46 - U46619 is a stable thromboxane A2 analog, ADP - Adenosine diphosphate, Thr -Thrombin, CRP - collagen-related peptide

(**I**) Staining of the pancreas from 15 weeks old male mice fed a normal chow diet (CHOW, n=3) or high hat diet (HFD, n=3) for 10 weeks. Antibody against Glycoprotein IX (GPIX) was used to visualize platelets (red), and the endothelium was visualized by CD31 antibody (magenta). Islets were identified by increased nuclear density (nucleus of the cells visualized by DAPI in blue). (**J**) Quantification of the platelets in islets corresponding to Figure I (n=30). Each n represents an image of randomly selected islet.

Mann Whitney test (A-D, J). Kruskal Wallis test followed by Mann Whitney test as post-hoc analysis with Benjamini-Hochberg correction for multiple comparisons (**E-H**). Data are mean ± SEM. Data in boxplots: the center line shows median; box defines first and third quartiles; whiskers indicate 1.5 × interquartile range; outliers are individually plotted (**J**).

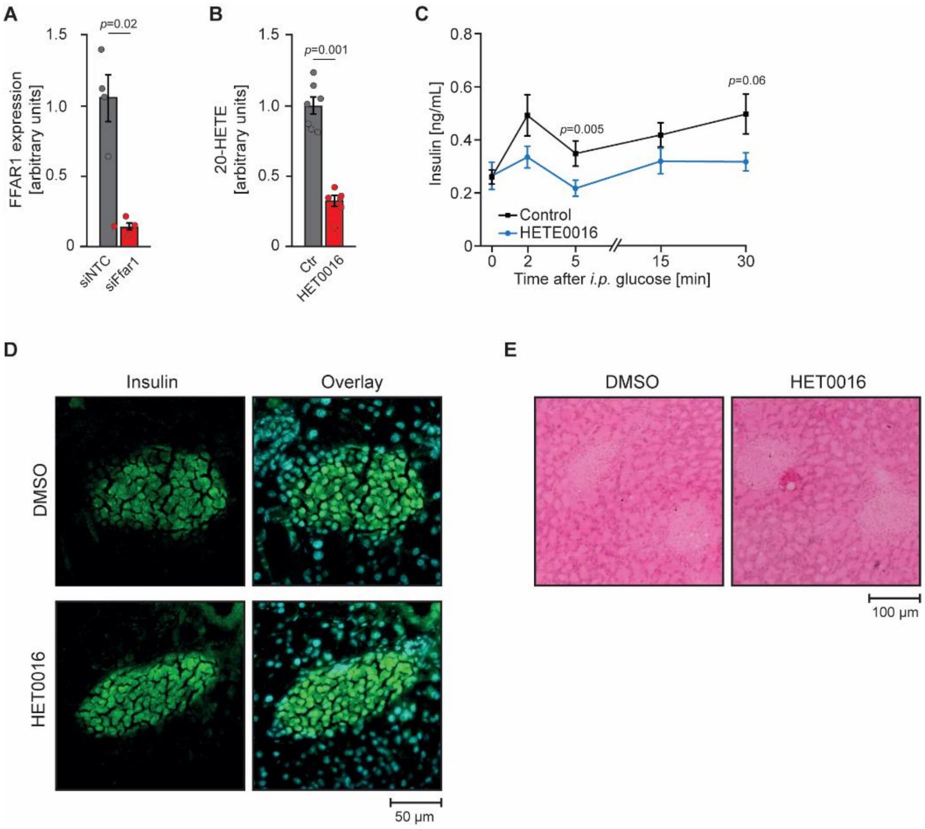

Appendix Figure S2. Inhibition of 20-HETE production reduces glucose-stimulated insulin secretion

(**A**) Relative FFAR1 expression in INS1 cells transfected with FFAR1 siRNA or non-targeting control (NTC) siRNA. (n=4) Each n represents an independent biological replicate.

(**B**) Relative serum levels of 20-HETE from C57BL/6JRj male mice injected i.p. with 10 mg per kg of body weight HET0016 72 h, 48 h, 24 h, and 30 min before the assay with respective control (Ctr). Ctr, n=7; HET0016, n=6. Each n represents the measurement of a sample from distinct mice.

(**C**) Glucose-stimulated insulin secretion on 8 weeks old male mice treated with 10 mg per kg of body weight HET0016 72 h, 48 h, 24 h, and 30 min before the experiment (n=11).

(**D** and **E**) Staining of pancreatic islets with insulin antibody (**D**) and pancreas with H&E staining (**E**) on mice described in figure C.

Mann Whitney test. Data are mean ± SEM.
