# Supplementary material for: Platelet‐derived lipids promote insulin secretion of pancreatic β cells
Source: EMBO Mol Med. 2023 Jul 25;15(9):e16858. doi: 10.15252/emmm.202216858 (PMC10493578; doi:10.15252/emmm.202216858)
Supplement: Supplementary file 2 — Expanded View Figures PDF [file EMMM-15-e16858-s006.pdf]

Expanded View Figures

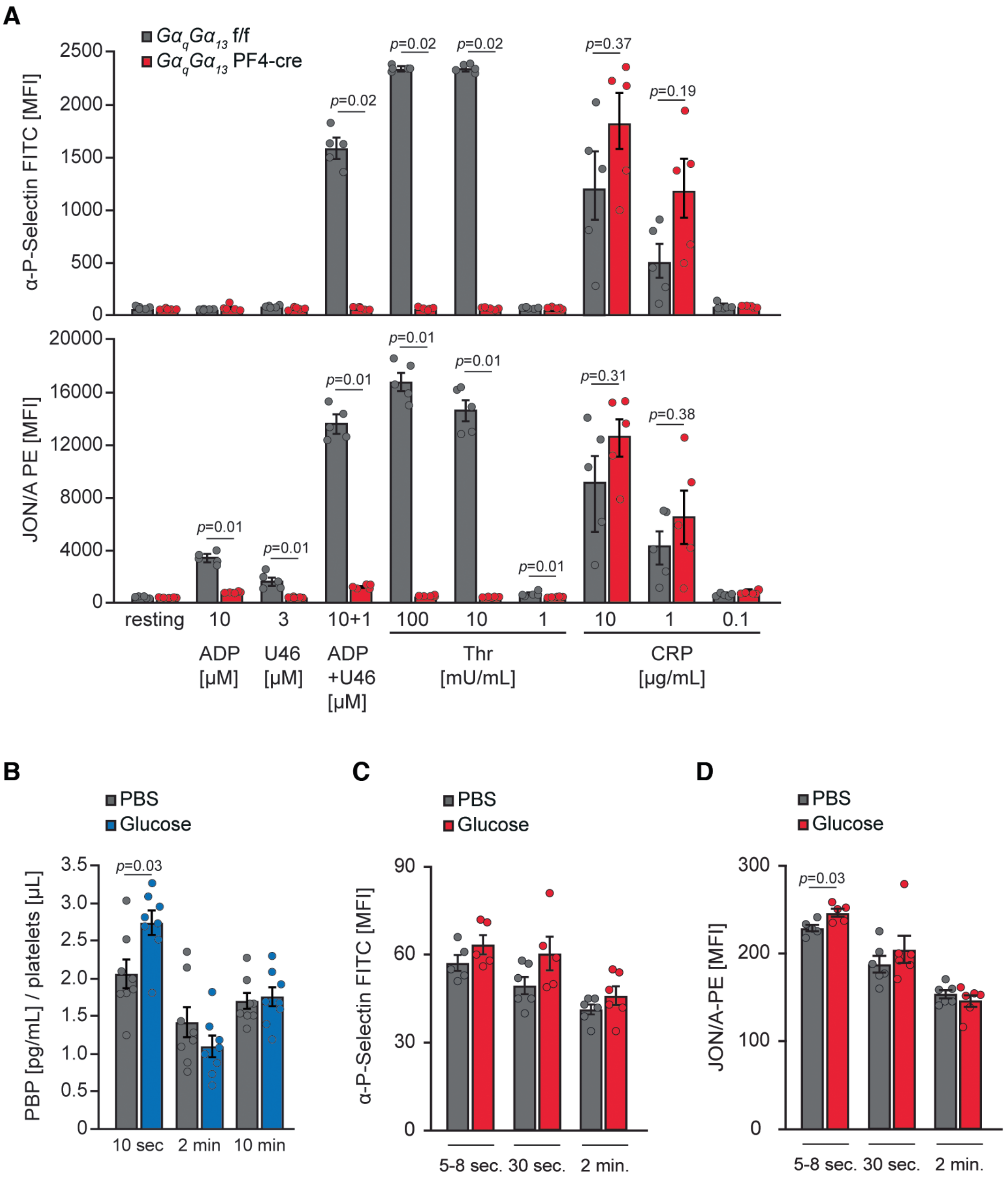

Figure EV1. Glucose promotes platelet activity.

A P-selectin exposure and integrin activation assessed by JONA PE antibody which recognizes an activated form of mouse platelet GPIIb/IIIa, of platelets from male GαqGα13 PF4 Δ/Δ and GαqGα13 f/f mice after stimulation with indicated agonists (determined by flow cytometry). GαqGα13 f/f, n = 5; GαqGα13 PF4 Δ/Δ, n = 5. U46, U46619 is a stable thromboxane A2 analog; ADP, Adenosine diphosphate; Thr, Thrombin; CRP, collagen-related peptide.

B–D Levels of platelet basic protein (PBP) assessed by specific ELISA (B), platelet surface exposure of P-Selectin (C) and integrins (D) (both assessed by flow cytometry using specific antibodies) in the blood of 10-week-old male C57BL/6JRj injected with glucose (2 g per kg of body weight) after indicated time points. PBP, n = 8; P-Selectin and integrins, n = 5.

Data information: Kruskal–Wallis test followed by Mann–Whitney test as *post hoc* analysis with Benjamini–Hochberg correction for multiple comparisons (A). Mann–Whitney test (B–D). Data are mean ± SEM. Each n represents the measurement of a sample from distinct mice.
Source data are available online for this figure.

© 2023 The Authors

Figure EV2. Humoral factors and glucose define reciprocal relation between platelets and pancreatic β cells.

A Representative fluorescence and bright field microscopic images of platelet aggregates formed upon flow adhesion of whole blood from C57BL/6JRj male mice. Before perfusion, mouse blood was incubated for 5 min with supernatants of indicated cells or control supernatant (Ctr). For the generation of supernatants of the different cell types were incubated in the KRB containing 25 mM glucose for 3 min. Control medium have been generated by incubation of the same media on a cell-free culture dish coated with Matrigel.

B Representative bright-field microscopic image obtained during intra-vital imaging of C57BL/6JRj male mouse pancreas with highlighted localized pancreatic islet.

C Representative DAPI and immunostainings of the exocrine and endocrine C57BL/6JRj male mouse pancreas. Mice were i.v. injected with JAQ1 IgG 5 days before and with control IgG, p0p/B $F(ab')_2$, JON/A $F(ab')_2$, or R300 IgG 24 h before organ harvesting.

D Platelet count normalized to islet area. Control, $n = 131$ (six mice); JAQ1 IgG, $n = 71$ (six mice); p0p/B $F(ab')_2$, $n = 113$ (six mice); JON/A $F(ab')_2$, $n = 77$ (six mice), R300 IgG, $n = 27$ (three mice).

Data information: Each *n* represents an image of an islet. Kruskal–Wallis test followed by Mann–Whitney test as *post hoc* analysis with Benjamini–Hochberg correction for multiple comparisons. Data in boxplot: center line shows median; cross indicates mean; box defines first and third quartiles; whiskers indicate 1.5 × interquartile range; outliers are individually plotted.
Source data are available online for this figure.

© 2023 The Authors

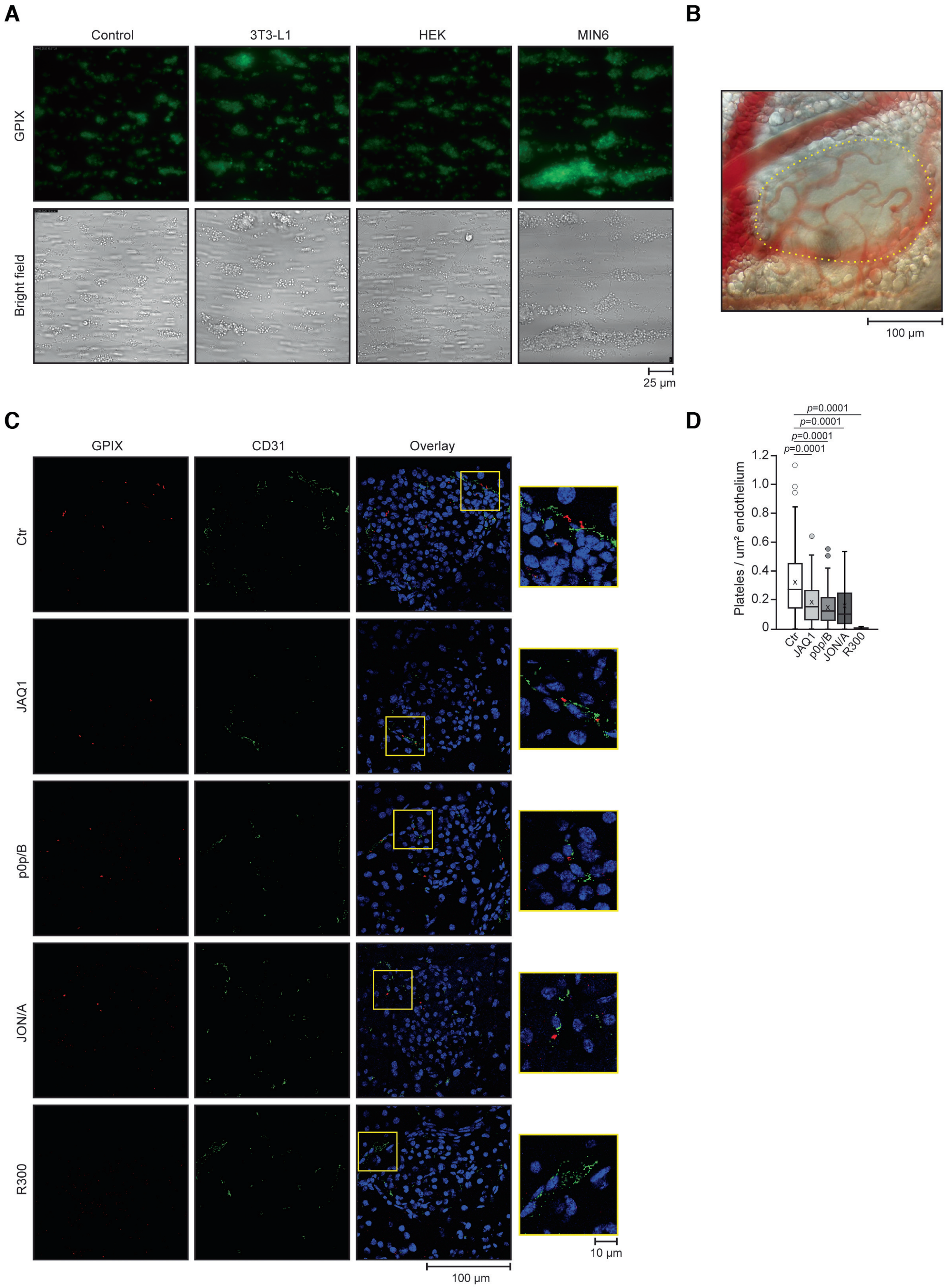

Figure EV2.

 © 2023 The Authors

Figure EV3. Platelets do not affect β cell mass.

A Glucose stimulated (3 g per kg of body weight) release of c-peptide in GαqGα13 f/f and GαqGα13 PF4 Δ/Δ (males, 10 weeks old, n = 4 for GαqGα13 f/f and 4 for GαqGα13 PF4 Δ/Δ).

B Quantification of area under the curve (AUC) from (A).

C Glucose tolerance test (2 g per kg of body weight) in GαqGα13 f/f and GαqGα13 PF4 Δ/Δ (females, 10 weeks old, n = 5 GαqGα13 f/f and 8 for GαqGα13 PF4 Δ/Δ).

D–F The ratio of islet area to whole pancreas area of BMC-Gp1bα$^{-/-,TG}$ (D), BMC-Gp6$^{-/-}$ (E), and GαqGα13 PF4 Δ/Δ (F) male mice relative to the respective wild-type control (ctr). BMC-Gp1bα$^{+/+}$, n = 32 (eight mice, 21 weeks old); BMC-Gp1bα$^{-/-,TG}$, n = 28 (seven mice, 21 weeks old D). BMC-Gp6$^{+/+}$, n = 24 (eight mice, 22 weeks old); BMC-Gp6$^{-/-}$, n = 18 (six mice, 22 weeks old E). GαqGα13 f/f, n = 15 (five mice, 15 weeks old); GαqGα13 PF4 Δ/Δ, n = 12 (four mice, 15 weeks old F). Each n represents the ratio of the islet area to the pancreas area of one tissue section.

G Insulin content of pancreas from GαqGα13 PF4 Δ/Δ and GαqGα13 f/f 9 weeks old male mice normalized to total protein. GαqGα13 f/f, n = 8; GαqGα13 PF4 Δ/Δ, n = 8. Each n represents the measurement of a sample from distinct mice.

H Glucagon serum levels of overnight fasted GαqGα13 PF4 Δ/Δ and GαqGα13 f/f 14 weeks old male mice. GαqGα13 f/f, n = 12; GαqGα13 PF4 Δ/Δ, n = 11. Each n represents the measurement of a sample from distinct mice.

I Insulin release per islet isolated from GαqGα13 PF4 Δ/Δ and GαqGα13 f/f 8–12-week-old male mice upon 2.8 mM (LG) and 16.7 mM glucose (HG). GαqGα13 f/f, n = 16; GαqGα13 PF4 Δ/Δ, n = 38. Each n represents an independent biological replicate.

J, K Body weight and composition of Gp6$^{-/-}$ 24 weeks old (J) and 15-week-old GαqGα13 PF4 Δ/Δ (K) mice with respective control. Gp6$^{+/+}$, n = 4; Gp6$^{-/-}$, n = 4 (J). GαqGα13 f/f, n = 9; GαqGα13 PF4 Δ/Δ, n = 4 (K). Each n represents the measurement of a sample from distinct mice.

Data information: Mann–Whitney test (A–H). Kruskal–Wallis test followed by Mann–Whitney test as *post hoc* analysis with Benjamini–Hochberg correction for multiple comparisons (I–K). Data are mean ± SEM. Data in boxplots: center line shows median; cross indicates mean; box defines first and third quartiles; whiskers indicate 1.5 × interquartile range; outliers are individually plotted (D–F, H, I).
Source data are available online for this figure.

© 2023 The Authors

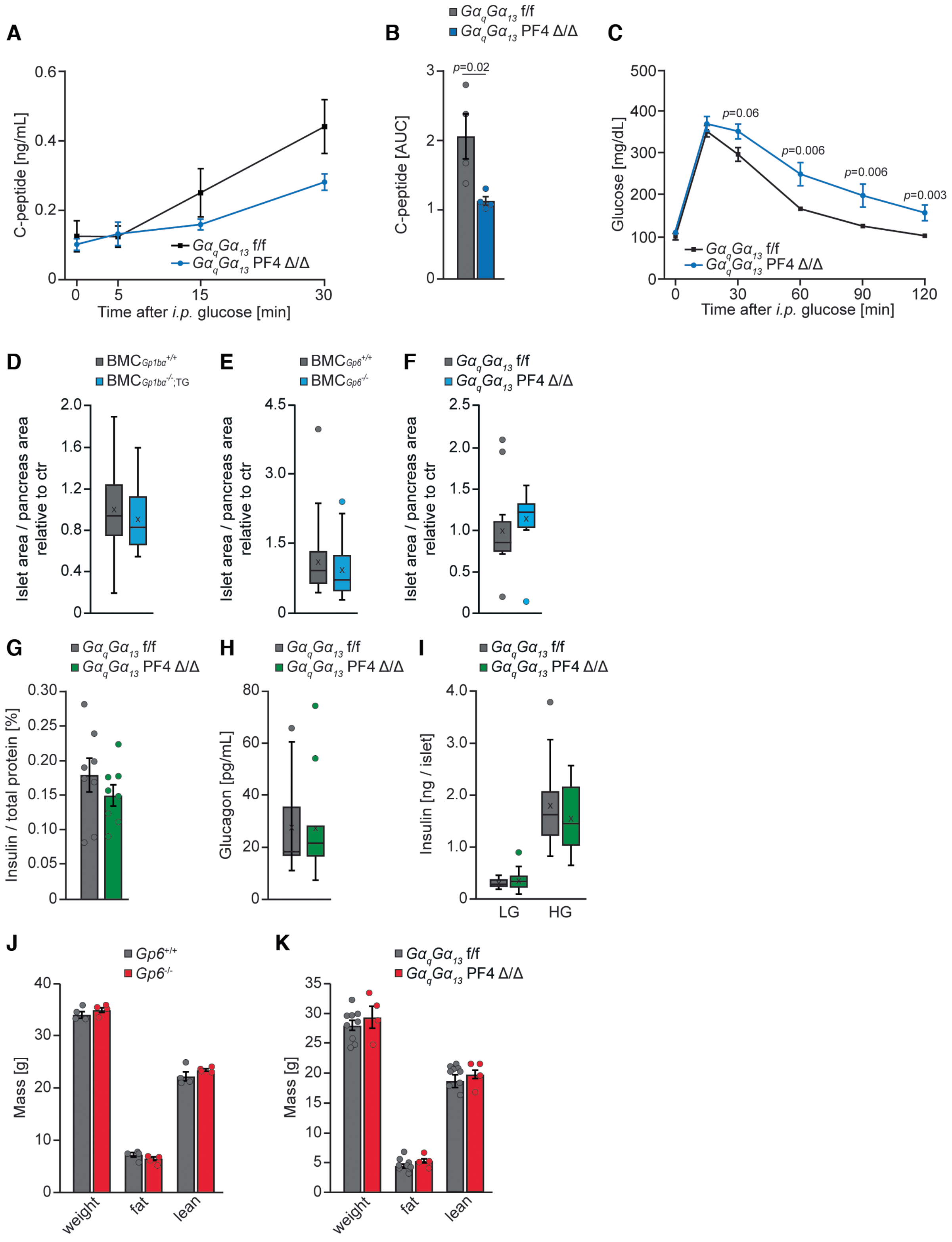

Figure EV3.

 © 2023 The Authors

Figure EV4. A platelet-derived factor stimulates insulin secretion.

A, B Cell surface expression of GPVI (A) and platelet count (B) of 6-week-old, male mice treated with JAQ1-IgG antibody for 5 days (n = 11). Each n represents one injected mouse.

C Cell surface expression of GPVI assessed by flow cytometry using JAQ1-FITC and GPIIb/IIIa verified by the same method using JON/A-FITC antibody in control animals and 6-week-old male mice injected with 4 mg per kg body weight JAQ1-F(ab)$_2$ or JON/A-F(ab)$_2$ 24 h before an experiment (n = 4). Each n represents one injected mouse.

D Platelet count in 6-week-old male mice treated with R300 antibody (2 mg per kg of body weight) 24 h before the experiment. Ctrl IgG, n = 11; R300, n = 10. Each n represents one injected mouse.

E Glucose tolerance test (2 g per kg of body weight) of 6-week-old female mice treated R300 or control IgG antibody (2 mg per kg of body weight) for 24 h before the experiment. Each n represents one injected mouse.

F, G Western blot (WB) analyses using indicated antibodies of extracts isolated from skeletal muscles (quadriceps) (F) and perigonadal adipose tissue (G) of 10-week-old male mice depleted from platelets (using R300 antibody) or corresponding age and sex-matched animals. Each band on the WB corresponds to the tissue isolated from one mouse.

H Insulin secretion from INS1 cells stimulated with a supernatant of activated human platelets (hPS) for indicated time points or control buffer in the presence of 2.8 mM glucose (n = 4). Each n represents an independent biological replicate.

I Insulin secretion of human EndoC-βH1 cells after 15 min of stimulation with supernatant of activated human platelets (hPS) or control buffer upon 2.5 mM glucose (n = 3). Each n represents an independent biological replicate.

Data information: Mann–Whitney test (A–E, I). Kruskal–Wallis test followed by Mann–Whitney test as *post hoc* analysis with Benjamini–Hochberg correction for multiple comparisons (H). Data are mean ± SEM.
Source data are available online for this figure.

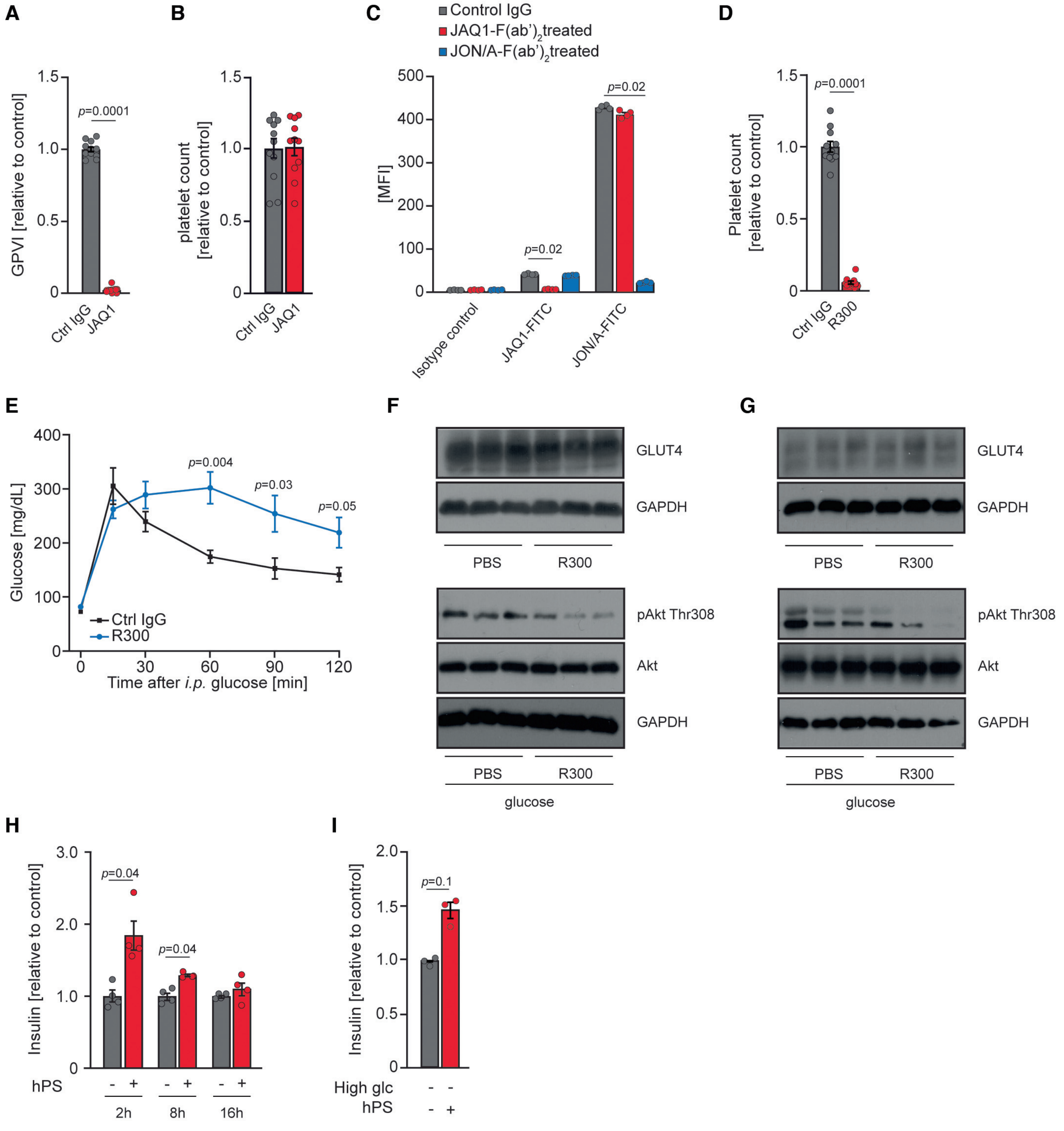
A
B
C
D
Control IgG
JAQ1-F(ab')2treated
JON/A-F(ab')2treated
GPVI [relative to control]
p=0.0001
Ctrl IgG
JAQ1
platelet count [relative to control]
[MFI]
p=0.02
Isotype control
JAQ1-FITC
JON/A-FITC
Platelet count [relative to control]
R300
E
F
G
Glucose [mg/dL]
p=0.004
p=0.03
p=0.05
Ctrl IgG
R300
Time after i.p. glucose [min]
GLUT4
GAPDH
PBS
pAkt Thr308
Akt
glucose
H
I
Insulin [relative to control]
p=0.04
p=0.1
hPS
2h
8h
16h
High glc

Figure EV4.

Figure EV5. The impact of platelets on insulin secretion declines with age.

A Insulin tolerance test of 13-week-old C57BL/6JRj male mice treated with clopidogrel for 7 weeks. Control, $n = 7$; Clopidogrel, $n = 9$.

B Western blot (WB) analysis using indicated antibodies on platelets isolated from 8 weeks control and clopidogrel-treated C57BL/6JRj male mice for 3 weeks. Each line represents a sample from distinct mice.

C Glucose tolerance test (2 g per kg of body weight) of 9-week-old C57BL/6JRj female mice treated with clopidogrel for 3 weeks. Control, $n = 15$; Clopidogrel, $n = 14$.

D Glucose tolerance test (2 g per kg of body weight) of 16-week-old WISTAR rats treated with clopidogrel for 8 weeks. Control, $n = 7$; Clopidogrel, $n = 10$.

E Glucose-stimulated insulin secretion (2 g per kg of body weight) of 17-week-old WISTAR rats treated with clopidogrel for 9 weeks. Control, $n = 9$; Clopidogrel, $n = 9$.

F Insulin tolerance test of 18-week-old WISTAR rats treated with clopidogrel for 10 weeks. Control, $n = 8$; Clopidogrel, $n = 10$.

G P-selectin exposure and integrin activation assessed by flow cytometry using specific antibodies on platelets isolated from young (9 weeks old, $n = 4$) and aged (60 weeks old, $n = 4$) male mice. U46, U46619 is a stable thromboxane A2 analog; ADP, Adenosine diphosphate; Thr, Thrombin; CRP, collagen-related peptide.

H–J Glucose tolerance test (2 g per kg of body weight) on treated with clopidogrel for 3 weeks or control mice. Fourteen weeks old male mice depleted from platelets ($n = 10$) and corresponding control animals ($n = 9$) (H) 13-week-old male mice treated with clopidogrel ($n = 9$) or control solution ($n = 9$) (I) and 14-week-old male GαqGα13 PF4 Δ/Δ mice ($n = 5$) as well as corresponding control GαqGα13 f/f animals ($n = 4$) (J). All animals were subjected to HFD feeding for 8 weeks before the glucose tolerance test.

Data information: Each n represents the measurement of a sample from distinct mice or rats. Mann–Whitney test. Data are mean $\pm$ SEM.
Source data are available online for this figure.

© 2023 The Authors

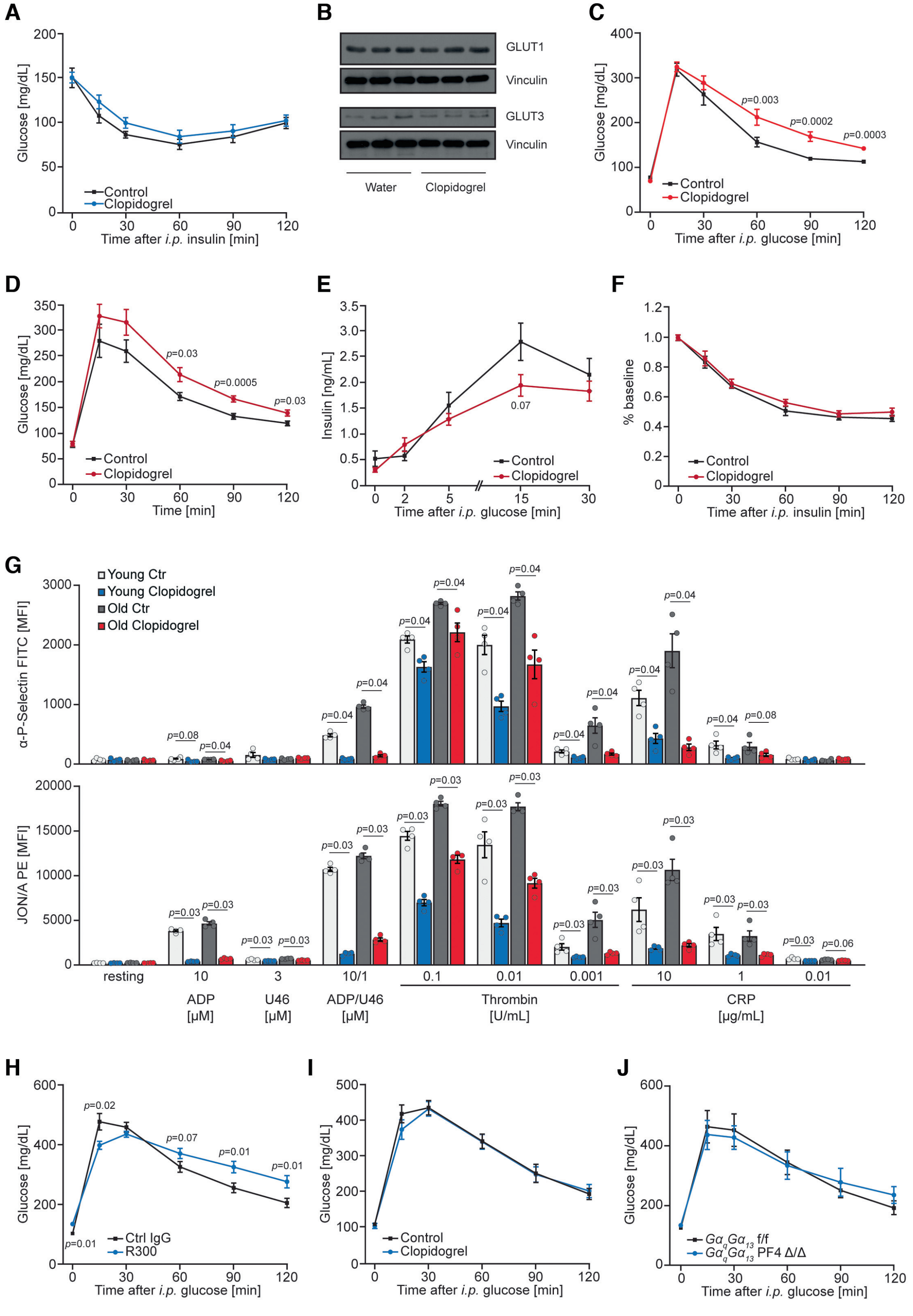

Figure EV5.

 © 2023 The Authors
