# Supplementary material for: Platelet‐derived lipids promote insulin secretion of pancreatic β cells
Source: EMBO Mol Med. 2023 Jul 25;15(9):e16858. doi: 10.15252/emmm.202216858 (PMC10493578; doi:10.15252/emmm.202216858)
Supplement: Supplementary file 3 — Table EV1 [file EMMM-15-e16858-s008.docx]

**Table EV1. Retention time and m/z values of identified lipids by LC/MS lipidomic analysis.**

| **Lipid** | | **Retention time [min]** | **m/z [Da]** |
| --- | --- | --- | --- |
| HETE | Hydroxyeicosatetraenoic acid | 6.8 - 7.8 | 319.228 |
| LPEA-(24:0) | Lysophosphatidylethanolamine | 8.1 - 9.7 | 566.418 |
| FA-(20:5) | Fatty Acid | 6.2 - 8.1 | 301.217 |
| PA-(34:1) | Phosphatidate | 10.6 - 13.5 | 673.481 |
| AC-(16:0) | Acyl Carnitine | 6.5 - 7.2 | 400.342 |
| PEA-(38:6) | Phosphatidylethanolamine | 8.8 - 9.9 | 764.522 |
| LPEA-(18:0) | Lysophosphatidylethanolamine | 7.5 - 8.5 | 482.324 |
| LPEA-(16:0) | Lysophosphatidylethanolamine | 7.0 - 8.0 | 454.293 |
| LPEA-(20:3) | Lysophosphatidylethanolamine | 7.0 - 8.3 | 504.308 |
| BMP-(34:2) | Bis(monoacylglycero)phosphate | 11.8 - 14.2 | 745.503 |
| BMP-(34:3) | Bis(monoacylglycero)phosphate | 11.1 - 13.7 | 743.487 |
| PlasEA-(38:4) | Plasmalogenethanolamine | 9.3 - 11.1 | 752.559 |
| LPI-(16:0) | Lysophosphatidylinositole | 7.9 - 9.7 | 571.289 |
| Sphingosin-P | Sphingosin-1-phosphate | 6.7 - 7.7 | 378.241 |
| lysoPAF-(18:1) | Lyso-platelet-activating-factor | 7.3 - 8.5 | 508.376 |
| LPC-(20:1) | Lysophosphatidylcholine | 7.4 - 8.7 | 550.387 |
| PC-(36:0) | Phosphatidylcholine | 11.5 - 13.4 | 790.632 |
| Cer-(24:1) | Cerebroside | 10.3 - 11.3 | 810.682 |
| LPI-(18:0) | Lysophosphatidylinositole | 8.7 - 10.2 | 599.320 |
| LPEA-(22:6) | Lysophosphatidylethanolamine | 6.7 - 7.7 | 526.293 |
| Cer-(22:6) | Cerebroside | 9.3 - 10.2 | 772.572 |
| PS-(40:3) | Phosphatidylserines | 8.4 - 9.7 | 842.591 |
