# Supplementary material for: Platelet‐derived lipids promote insulin secretion of pancreatic β cells
Source: EMBO Mol Med. 2023 Jul 25;15(9):e16858. doi: 10.15252/emmm.202216858 (PMC10493578; doi:10.15252/emmm.202216858)
Supplement: Supplementary file 4 — PDF+ [file EMMM-15-e16858-s010.pdf]

Article

SOURCE DATA TRANSPARENT PROCESS OPEN ACCESS

EMBO Molecular Medicine

Platelet-derived lipids promote insulin secretion of pancreatic β cells

Till Karwen[1,†], Katarzyna Kolczynska-Matysiak[2,†], Carina Gross[3], Mona C Löffler[1], Mike Friedrich[1], Angel Loza-Valdes[2], Werner Schmitz[4], Magdalena Wit[2], Filip Dziaczkowski[2], Andrei Belykh[2], Jonathan Trujillo-Viera[1], Rabih El-Merahbi[1], Carsten Deppermann[1,5], Sameena Nawaz[6], Benoit Hastoy[6], Agnieszka Demczuk[2], Manuela Erk[1], Mariusz R Wieckowski[2], Patrik Rorsman[6,7,8], Katrin G Heinze[1], David Stegner[1,3,*], Bernhard Nieswandt[1,3,**] & Grzegorz Sumara[1,2,***]

Abstract

Hyperreactive platelets are commonly observed in diabetic patients indicating a potential link between glucose homeostasis and platelet reactivity. This raises the possibility that platelets may play a role in the regulation of metabolism. Pancreatic β cells are the central regulators of systemic glucose homeostasis. Here, we show that factor(s) derived from β cells stimulate platelet activity and platelets selectively localize to the vascular endothelium of pancreatic islets. Both depletion of platelets and ablation of major platelet adhesion or activation pathways consistently resulted in impaired glucose tolerance and decreased circulating insulin levels. Furthermore, we found platelet-derived lipid classes to promote insulin secretion and identified 20-Hydroxyeicosatetraenoic acid (20-HETE) as the main factor promoting β cells function. Finally, we demonstrate that the levels of platelet-derived 20-HETE decline with age and that this parallels with reduced impact of platelets on β cell function. Our findings identify an unexpected function of platelets in the regulation of insulin secretion and glucose metabolism, which promotes metabolic fitness in young individuals.

Keywords 20-HETE; diabetes; insulin secretion; platelet; β cell
Subject Categories Haematology; Metabolism; Vascular Biology & Angiogenesis
DOI 10.15252/emmm.202216858 | Received 8 September 2022 | Revised 4 July 2023 | Accepted 7 July 2023 | Published online 25 July 2023
EMBO Mol Med (2023) 15: e16858

Introduction

Platelets are small anucleate blood cells responsible for the maintenance of vascular integrity (Burkard *et al*, 2020). At the site of injury, platelets recognize and adhere to exposed extracellular matrix constituents through a multi-step process involving the sequential action of different membrane receptors, such as glycoproteins (GP)Ibα, GPVI, and GPIIb/IIIa (integrin αIIbβ3). This results in the sealing of the injured vessel wall and triggers the degranulation of platelets (Mancuso & Santagostino, 2017). Many of the substances released act on G-protein-coupled receptors (GPCRs) signaling through Gαq and/or Gα13 proteins (Moers *et al*, 2004), which further promote platelet activation, degranulation, and the functional upregulation of GPIIb/IIIa to bind fibrinogen and other multimeric ligands resulting in platelet aggregation and further degranulation (Mancuso & Santagostino, 2017). Of note, platelet activation is accompanied by the conversion of lipid precursors in signaling lipids that can be released from the cells (Duvernay *et al*, 2015; Peng *et al*, 2018).

Energy homeostasis is ensured by a complex interplay between multiple organs mediated by nutrients and hormones (Ashcroft & Rorsman, 2012). Pancreatic islets secrete two major hormones that regulate circulating levels of glucose and other nutrients. Insulin is secreted by pancreatic β cells in response to the elevation of glucose levels, glucagon is secreted by α cells during the time of nutrient shortage. Multiple endo-, para-, and autocrine factors modulate β cell function (Ashcroft & Rorsman, 2012). Among them, different

1 Rudolf Virchow Center for Integrative and Translational Bioimaging, Julius-Maximilians University of Würzburg, Würzburg, Germany
2 Nencki Institute of Experimental Biology, Polish Academy of Sciences, Warszawa, Poland
3 Institute of Experimental Biomedicine I, University Hospital Würzburg, Würzburg, Germany
4 Theodor Boveri Institute, Biocenter, University of Würzburg, Würzburg, Germany
5 Center for Thrombosis and Hemostasis, University Medical Center of the Johannes Gutenberg-University, Mainz, Germany
6 Radcliffe Department of Medicine, Oxford Centre for Diabetes, Endocrinology and Metabolism, Churchill Hospital, Oxford, UK
7 Department of Physiology, Institute of Neuroscience and Physiology, University of Göteborg, Göteborg, Sweden
8 Oxford National Institute for Health Research, Biomedical Research Centre, Churchill Hospital, Oxford, UK
*Corresponding author. Tel: +49 931 3180419; E-mail: stegner@uni-wuerzburg.de
**Corresponding author. Tel: +49 931 31 80405; E-mail: bernhard.nieswandt@uni-wuerzburg.de
***Corresponding author. Tel: +48 22 5892 190; E-mail: g.sumara@nencki.edu.pl
†These authors contributed equally to this work

© 2023 The Authors. Published under the terms of the CC BY 4.0 license

lipid classes execute an insulinotropic effect by signaling through receptors such as G-protein-coupled receptor (GPR) 40, 55, and 120 (Kristinsson *et al*, 2013; McKillop *et al*, 2013; Moran *et al*, 2014; Tunaru *et al*, 2018). Loss of β cell function results in hyperglycemia, a hallmark of diabetes. In type 1 diabetes, autoimmune destruction of β cells leads to absolute insulin deficiency, whereas in type 2 diabetes, obesity and peripheral insulin resistance result in relative insulin deficiency that may culminate in glucolipotoxicity causing β cell death (Hall *et al*, 2019). Both type 1 and type 2 diabetes are associated with an increased prevalence of vascular diseases (Laakso & Lehto, 1998), that besides other factors, is driven by increased platelet reactivity (Mylotte *et al*, 2012; Mahmoodian *et al*, 2019). This suggests that platelets might become activated in response to high glucose and raises a possibility that these cells might be implicated in the systemic response to elevated glucose levels.

We assessed the contribution of platelets to homeostatic function using genetic and pharmacological approaches in combination with intravital imaging and computational techniques. We reveal that platelets specifically interact with the microvasculature of pancreatic islets and that genetic or pharmacological interference with major platelet adhesion/activation pathways consistently results in decreased glucose-induced insulin release leading to glucose intolerance. Using co-culture experiments we show that platelet-derived 20-HETE directly increases insulin secretion in mouse, rat, and human β cells. Notably, the levels of platelet-derived 20-HETE decrease during aging. Therefore, platelet-mediated insulin secretion decline with age. Taken together, our results demonstrate that platelets directly stimulate pancreatic β cell function and platelet-derived lipids contribute to metabolic fitness in young individuals.

Results

Humoral factors and glucose define reciprocal relation between platelets and pancreatic β cells

As diabetic patients are prone to platelet hyperreactivity (Mylotte *et al*, 2012; Mahmoodian *et al*, 2019), we tested whether high glucose levels *per se* increase platelet reactivity. Therefore, we studied thrombus formation on collagen in a whole blood perfusion system (Stritt *et al*, 2017) under low, medium, or high glucose concentrations (2.8, 5, and 25 mM, respectively). While 2.8 mM glucose is commonly considered as low glucose levels in multiple cell culture systems (Burns *et al*, 2015), the physiological concentration of fasting glucose in blood oscillates around 5 mM in healthy subjects and the highest concentration corresponding to that occurring in untreated diabetes. The adhesion of mouse platelets to collagen was increased in response to 25 mM glucose compared to the cells incubated both with 2.8 and 5 mM as indicated by increased surface coverage and the integrated density of the platelets (Fig 1A–C). Importantly, incubation of mouse or human platelets with 5 mM glucose did not alter their adhesion to the collagen-coated surface compared to the cells incubated with 2.8 mM (Fig 1A–C). Platelets deficient for Gαq and Gα13lack activation by major soluble agonists such as thrombin, ADP, or thromboxane A2 (Wettschureck *et al*, 2001; Moers *et al*, 2003, 2004). We confirmed that the deletion of Gαq and Gα13 in platelets results in markedly reduced exposure of P-selectin and Integrin αβ3 in response to these substances (Fig EV1A). Consistently, platelets deficient for Gαq and Gα13 did not aggregate on the collagen-coated surface regardless of glucose levels (Fig 1A–C). Similarly, human platelets incubated with high glucose presented increased reactivity towards the collagen-coated surface, while incubation with 5 mM glucose did not alter their adhesion compared to the cells incubated with 2.8 mM (Fig 1D–F). To verify, if elevated glucose concentration promotes platelet activation we injected mice with glucose (2 g per kg of body weight). As indicated by the levels of Platelet basic protein (PBP), as well as exposure of P-selectin and Integrins on the surface of the platelets glucose transiently prime the activation of platelets (Fig EV1B–D). Altogether, these results demonstrate that high glucose levels promote platelet activation *in vitro* and *in vivo*.

In response to glucose, pancreatic β cells secrete insulin to promote peripheral glucose uptake. To test if β cell-derived factors contribute to the activation of platelets, we again utilized the whole blood perfusion system (Stritt *et al*, 2017). Pre-incubation of blood with supernatants of a pancreatic β cell line (Min6) promoted platelet adhesion to collagen, while pre-incubation of blood with supernatants derived from adipocytes (3T3L1 cells) or Hek293 cells had no such effect (Figs 2A and B, and EV2A), suggesting that only β cells secrete the active factor(s) that promotes platelet activity. To corroborate these results, we preincubated platelets with supernatants derived from Min6 cells and other cell lines that had been maintained in the presence of low or high glucose before. Platelets exposed to supernatants derived from glucose-stimulated Min6 cells presented higher reactivity characterized by increased integrin activation and degranulation-dependent P-selectin exposure, the latter a marker for activation-dependent degranulation (Fig 2C and D). Insulin, which is a major factor released by pancreatic β cells, was previously proposed to affect platelet activation, albeit conflicting results have been published (Yngen *et al*, 2001; Hu *et al*, 2002; Ferreira *et al*, 2004). In our experiment insulin did not affect integrin and P-selectin exposure by platelets (Appendix Fig S1A and B). Together, these results indicated that both glucose and β cell-derived factors promote platelet activation and raised the possibility that platelets might directly interact with pancreatic β cells or become transiently activated in the vasculature of the pancreatic islets. We addressed this by performing *in vivo* 3D confocal live imaging to detect the localization of platelets to the endothelium of pancreatic islets. In the exposed pancreas from mice, we identified pancreatic islets by morphology and vascular density (Fig EV2B). Image processing incorporating the distance of platelets to endothelium and platelet size enabled us to distinguish adherent platelets from motile ones. The number of platelets located at the endothelium of the endocrine pancreas was nearly double at the vasculature relative to the exocrine compartment (Fig 2E and F). We confirmed these data using immunohistochemistry of fixed pancreas sections. Platelets were detected more frequently at the endothelium of the endocrine than the exocrine pancreas (Fig 2G and H). Depletion of the activating platelet collagen/fibrin receptor GPVI from the platelet surface by the JAQ1 antibody (Nieswandt *et al*, 2001), or blocking of the main ligand-binding site of GPIbα by Fab fragments of the p0p/B antibody (Massberg *et al*, 2003), or GPIIb/IIIa by $F(ab)_2$ fragments of JON/A (Bergmeier *et al*, 2002; Stegner

 © 2023 The Authors

et al, 2022) markedly reduced interaction of platelets with the endothelium of the endocrine pancreas. Removal of platelets from the circulation by R300 antibody (Emfret Analytics) (Bergmeier et al, 2000) resulted in complete loss of the platelets from the endothelium of the pancreatic islets, proving specificity of stainings for platelets (Fig EV2C and D).

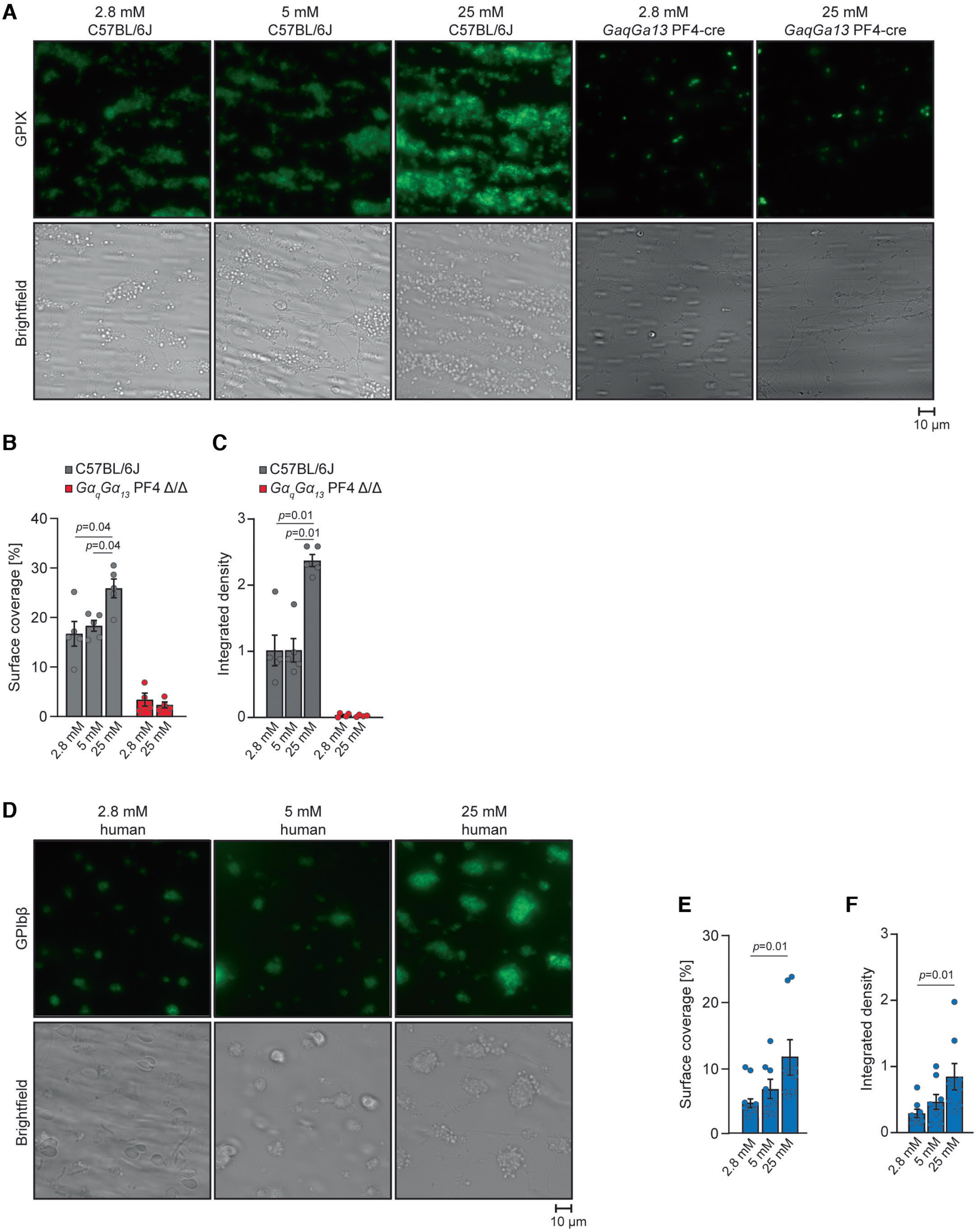

Figure 1.

◀ **Figure 1. Glucose promotes platelet activity.**

A Representative fluorescence and bright field microscopic images of platelet aggregates formed upon flow adhesion of anti-coagulated whole blood from 10-week-old male C57BL/6JRj and GαqGα13 PF4 Δ/Δ mice. Before perfusion, mouse blood was incubated for 5 min with 2.8, 5, or 25 mM glucose.

B, C Surface coverage (B) and integrated density (C) of platelets of conditions described in panels (A). 2.8, 5 and 25 mM C57BL/6JRj, $n = 5$; from five independent experiments; 2.8, 25 mM GαqGα13 PF4 Δ/Δ, $n = 4$; from four independent experiments. Each n represents a randomly taken image of the collagen-coated surface from different experiments.

D Representative fluorescence and bright field microscopic images of platelet aggregates formed upon flow adhesion of anti-coagulated whole blood from healthy humans. Before perfusion, blood was incubated for 5 min with 2.8, 5, or 25 mM glucose.

E, F Surface coverage (E) and integrated density (F) of platelets of conditions described in panels (D). 2.8, 5, and 25 mM human, $n = 8$; from eight independent experiments. Each n represents a randomly taken image of the collagen-coated surface from different experiments.

Data information: Kruskal–Wallis test followed by Mann–Whitney test as *post hoc* analysis with Benjamini–Hochberg correction for multiple comparisons. Data are mean ± SEM.
Source data are available online for this figure.

Taken together our data indicate that acute stimulation with glucose and yet unidentified factor derived from pancreatic β cells stimulates platelet reactivity. Moreover, our results suggest that a fraction of platelets pre-adhere to the endothelium of pancreatic islets.

To test the impact of long-term hyperglycemia on platelet reactivity, we isolated these cells from mice fed a high-fat diet (HFD) for 12 weeks. After this period of HFD feeding animals presents marked hyperglycemia and hyperlipidemia compared to mice fed a standard chow diet (El-Merahbi *et al*, 2020). HFD feeding did not affect, platelets count and size as well as the expression of platelet surface glycoproteins (Appendix Fig S1C–F). However, platelets isolated from HFD-fed mice presented reduced integrin exposure, but not P-selectin in response to the factors inducing blood coagulation (Appendix Fig S1G and H). In line with these findings, we observed that HFD feeding does not affect the localization of the platelets to the endothelium of pancreatic islets (Appendix Fig S1I and J). Three conclusions can be drawn from these data. First, platelets reside specifically in the microvasculature of the pancreatic islets. Second, acute stimulation with glucose activates platelets. Third, long-term hyperglycemia, which in the physiological settings is often accompanied by hyperlipidemia and hormonal imbalance does not affect platelet activity or localization.

Genetic ablation of major platelet functions results in glucose intolerance caused by decreased glucose-stimulated insulin secretion

Next, we tested if abrogation of key platelet functions can influence insulin secretion from pancreatic β cells. Initial platelet adhesion is mediated by the GPIb-IX-V complex, with the GPIbα subunit interacting with the von Willebrand factor (vWF) resulting in platelet tethering (Burkard *et al*, 2020). Mice deficient for GPIbα present profound defects in platelet adhesion, but also a severe platelet biogenesis defect resulting in macrothrombocytopenia (Kanaji *et al*, 2002). However, re-expression of engineered GPIbα protein, in which the extracellular domain is replaced by the α-subunit of human interleukin 4 receptor in GPIbα knockout mice (*Gp1ba*$^{-/-;TG}$), reverses the macrothrombocytopenia observed in GPIbα knockout mice but *Gp1ba*$^{-/-;TG}$ mice still present defective platelet adhesion (Bergmeier *et al*, 2006). Lethally irradiated wild-type mice were reconstituted either with wild-type or *Gp1ba*$^{-/-,TG}$ bone marrow. Among bone marrow-derived cells only megakaryocytes and platelets express GPIbα (Fujita *et al*, 1998). Mice deficient for the extracellular domain of GPIbα displayed glucose intolerance associated with reduced glucose-stimulated insulin levels (Fig 3A and B) while insulin sensitivity was not altered (Fig 3C). Next, we tested if the deletion of GPVI can alter glucose metabolism. Mice carrying a bone marrow-specific deletion of GPVI displayed glucose intolerance, and impaired insulin secretion but unaltered insulin sensitivity compared to controls (Fig 3D–F). These data indicated that chronic impairment in platelet adhesion results in glucose intolerance associated with declined insulin secretion. Platelet-restricted deletion of Gαq and Gα13 proteins (Wettschureck *et al*, 2001; Moers *et al*, 2003, 2004; Tiedt *et al*, 2007), causes a defect in platelet activation by major soluble agonists such as thrombin, ADP or thromboxane A2 (Fig EV1A) resulted in glucose intolerance and loss of glucose-stimulated insulin release whilst not affecting insulin sensitivity (Fig 3G–I). Similarly, circulating levels of C-peptide, which is co-secreted together with insulin, were markedly lower in the absence of Gαq and Gα13 proteins in the platelets (Fig EV3A and B). Altogether, these results indicate that activation of platelets is required for glucose-stimulated insulin secretion.

In the experiments described above we utilized male mice only. To test if the same mechanisms apply to females, we performed a glucose tolerance test on females carrying the platelet-restricted deletion of Gαq and Gα13 proteins. Genetic blockage of platelet activation in females resulted in a similar impairment of glucose tolerance as in males (Fig EV3C). These indicate that generally, platelets similarly regulate insulin secretion in both males and females. Altogether, these observations suggested that platelets promote pancreatic β cell function to maintain normoglycemia.

Platelets do not affect β cell mass

Lower insulin levels in mice showing defects in platelet function might be caused by a decrease in pancreatic β cells mass or defective insulin secretion. Measurements of the relative pancreatic β cell area in the mouse models with platelet dysfunction described above revealed no differences relative to controls (Fig EV3D–F). Moreover, insulin content in the pancreas was not altered in the absence of Gαq and Gα13 in platelets (Fig EV3G) and serum glucagon levels in these mice were not changed (Fig EV3H). Pancreatic islets isolated from mice deficient for Gαq and Gα13 in platelets, in the absence of the hormonal factors from circulation, presented a normal response to glucose, indicating that no developmental

 © 2023 The Authors

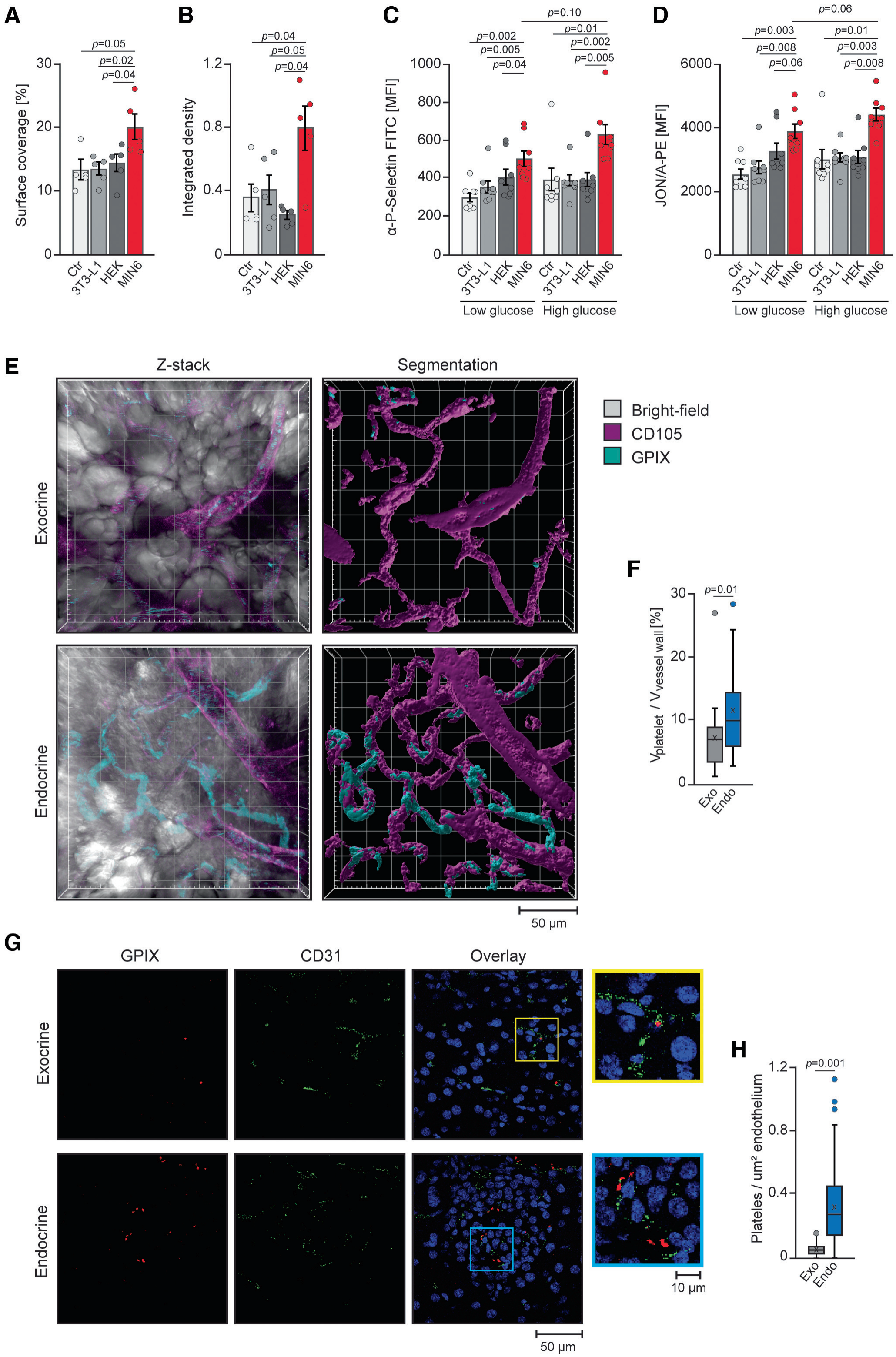

Figure 2.

defects are present as they are still fully functional if taken out of the context of the organism (Fig EV3I). Additionally, the body mass and body composition were not altered in mouse models of platelet dysfunction (Fig EV3J and K). Based on these findings, we hypothesize that platelets specifically promote insulin secretion from pancreatic β cells.

◀ **Figure 2. Humoral factors and glucose define reciprocal relation between platelets and pancreatic β cells.**

A, B Surface coverage (A) and integrated density (B) of whole blood thrombus from 10-week-old male C57BL/6JRj mice (n = 5). Before perfusion blood was incubated for 5 min with supernatants of indicated cells or control supernatant (Ctr). Supernatants from all the cell types indicated were generated in KRB containing 2.8 or 25 mM glucose. Each n represents the measurement of a sample from distinct mice.

C, D P-selectin exposure (C) and integrin activation (D) of platelets preincubated for 12 min with low glucose or high glucose (2.8 and 25 mM, respectively) supernatants of indicated cells or control supernatant (Ctr) determined by flow cytometry (n = 8). Each n represents the measurement of a sample from distinct mice.

E Representative z-stacks recorded *in vivo* of exocrine and endocrine C57BL/6JRj male mouse pancreas before and after segmentation.

F Volume of platelets attached to vasculature normalized to the volume of endothelial cells. Exocrine, n = 29; Endocrine, n = 26 (five C57BL/6JRj male mice). Each n represents one z-stack of randomly selected exocrine tissue or an islet.

G Representative DAPI and immunostainings of the exocrine and endocrine C57BL/6JRj male mouse pancreas.

H Platelet count normalized to the endothelial area. Exocrine, n = 131; Endocrine, n = 112 (six C57BL/6JRj male mice). Each n represents an image of randomly selected exocrine tissue or an islet.

Data information: Kruskal–Wallis test followed by Mann–Whitney test as *post hoc* analysis with Benjamini–Hochberg correction for multiple comparisons (A–D). Mann–Whitney test (F, H). Data are mean ± SEM. Data in boxplots: the center line shows median; cross indicates mean; box defines first and third quartiles; whiskers indicate 1.5 × interquartile range; outliers are individually plotted (F, H).
Source data are available online for this figure.

Pharmacological inhibition of platelet function and platelet depletion decreases insulin secretion and glucose tolerance

To test the hypothesis that platelets promote insulin secretion, we temporarily blocked different platelet functions using pharmacological tools in young-adult mice. JAQ1-IgG efficiently depleted GPVI from the surface of platelets, but did not affect platelet count (Fig EV4A and B). JAQ1-F$(ab)_2$ blocked GPVI while JON/A-F$(ab)_2$ abolished GPIIb/IIIa action (Fig EV4C). Application of the R300 antibody almost completely depleted mice from platelets (Fig EV4D). Importantly, GPVI depletion by JAQ1-IgG or GPVI-blockade by JAQ1-F$(ab)_2$, blockage of GPIIb/IIIa by JON/A-F$(ab)_2$, or systemic platelet depletion using R300 antibody also caused glucose intolerance in male mice resulting from decreased insulin secretion in response to glucose, while insulin sensitivity was not affected (Fig 4A–I). Similarly, the depletion of platelets in females resulted in glucose intolerance (Fig EV4E). Therefore, short-term blockage of platelet adhesion or platelet ablation results in similar glucose intolerance to long-term ablation of platelet function. Insulin promotes peripheral glucose uptake in the process initiated by its binding to the insulin receptor which causes activation of intracellular signaling cascade. AKT kinase represents a key-note signaling module activated by insulin stimulation (Hopkins *et al*, 2020). Of note, decreased insulin levels upon stimulation with glucose in mice depleted from platelets correlated with lower activity of AKT in skeletal muscles and perigonadal adipose tissue (Fig EV4F and G) but, the levels of glucose transporter 4 (Glut4), responsible for insulin-stimulated glucose transport in skeletal muscle and adipose tissue, were not affected in both of these organs by platelet depletion (Fig EV4F and G). Collectively, these data suggested that platelets stimulate insulin secretion in a para- or endocrine manner or by direct interaction with pancreatic β cells.

A platelet-derived factor directly stimulates insulin secretion

To test directly if platelets stimulate insulin secretion, we performed a co-culture experiment of platelets and the rat pancreatic β cell line, INS1. The presence of the platelets in the culture markedly increased insulin secretion, when incubated in the presence of low and high glucose (Fig 5A). To check if the effect of platelets on insulin secretion from β cells is mediated by a secreted factor, we prepared supernatants from activated human and mouse platelets (hPS and mPS, respectively). Stimulation of INS1 cells with hPS resulted in a dose-dependent increase in insulin secretion (Fig 5B). Stimulation of INS1 cells with hPS promoted insulin secretion in the presence of low and high glucose (Fig 5C). The effect of hPS on insulin secretion from INS1 cells persisted 2 and 8 h after the stimulation but was not observed following 16 h incubation of INS1 cells with hPS (Fig EV4H). Also, incubation of primary mouse islets and mouse insulinoma cells (MIN6) with mPS resulted in enhanced insulin secretion (Fig 5D and E). Next, we decided to extend our data from rodents to humans. Stimulation of the human insulin-releasing cell line EndoC-βH1 (Ravassard *et al*, 2011) or recently established human β cells derived from EndoC-βH5 pancreatic progenitors (Szczerbinska *et al*, 2022) with hPS resulted in increased insulin secretion (Figs 5F and EV4I). Taken together, these results indicated that platelets release factor(s) promoting insulin secretion.

Platelets release a lipid that promotes insulin secretion

In search of the substance(s) secreted by platelets that regulate(s) pancreatic β cell function, we subjected hPS to various biochemical modifications in order to identify the chemical nature of the platelet-secreted factor(s). To clean the hPS from all proteins and peptides, we treated them with Proteinase K. However, protein-free hPS still stimulated insulin secretion from β cells (Fig 6A). Next, we fractionated hPS for substances smaller and bigger than 3 kDa. Only substances smaller than 3 kDa present in hPS stimulated insulin secretion (Fig 6A). Dense granules of platelets, which are released during activation, contain several substances that potentially can influence pancreatic β cell function. These include purines (ATP and ADP), serotonin, and histamine (Nakamura *et al*, 2014; El-Merahbi *et al*, 2015; Fotino *et al*, 2018). We thus treated hPS with apyrase, monoamine oxidase (MAO), or diamine oxidase (DAO), to digest purines or degrade serotonin or histamine, respectively. These treatments did not influence the efficiency of hPS to induce insulin secretion (Fig 6A and B). Finally, we prepared lipid-depleted hPS and found that it did not stimulate insulin secretion (Fig 6C). Taken together, these data indicated that platelets stimulate insulin secretion by releasing a lipid mediator.

To identify the potential substance promoting insulin secretion that is present in the supernatant of activated platelets, we

 © 2023 The Authors

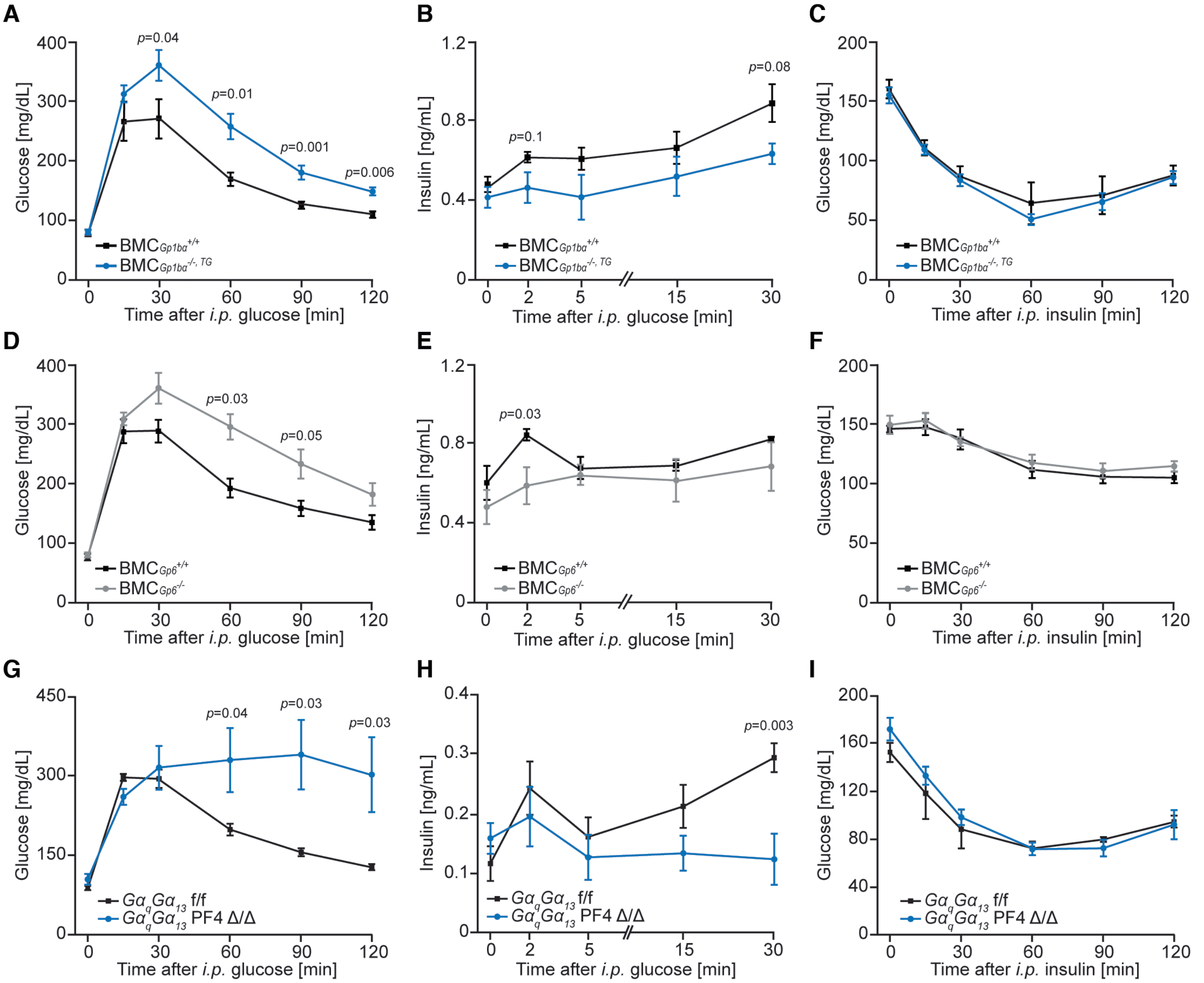

Figure 3. Genetic ablation of platelet functionality results in glucose intolerance caused by decreased glucose-stimulated insulin secretion.

A–I Glucose tolerance test (A, D, G), glucose-stimulated insulin secretion (B, E, H), and insulin tolerance test (C, F, I) of $Gp1b\alpha^{-/-,TG}$ bone marrow chimeric male mice (BMC) (A–C), $Gp6^{-/-}$ BMC male mice (D–F), and $G\alpha qG\alpha_{13}$ PF4 Δ/Δ male mice (G–I) with respective littermate control animals. For the glucose tolerance test, 2 g of glucose per kg of body weight (A, G) or 1.5 g of glucose per kg of body weight (D) and for glucose-stimulated insulin secretion, 3 g of glucose per kg of body weight (B, H) or 2 g of glucose per kg of body weight (E) was injected. For the insulin tolerance test, 0.5 U (C, I) or 0.25 U (F) of insulin per kg of body weight was injected. $BMC_{Gp1b\alpha}{}^{+/+}$, n = 8; $BMC_{Gp1b\alpha}{}^{-/-,TG}$, n = 10 (12 weeks old) (A). $BMC_{Gp1b\alpha}{}^{+/+}$, n = 6; $BMC_{Gp1b\alpha}{}^{-/-,TG}$, n = 8 (14 weeks old) (B). $BMC_{Gp1b\alpha}{}^{+/+}$, n = 5; $BMC_{Gp1b\alpha}{}^{-/-,TG}$, n = 8 (16 weeks old) (C). $BMC_{Gp6}{}^{+/+}$, n = 15; $BMC_{Gp6}{}^{-/-}$, n = 13 (14 weeks old) (D). $BMC_{Gp6}{}^{+/+}$, n = 6; $BMC_{Gp6}{}^{-/-}$, n = 6 (16 weeks old) (E). $BMC_{Gp6}{}^{+/+}$, n = 12; $BMC_{Gp6}{}^{-/-}$, n = 7 (18 weeks old) (F). $G\alpha qG\alpha_{13}$ f/f, n = 9; $G\alpha qG\alpha_{13}$ PF4 Δ/Δ, n = 6 (14 weeks old) (G). $G\alpha qG\alpha_{13}$ f/f, n = 12; $G\alpha qG\alpha_{13}$ PF4 Δ/Δ, n = 9 (16 weeks old) (H). $G\alpha qG\alpha_{13}$ f/f, n = 8; $G\alpha qG\alpha_{13}$ PF4 Δ/Δ, n = 7 (17 weeks old) (I).

Data information: Each n represents the measurement of a sample from distinct mice. Mann–Whitney test. Data are mean ± SEM.
Source data are available online for this figure.

performed semiquantitative lipidomic analyses of mPS and serum from platelet-depleted mice. Numerous lipids were present in the mPS (Fig 6D); one of them (HETEs) were significantly decreased in serum isolated from platelet-depleted mice. Members of the HETE family were previously established as regulators of multiple homeostatic functions including modulation of insulin secretion (Laychock, 1985; Turk *et al*, 1988). Most recently 20-HETE was shown to promote insulin secretion from pancreatic β cells (Tunaru *et al*, 2018). We confirmed that 20-HETE is present in the mPS and its levels are markedly reduced in the blood of mice depleted from platelets (Fig 6E and F). Of note, the depletion of platelets resulted in a similar reduction of 20-HETE levels to the treatment of mice with a selective CYP450 inhibitor (HET0016) required only for 20-HETE isomer production (Appendix Fig S2B). Moreover, 20-HETE

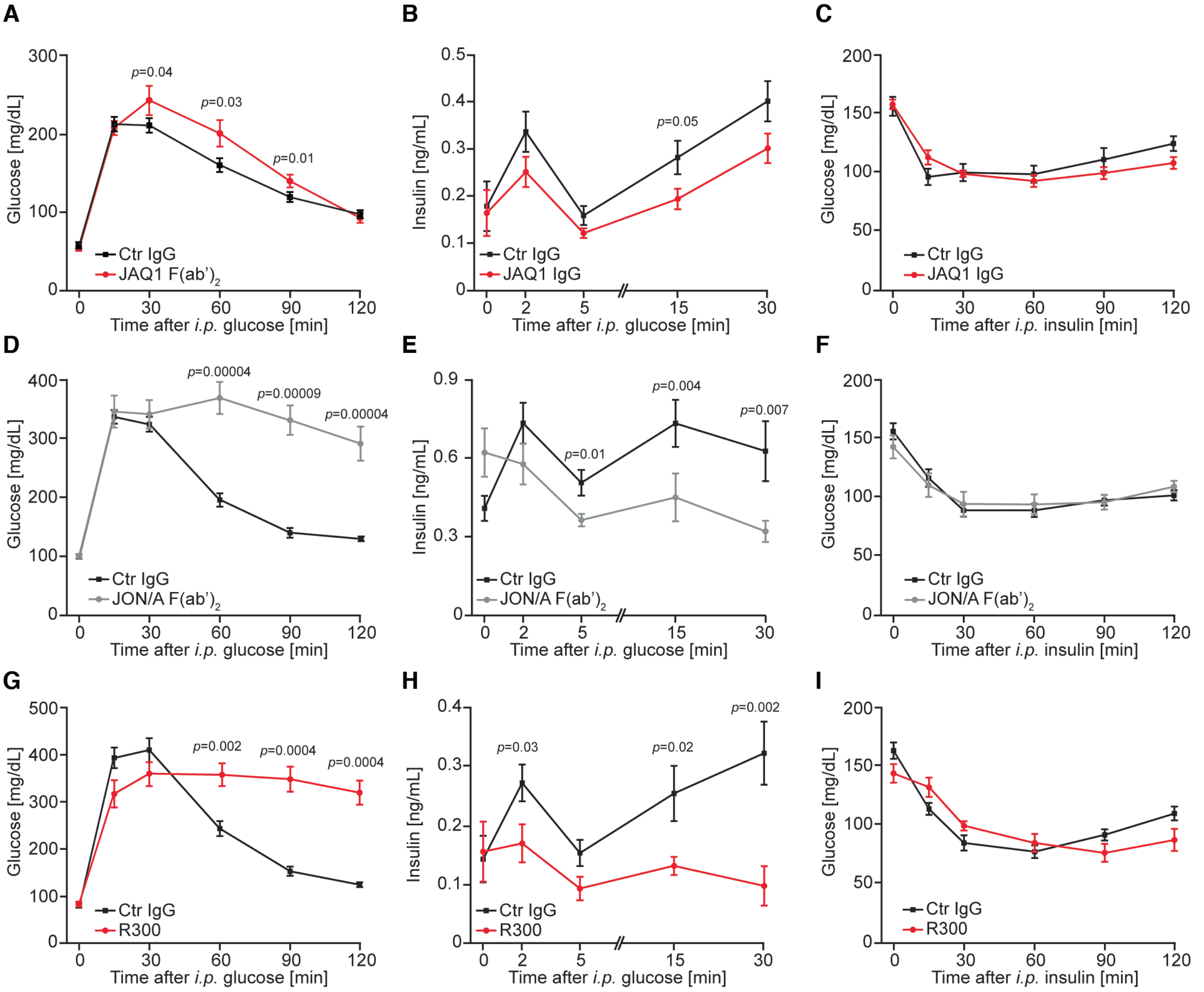

Figure 4. Pharmacological inhibition of platelet function and platelet depletion decrease insulin secretion and glucose tolerance.

A Glucose tolerance test of JAQ1 F(ab')$_2$ (n = 4) and control (Ctr) IgG injected (n = 9) C57BL/6JRj male mice.

B Glucose-stimulated insulin secretion of JAQ1 IgG (n = 11) and control IgG (n = 11) injected C57BL/6JRj male mice.

C Insulin tolerance test of JAQ1 IgG (n = 11) and control IgG injected (n = 7) C57BL/6JRj male mice.

D–F Glucose tolerance test (D), glucose-stimulated insulin secretion (E), and insulin tolerance test (F) of JON/A F(ab')$_2$ and control IgG injected C57BL/6JRj male mice. Ctr IgG, n = 8; JON/A F(ab')$_2$, n = 10 (D). Ctr IgG, n = 7; JON/A F(ab')$_2$, n = 10 (E). Ctr IgG, n = 8; JON/A-F(ab')$_2$, n = 7 (F).

G–I Glucose tolerance test (G), glucose-stimulated insulin secretion (H), and insulin tolerance test (I) of platelet depleted C57BL/6JRj male mice using R300 IgG (n = 9) and mice receiving control IgG (n = 9). Glucose tolerance test, glucose-stimulated insulin secretion, and insulin tolerance test were executed in the age of 6, 7, and 9 weeks, respectively.

Data information: Each n represents the measurement of a sample from distinct mice. Mann–Whitney test. Data are mean ± SEM.
Source data are available online for this figure.

secretion from platelets was increased in the presence of high glucose (Fig 6G). These data indicate that 20-HETE secreted by platelets might promote insulin secretion.

Platelet-derived 20-HETE promotes insulin secretion

Next, we tested if the effect of hPS on pancreatic β cells is mediated by a cell-surface receptor. We applied the Gαq/11 inhibitor YM-254890, which blocks signaling through GPCRs, prior to the stimulation of pancreatic β cells with hPS. This completely abrogated PS-stimulated insulin secretion (Fig 7A), indicating that a factor released by platelets acts on insulin-producing cells through a GPCR. The previous study by Tunaru *et al* (2018) suggested that 20-HETE acts as one of the Free Fatty Acid Receptor 1 (FFAR1) (also known as G-protein-coupled receptor 40 (GPR40)) agonists. To test if FFAR1 was responsible, at least partially, for platelet-induced

© 2023 The Authors

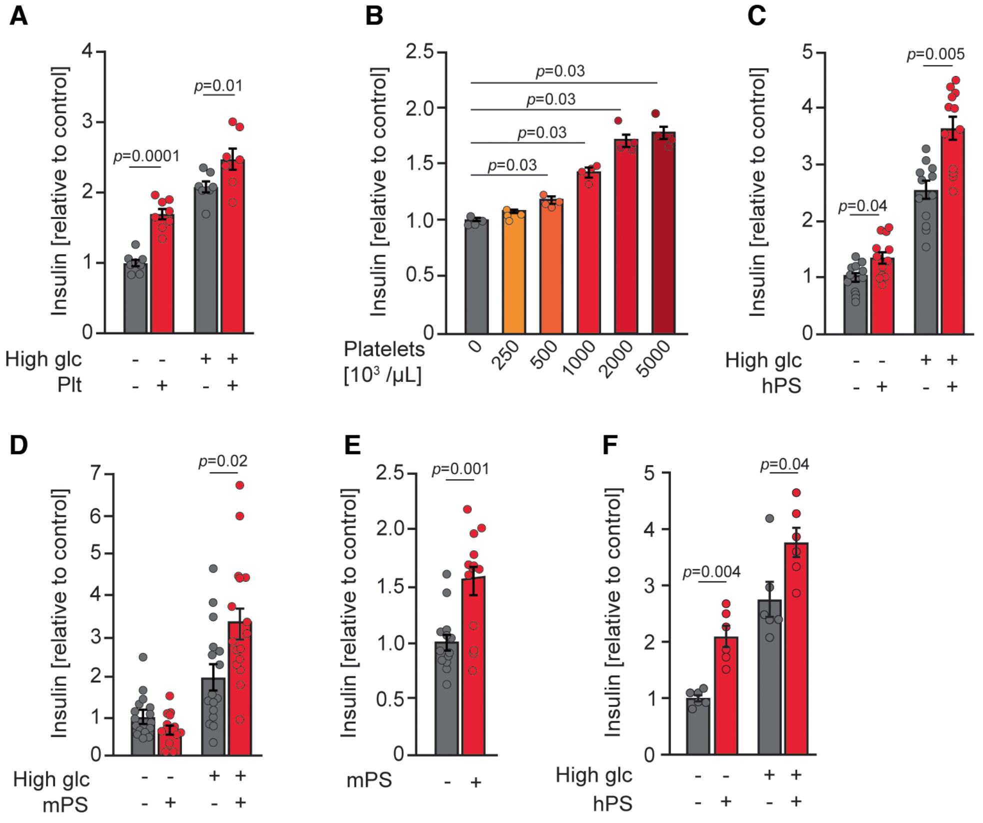

Figure 5. A platelet-derived factor directly stimulates insulin secretion.

A Relative insulin secretion of INS1 cells co-cultured with human platelets (Plt) or control buffer (Ctr) at low (2.8 mM) or high (15 mM) glucose for 30 min. 2.8 mM, n = 8; 15 mM, n = 7.

B Relative insulin secretion of INS1 cells stimulated with supernatant of different concentrations of activated human platelets in the presence of 15 mM glucose. n = 4.

C Relative insulin secretion of INS1 cells stimulated with supernatant of activated human platelets (hPS) or control buffer upon low (2.8 mM) and high (15 mM) glucose. n = 12.

D Relative insulin secretion of isolated islets from C57BL/6JRj male mice after 90 min of stimulation with supernatant of activated mouse platelets (mPS) or control buffer. Stimulation occurred during low (2.8 mM) or high (16.7 mM) glucose conditions. (−) High glc, (−) mPS, n = 16; (+) High glc, (−) mPS, n = 16; (−) High glc, (+) mPS, n = 14; (+) High glc, (+) mPS, n = 16.

E Relative insulin secretion of MIN6 cells stimulated with supernatant of activated mouse platelets (mPS) or control buffer upon 25 mM glucose. n = 13.

F Relative insulin secretion of human EndoC-βH5 cells stimulated with supernatant of activated human platelets (hPS) upon low (2.8 mM), or high (20 mM) glucose (n = 6).

Data information: Each n represents an independent biological replicate. Data have a normal distribution (A and E). Data distribution was checked by the Shapiro–Wilk normality test. Unpaired t-test (E). One-way ANOVA followed by Sidak's multiple comparisons test (A). Kruskal–Wallis test followed by Mann–Whitney test as *post hoc* analysis with Benjamini–Hochberg correction for multiple comparisons (B–D, F). Data are mean ± SEM.
Source data are available online for this figure.

insulin secretion, we applied the FFAR1 antagonist GW1100 to hPS-stimulated pancreatic β cells. This treatment reduced basal insulin secretion but also partially reduced hPS-stimulated insulin secretion (Fig 7A), indicating that platelets stimulate insulin secretion by utilizing a factor that at least partially acts via FFAR1. Consistently, siRNA-mediated silencing of FFAR1 (Appendix Fig S2A) partially lowered insulin secretion in response to the stimulation with hPS (Fig 7B). However, the partial effect of hPS on insulin secretion in the absence of FFAR1 suggests that alternative mechanisms mediating the platelet effect on β cells exist. Injection of HET0016, a selective CYP450 inhibitor required for 20-HETE production, for 4 consecutive days in wild-type mice, resulted in an approximately 70% reduction in serum levels of 20-HETE which was associated with glucose intolerance and reduced glucose-stimulated insulin secretion (Appendix Fig S2B and C, Fig 7C), but did not alter the morphology of pancreatic islets (Appendix Fig S2D and E). Of note, the application of HET0016 to mice depleted from platelets did not further reduce glucose tolerance (Fig 7D). Previous study indicates that 20-HETE is also produced by pancreatic β cells (Tunaru *et al*, 2018). To test if inhibition of β cell-derived 20-HETE production will affect platelet-stimulated insulin production, we pre-treated INS1 cells with HET0016 and then stimulated these cells with hPS. Pre-treatment of INS1 cells with HET0016 did not abolish the effect of hPS on insulin secretion (Fig 7E). Activation of the FFAR1 receptor commonly elevates Ca^{2+} concentration [Ca^{2+}] in β cells (Schnell *et al*, 2007; Vettor *et al*, 2008; Usui *et al*, 2019). In line with this, stimulation of INS1 cells with hPS potentiated the glucose-induced Ca^{2+} influx (Fig 7F). Previous studies indicated that activation of

© 2023 The Authors

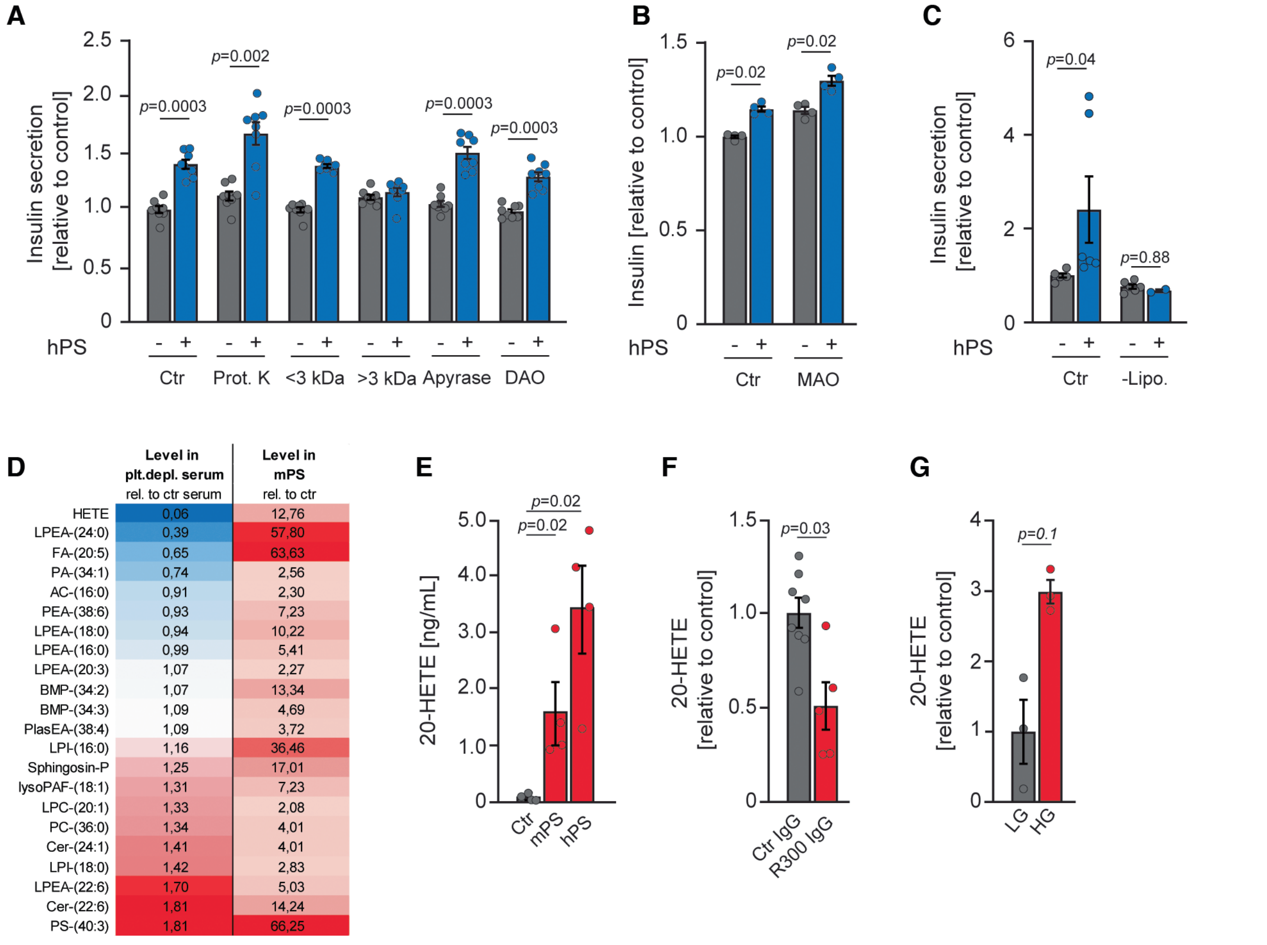

Figure 6. Platelets release a lipid that promotes insulin secretion.

A Relative insulin secretion of INS1 cells stimulated with unmodified (Ctr), proteinase K treated, fractionated (< 3 kDa, > 3 kDa), apyrase or diamine oxidase (DAO) treated supernatant of activated human platelets (hPS) in the presence of 15 mM glucose (n = 8).

B Relative insulin secretion of INS1 cells stimulated with unmodified (Ctr) or monoamine oxidase A (MAO) treated supernatant of activated human platelets (hPS) in the presence of 15 mM glucose (n = 4).

C Relative insulin secretion of INS1 cells stimulated with unmodified (Ctr) or lipophilic fraction extracted (-Lipo.) supernatant of activated human platelets (hPS) in the presence of 15 mM glucose. Ctr, n = 6.

D Liquid chromatography-mass spectrometry analysis of supernatant from activated mouse platelets (mPS) and serum of platelet-depleted mice using R300 IgG. The result is shown as relative values to the respective control supernatant (ctr) and serum of control IgG-treated mice (ctr serum) (n = 4). The intensity of the blue color indicates a lower presence of lipids in the serum of platelet-depleted mice, while the intensity of the red color indicates a higher presence.

E Levels of 20-HETE in the supernatant of activated mouse and human platelets (mPS, hPS) and control supernatant (Ctr) (n = 4).

F Serum levels of 20-HETE from platelet-depleted 8-week-old male C57BL/6JRj mice using R300 IgG and mice receiving control (Ctr) IgG (n = 4).

G Levels of 20-HETE in the supernatant of human platelets stimulated with collagen-related protein in low (5 mM) or high (25 mM) glucose (n = 3).

Data information: Each n represents an independent biological replicate (A–E, G) or measurement of a sample from distinct mice (F). Mann–Whitney test (F, G). Kruskal–Wallis test followed by Mann–Whitney test as *post hoc* analysis with Benjamini–Hochberg correction for multiple comparisons (A–C, E) Data are mean ± SEM.
Source data are available online for this figure.

FFAR1 stimulates Protein Kinase D (PKD) activity (Ferdaoussi *et al*, 2012), a master regulator of glucose homeostasis (Sumara *et al*, 2009; Löffler *et al*, 2018; Mayer *et al*, 2019; Kolczynska *et al*, 2020; Trujillo-Viera *et al*, 2021) and insulin secretion (Sumara *et al*, 2009). PKD family of kinases consists of three members (PKD1, PKD2, and PKD3) (Kolczynska *et al*, 2020). As indicated by an antibody that recognizes phosphorylation of s916 on PKD1 and s876 on PKD2 as well as an antibody that recognizes s744 and 748 in all PKD members (Trujillo-Viera *et al*, 2021), the activity of PKD was increased in response to the stimulation with hPS (Fig 7G). Consistently, using an antibody that recognizes phosphorylation motive in PKD's substrate proteins (Loza-Valdes *et al*, 2021), we confirmed the increased activity of these kinases in β cells in response to hPS stimulation (Fig 7G). Finally, inhibition of PKDs with the specific inhibitor (CRT 0066101) in β cells abrogated hPS-induced insulin secretion (Fig 7H).

© 2023 The Authors

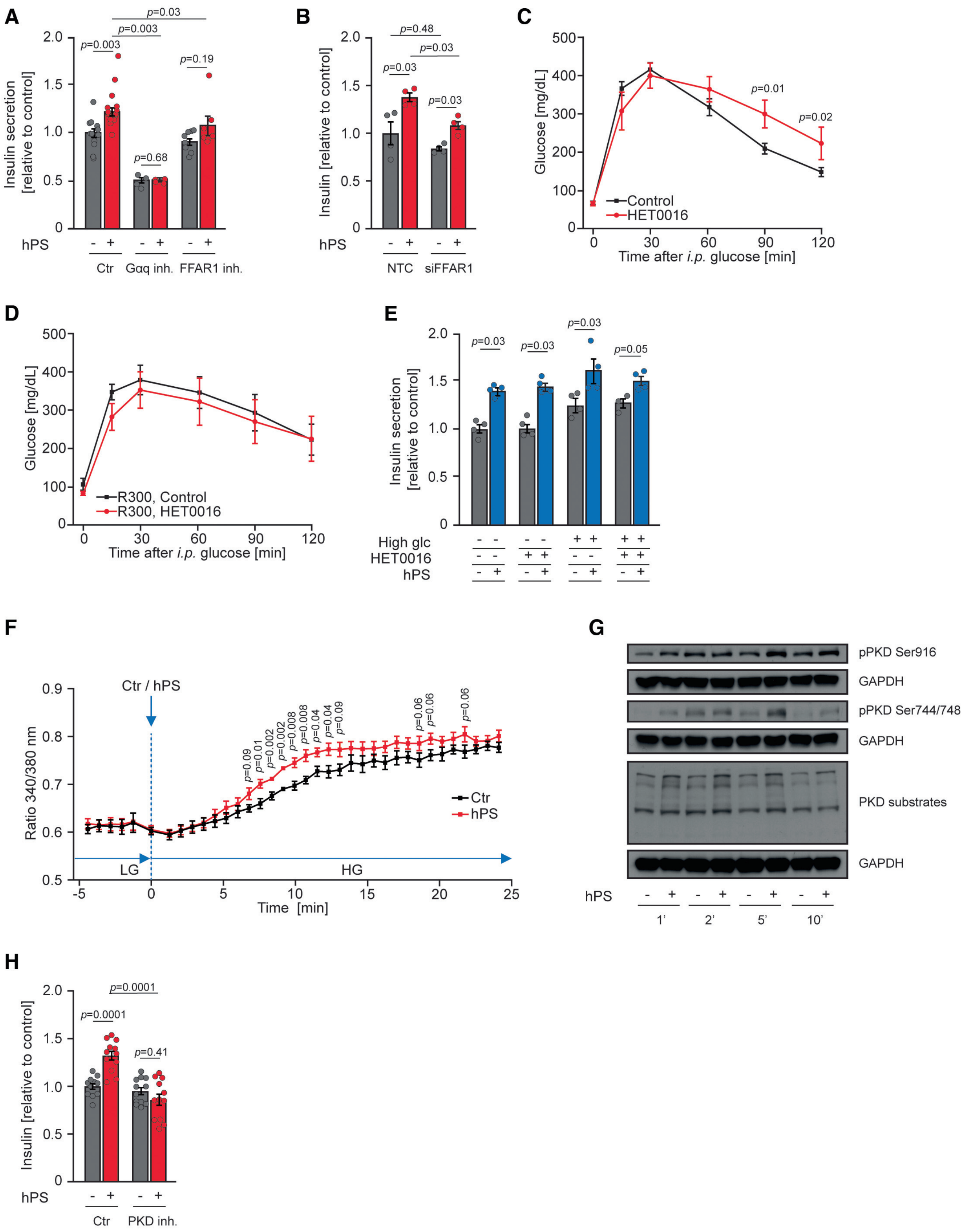

Figure 7.

Altogether, these data indicate that platelets release 20-HETE, which stimulates FFAR1 in β cells. This promotes Ca^{2+} influx as well as activation of PKD finally leading to elevated insulin secretion.

The impact of platelets on insulin secretion declines with age

The anti-thrombotic drug clopidogrel blocks platelet activation by targeting the ADP receptor $P2Y_{12}$ (Savi & Herbert, 2005). Three

Figure 7. Platelet-derived 20-HETE promotes insulin secretion.

A Insulin secretion of INS1 cells stimulated with supernatant of activated human platelets (hPS) or control supernatant. INS1 cells were used untreated (Ctr) or treated with Gαq/11 inhibitor YM-254890 (5 nM) or FFAR1 antagonist GW1100 (5 μM). Ctr, (−) hPS, n = 12; Ctr, (+) hPS, n = 16; Gαq inh., (−) hPS, n = 4, Gαq inh., (+) hPS, n = 4; FFAR1 inh., (−) hPS, n = 8; FFAR1 inh., (+) hPS, n = 7.

B Insulin secretion of INS1 depleted from FFAR1 using siRNA or control cells transfected with non-targeting control (NTC) siRNA and stimulated with supernatant of activated human platelets (hPS) or control supernatant (n = 4).

C, D Glucose tolerance test (2 g of glucose per kg of body weight) of C57BL/6JRj mice injected i.p. with HET0016 (10 mg per kg of body weight) 72 h, 48 h, 24 h, and 30 min before the experiment with respective control. Control, n = 8; HET0016, n = 6. Mice were additionally treated with platelet depletion R300 IgG (2 mg per kg of body weight) or control IgG (D). R300, Control, n = 6; R300, HET0016, n = 6.

E Insulin secretion from INS1 cells pre-treated with HET0016 (30 μM) for 3 h and stimulated with hPS for 20 min in low (2.8 mM) and high (15 mM) glucose (n = 4).

F Calcium influx was measured in INS1 cells upon stimulation with activated human platelets (hPS) or control supernatant (Ctr) in the presence of 2.8 mM (LG) or 15 mM glucose (HG) using Fura-2 calcium tracer (n = 6).

G Western Blot (WB) for indicated proteins on extracts from INS1 cells stimulated with activated human platelets (hPS) or control supernatant in the presence of 15 mM glucose for indicated time points. Representative image from four independent experiments.

H Insulin secretion of PKD inhibitor (CRT0066101, 10 μM) treated INS1 cells with control (Ctr) that were stimulated with supernatant of activated human platelets (hPS) or control supernatant (Ctr) in the presence of 15 mM glucose. Ctr, (−) hPS, n = 11; Ctr, (+) hPS, n = 12; PKD inh., (−) hPS, n = 10; PKD inh., (+) hPS, n = 12.

Data information: Each n represents an independent biological replicate (A, B, E–H) or measurement of a sample from distinct mice (C, D). Data have a normal distribution (A and E). Data distribution was checked by the Shapiro–Wilk normality test. One-way ANOVA followed by Sidak's multiple comparisons test (H). Mann–Whitney test (C, D, F). Kruskal–Wallis test followed by Mann–Whitney test as *post hoc* analysis with Benjamini–Hochberg correction for multiple comparisons (A, B, E). Data are mean ± SEM.
Source data are available online for this figure.

weeks of clopidogrel treatment of young-adult male mice resulted in glucose intolerance and diminished insulin secretion upon glucose stimulation but did not affect insulin sensitivity (Figs 8A and B, and EV5A). Of note, the treatment of mice with clopidogrel did not affect the abundance of glucose transporter 1 and 3 (Glut1 and Glut3) (Fig EV5B). Similarly, in young adult females, 3 weeks of treatment with clopidogrel resulted in glucose intolerance (Fig EV5C). Moreover, to confirm that observed phenomena are conserved across species we implemented a rat model of platelet dysfunction. Treatment of young-adult rats with clopidogrel also resulted in glucose intolerance, and lower glucose-stimulated insulin secretion, but did not affect insulin sensitivity (Fig EV5D–F). In clinical practice, clopidogrel has been used for decades (Savi & Herbert, 2005). To our knowledge, glucose intolerance is not a commonly reported side effect of clopidogrel. The vast majority of patients receiving clopidogrel are older than 60 years of age (Savi & Herbert, 2005). This prompted us to investigate the effect of clopidogrel on aged mice. Interestingly, in aged animals, clopidogrel did not evoke glucose intolerance nor decreased insulin levels (Fig 8C and D). Of note, the treatment of young adult and aged mice with clopidogrel was equally efficient in the reduction in agonist induced platelet activation (Fig EV5G). As mentioned before, platelet depletion in young-adult mice leads to glucose intolerance caused by decreased glucose-stimulated insulin secretion (Fig 4G and H). However, platelet depletion in aged mice using the R300 antibody did not result in glucose intolerance (Fig 8E). Of note, the depletion of platelets in mice fed HFD only marginally decreased glucose tolerance (Fig EV5H), while administration of clopidogrel in animals fed HFD did not affect glucose levels (Fig EV5I). In line with these observations mice carrying a platelet-restricted deletion of Gαq and Gα13 proteins did not present altered glucose tolerance when fed HFD (Fig EV5J).

As 20-HETE represents a platelet-derived factor that stimulates insulin secretion, we measured the levels of 20-HETE in young and aged mice. Of note, blood levels of 20-HETE were drastically decreased in aged animals (Fig 8F). Moreover, secretion of 20-HETE from platelets isolated from aged mice was markedly decreased compared to the secretome of platelets isolated from young adult animals (Fig 8G) Similarly, levels of 20-HETE decreased in aged humans (Fig 8H).

Collectively, these results demonstrate that platelets in young subjects promote insulin levels by secreting 20-HETE, while the capacity of platelets to stimulate insulin secretion declines with age.

Discussion

Platelets are critical mediators of hemostasis that are increasingly recognized to have central functions also in the regulation of inflammatory response, vascular integrity, regenerative processes, and cancer progression (Holinstat, 2017; Mancuso & Santagostino, 2017; Deppermann, 2018; Ho-Tin-Noé *et al*, 2018; Malehmir *et al*, 2019; Burkard *et al*, 2020; Levoux *et al*, 2021).

Platelet function is altered in the course of type 1 and type 2 diabetes, indicating that hyperglycemia might directly influence platelet reactivity (Malachowska *et al*, 2015). Here we showed that high glucose levels promote the activation of platelets, which is in line with previous work of others, demonstrating that several mechanisms contribute to this effect, including a change of osmolality and increased glucose metabolism of platelets leading to elevated levels of reactive oxygen species (Sudic *et al*, 2006; Tang *et al*, 2011; Fidler *et al*, 2019). Our data also point to a novel mechanism that can contribute to the increased reactivity of platelets during hyperglycemia. Namely, we showed that pancreatic β cells, especially, in combination with high glucose levels increase platelet reactivity. In line with this notion, we show that a fraction of platelets adheres to the endothelium of pancreatic islets. The mechanism mediating the limited, and presumably transient, activation of platelets upon exposure to pancreatic β cells remains unclear. Insulin is the major factor secreted from β cells upon exposure to glucose, but previous studies indicate that insulin attenuates rather than stimulates platelet activity (Ferreira *et al*, 2004). Nevertheless, insulin granules also contain potent activators of platelets, like ATP and ADP, which are released together with insulin (Richards-Williams *et al*, 2008; Burkard *et al*, 2020). Interestingly, the endo-, and exocrine pancreas present substantial differences in composition and abundance of collagen fibers

 © 2023 The Authors

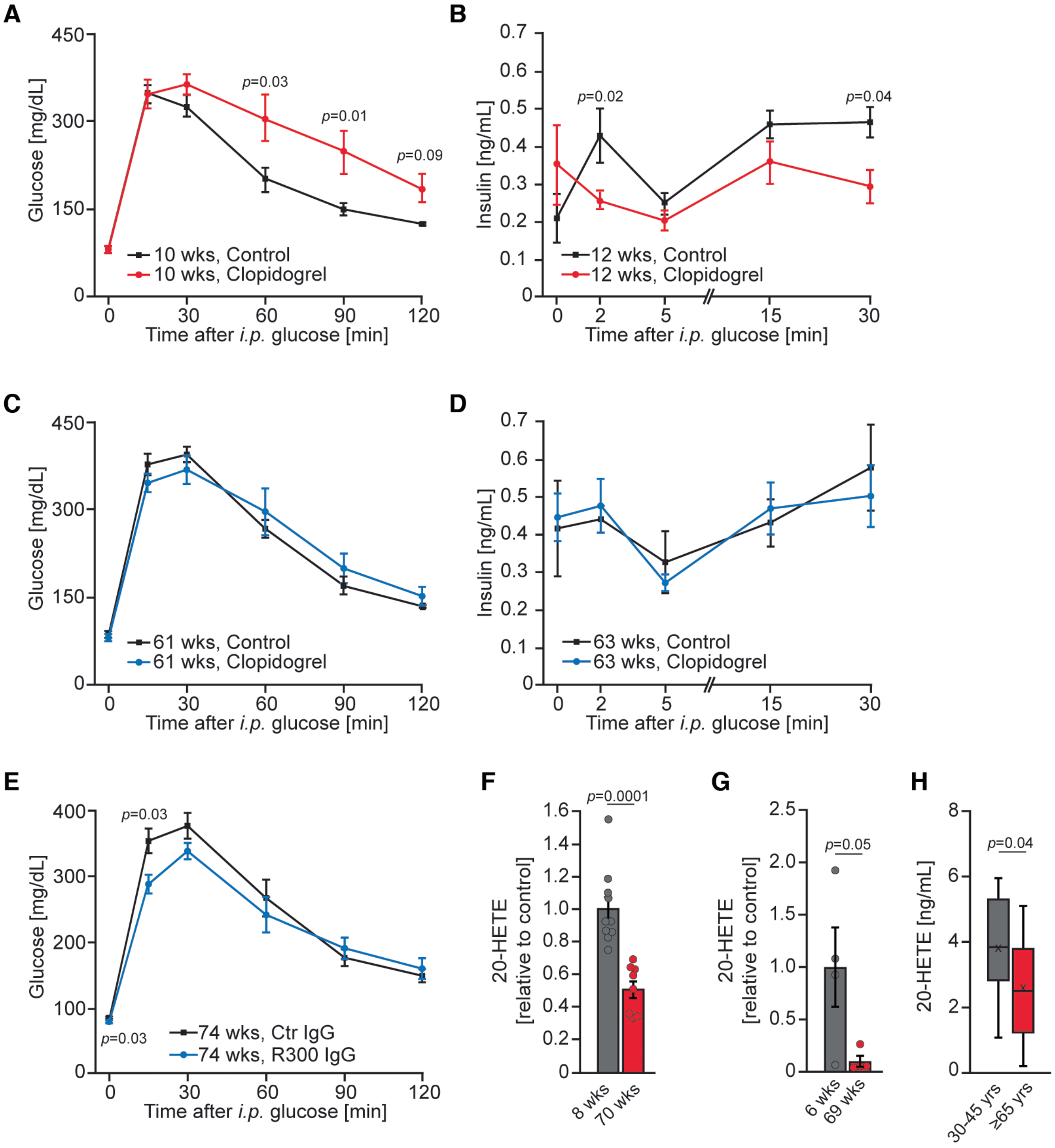

Figure 8. The impact of platelets on insulin secretion declines with age.

A Glucose tolerance test (2 g of glucose per kg of body weight) of 10-week-old C57BL/6JRj male mice treated with clopidogrel for 3 weeks with respective control animals ($n = 7$).

B Glucose-stimulated insulin secretion (3 g of glucose per kg of body weight) of 12-week-old C57BL/6JRj male mice treated with clopidogrel for 5 weeks with respective control mice ($n = 7$).

C Glucose tolerance test (2 g of glucose per kg of body weight) of 61-week-old C57BL/6JRj male mice treated with clopidogrel for 3 weeks with respective control animals ($n = 7$).

D Glucose-stimulated insulin secretion (3 g of glucose per kg of body weight) of 63-week-old C57BL/6JRj male mice treated with clopidogrel for 5 weeks with respective control mice ($n = 7$).

E Glucose tolerance test (2 g of glucose per kg of body weight) of 74-week-old platelet-depleted C57BL/6JRj male mice using R300 IgG and mice receiving control IgG ($n = 5$).

F Relative serum levels of 20-HETE from 8 and 70 weeks old C57BL/6JRj mice. Eight weeks, $n = 10$; 70 weeks, $n = 8$.

G Levels of 20-HETE in supernatants from activated mouse platelets isolated from young (6 weeks old) and aged (69 weeks old) C57BL/6JRj male mice ($n = 4$).

H Serum levels of 20-HETE from 30 to 45 years and 65 to 79-year-old human. < 45 years, $n = 16$; ≥ 65 years, $n = 9$.

Data information: Each n represents the measurement of a sample from distinct mice (A–G) or humans (H). Data have normal distribution (F, H). Data distribution was checked by the Shapiro–Wilk normality test. Unpaired t-test (F, H). Mann–Whitney test (A–E, G). Data are mean ± SEM (A–G). Data in boxplots: the center line shows median; cross indicates mean; box defines first and third quartiles; whiskers indicate 1.5 × interquartile range; outliers are individually plotted (H).
Source data are available online for this figure.

© 2023 The Authors

(Huang *et al*, 2011), which might influence platelet adhesion. Also, the specific organization of islets vasculature might determine the abundance of the platelets in pancreatic islets. Vascular density is approximately 10 times higher in the endo- versus exocrine pancreas, the same applies to the density of fenestration. This correlates with a larger blood supply to the endocrine pancreas which receives about 15% of the total blood volume pumped to the pancreas, even though islets constitute 1–2% of the total mass of the pancreas (Hogan & Hull, 2017). It has been proposed that dysfunction of islets endothelium contributes to β cell failure during the development of diabetes (Hogan & Hull, 2017). The specific composition of the vasculature in the pancreatic islets suggests that endothelial cells from the endocrine pancreas possess special features (manifested also at the molecular level) distinct from the exocrine part of the pancreas and perhaps other tissues. However, despite clear morphological differences between endothelial cells from the endo- and exocrine pancreas little is known about molecular features that distinguish these populations of cells. Endothelial cells at different anatomical locations express many proteins that can modulate the adhesion of platelets. This includes thrombomodulin (CD141), von Willebrand factor (vWF), coagulation factor V, and tissue factor (TF) (Siegel-Axel & Gawaz, 2007). To our knowledge, the relative abundance of these proteins in the endothelium of the endo- and exocrine pancreas have not been investigated so far. Recently, transcriptional profiles of human endothelial cells from the endo- and exocrine pancreas were determined. More than 1,200 genes were differentially expressed in these populations of cells. Several functional gene sets were enriched in endothelial cell populations isolated either from the endo- or exocrine pancreas (Jonsson *et al*, 2020). However, based on existing data is not possible to predict if differences between molecular signatures between endothelial cells from the endo- and exocrine pancreas contributing to platelets adhesion in the vasculature of islets. Further detailed studies will be required to unravel the mechanisms underlying the localization of platelets to the endothelium of pancreatic islets as well as the enhanced activity of platelets upon exposure of β cells.

Our results provide strong evidence for an unexpected and evolutionarily conserved function of platelets in the regulation of insulin release and glucose homeostasis. Genetic or pharmacological interference with major platelet adhesion mechanisms consistently resulted in a reduction of insulin secretion and thus glucose intolerance. Importantly, we showed that observed phenomena are independent of gender. Insulin granule release from β cells in response to glucose challenge is modulated by multiple metabolites as well as auto-, para-, and endocrine factors (Ashcroft & Rorsman, 2012). Several of these hormones and factors are stored in dense, and α granules of platelets making them potential modulators of pancreatic β cell function and energy homeostasis (Sumara *et al*, 2012; Rorsman & Braun, 2013; El-Merahbi *et al*, 2015; Manne *et al*, 2017). Our data provide the first direct evidence that platelets promote β cell function by secreting an insulinotropic factor. Our further analyses revealed that none of the known factors, which are stored in the granules of platelets (serotonin, ATP, ADP, multiple peptide-hormones), but rather lipid-based substances mediate this unexpected effect of platelets on insulin secretion. Four of the lipid molecules (or class of lipids) were especially interesting in the context of our study: HETE, LPEA-(24:0), FA-(20:5), and PA-(34:1), all of which were enriched in mPS whereas their levels were markedly reduced in the blood of platelet depleted mice. We focused our study on the HETE group of lipid mediators as previous work by others had established multiple factors from this group as the mediators of several homeostatic functions including modulation of pancreatic β cells action (Laychock, 1985; Turk *et al*, 1988). One member of the HETE group, 20-HETE was recently found as an insulinotropic factor (Tunaru *et al*, 2018) and indeed platelet depletion markedly reduces 20-HETE levels in the circulation. However, it has been suggested that 20-HETE is produced locally in pancreatic islets and stimulates insulin secretion in an autocrine manner (Tunaru *et al*, 2018). We cannot precisely estimate the relative contribution of 20-HETE produced by islets and by platelets to the stimulation of insulin secretion. However, blood levels of 20-HETE were reduced by about 50% in platelet-depleted mice. Most importantly, inhibition of 20-HETE production in platelet-depleted mice had virtually no effect on glucose tolerance, indicating that platelet-derived 20-HETE plays a predominant role in the stimulation of insulin secretion. Nevertheless, the question of whether 20-HETE derived from platelets acts in the para-, endocrine manner remains open. The fact that platelets localize to the endothelium of pancreatic islets might indicate that the paracrine mode of action is predominant. To resolve this question, mouse models in which platelet adhesion to the vasculature of islets is diminished, but production of 20-HETE is not altered should be investigated in detail. Our data indicate also that platelets release several other factors that potentially can influence insulin secretion. Previous studies indicate that 5-HETE, and 12-HETE which are abundantly produced by platelets, can stimulate insulin secretion by cell surface-mediated signaling as well as induction of lipid metabolism in the β cells (Laychock, 1985; Turk *et al*, 1988). At this stage, we cannot exclude that platelets modulate β cell function also by utilizing other members of the HETE family.

Silencing or inhibition of the 20-HETE receptor, FFAR1 (Tunaru *et al*, 2018), in β cells significantly, but not completely, reduced platelet-evoked insulin secretion. These observations suggest that platelets secrete another insulinotropic factor, or 20-HETE does not solely act on FFAR1. Depletion of lipids from hPS completely abrogated its effect on β cells, suggesting that other factors released by platelets might also be lipid-based. Our lipidomic analysis identified a couple of putative factors which should be investigated in the future. Also, the mechanism by which platelets release 20-HETE and other lipids stimulating β cell function remains unclear. These findings open a plethora of new research directions in the future.

Anti-platelet agents have been in clinical use to prevent or treat acutely ischemic cardiovascular events, such as myocardial infarction. Among them, $P2Y_{12}$ ADP receptor antagonists such as clopidogrel are commonly used as an anti-platelet therapy (Savi & Herbert, 2005). The effect of the $P2Y_{12}$ ADP receptor blockers such as clopidogrel is confined to reducing platelet reactivity (Savi & Herbert, 2005). We showed that in young-adult mice clopidogrel treatment resulted in glucose intolerance caused by decreased glucose-stimulated insulin secretion. However, to our knowledge glucose intolerance has not been reported so far in patients receiving $P2Y_{12}$ ADP receptor blockers, including clopidogrel. Clopidogrel is mainly used to prevent ischemic cardiovascular events, a clinical complication that typically occurs in aged patients. Therefore, we tested the effect of clopidogrel treatment on glucose tolerance and insulin levels in aged mice. Strikingly, in 14–17-month-old animals, clopidogrel had no effect on insulin and glucose levels, and also platelet

© 2023 The Authors

depletion had no effect on glucose homeostasis in those animals. These results indicate that the abundance of insulinotropic factors released by platelets might decrease with age. In fact, we showed that levels of serum 20-HETE decrease with age in mice and humans. However, mechanisms mediating decreased production of 20-HETE during aging remain unclear. Also, other putative insulinotropic factors released by platelets and/or platelet localization to the pancreatic islets vasculature might decline with aging. Also feeding animals with HFD, which induces hyperglycemia and hyperlipidemia results in reduced platelet action on pancreatic β cells. One possible explanation is that during HFD significant increase in fatty acid levels in circulation occurs (Wit *et al*, 2022), this might unspecifically saturate FFAR1 independently of 20-HETE derived from platelets.

Numerous studies indicate that blood components might determine systemic aging of the organism (Conboy *et al*, 2005; Villeda *et al*, 2011; Katsimpardi *et al*, 2014; Baht *et al*, 2015). Of note, significant changes in platelet reactivity and their redox homeostasis during aging have been observed (Jain *et al*, 2019). Although, experimental induction of senescence in β cells of mice enhances insulin secretion (Helman *et al*, 2016), generally with age the responsiveness of pancreatic β cells to glucose declines (Rorsman & Braun, 2013). Based on these findings it may be tempting to speculate that declining levels of platelet-derived 20-HETE (and possibly other platelet-derived insulinotropic factors) could potentially contribute to the age-related decline in pancreatic β cell fitness. However, it should be noted that other factors, such as mentioned changes in platelet reactivity which are manifested during aging (Jain *et al*, 2019) should be considered as potential factors contributing to the decrease action of platelets on pancreatic β cells.

The findings reported here highlight a novel and unexpected role of platelets in the regulation of pancreatic β cell function and glucose metabolism. These findings may serve as a basis for future studies on the local and systemic effects of 20-HETE and possibly other insulinotropic factors/s released by platelets but also on the molecular mechanisms underlying the selective interaction of platelets with the endothelium of the endocrine pancreas. Evolutionary conservation of this unanticipated platelet function may open in future new therapeutic avenues for the treatment of patients with perturbed glucose homeostasis, especially in the context of age-related decline in pancreatic β cell function.

Materials and Methods

Animals

Gp6$^{-/-}$, and *Gp1bα*$^{-/-}$ mice were described previously (Kanaji *et al*, 2002; Bender *et al*, 2013). Gαq$^{fl/fl}$ and Gα13$^{fl/fl}$ mice (Wettschureck *et al*, 2001; Moers *et al*, 2003) (kindly contributed by Prof. Dr. Stefan Offermanns and Shaun R Coughlin) were crossed with PF4-Cre mice (Tiedt *et al*, 2007) and then intercrossed to obtain PF4-Cre *Gαq*$^{fl/fl}$ *Gα13*$^{fl/fl}$ double knock out mice. For all experiments, respective littermate control mice were used (Gp6$^{+/+}$, and Gp1bα$^{+/+}$, Gαq$^{fl/fl}$ Gα13$^{fl/fl}$). Wild-type C57BL/6JRj mice and WISTAR rats were obtained from Janvier Labs (Le Genest-Saint-Isle, France). Male and female mice and male rats were used in the experiments. Mice and rats were held under specific pathogen-free conditions in the animal facility of the Rudolf Virchow Centre and the Nencki Institute of Experimental Biology on a 12:12 light: dark cycle and allowed free access to a regular chow diet and water. In all cases, experimental and control animals were of the same age and gender. Mouse experiments were approved by the district government of Lower Frankonia (Bezirksregierung Unterfranken, reference 55.2.2-2532-2-1296, 55.2-2532-2-999, 55.2-2532-2-435 and 55.2-2532-2-746) and the local ethic committee of Warsaw (I Lokalna Komisja Etyczna ds. Doświadczeń na Zwierzętach, reference: 1054/2020 and 1074/2020).

Generation of bone marrow chimeras

Five to seven week-old recipient C57BL/6JRj male mice were irradiated with 10 Gy in a radiation device (Faxitron). Subsequently, they were reconstituted by intravenous injection with 4×10^6 bone marrow cells from the femur and tibia of donor male mice (Gp1bα$^{-/-}$ or littermate Gp1bα$^{+/+}$ control; Gp6$^{-/-}$ or littermate Gp6$^{+/+}$ control). In the following 2 weeks, mice were provided with 2 mg/ml neomycin-sulfate (Sigma-Aldrich) supplemented drinking water. Six weeks after reconstitution mice were used for experiments.

Antibody application

Platelet depletion was induced in male and female mice by i.v. injection of 2 μg/g body weight of the polyclonal rat anti-mouse GPIbα antibodies R300 (Emfret Analytics) (Stegner *et al*, 2017). To functionally block the platelet receptors GPVI, GPIbα, and αIIbβ3 4 μg/g body weight of JAQ1-F(ab′)$_2$ (Nieswandt *et al*, 2001), p0p/B-Fab (Massberg *et al*, 2003) and JON/A-F(ab′)$_2$ (Bergmeier *et al*, 2002), respectively (all from Emfret Analytics) were injected i.v. to male mice. GPVI was depleted in male mice by i.v. injection of 4 μg/g body weight of JAQ1-IgG (Emfret Analytics) (Nieswandt *et al*, 2001). All control mice received non-immune rat IgG (Emfret Analytics) (Stegner *et al*, 2017).

Clopidogrel treatment

A solution of 150 or 375 mg/l of clopidogrel (Sanofi-Aventis) was given *ad libitum* by drinking water to mice (males and females) or rats (males), respectively, to ensure an assumed daily dosage of 40 mg per kg of body weight (Lieschke *et al*, 2020).

HET0016 treatment

Male mice received an i.p. injection of 10 mg HET0016 per kg of body weight (Sigma-Aldrich) (Wu *et al*, 2022) dissolved in DMSO (Roth) 72 h, 48 h, 24 h, and 30 min before the experiment. Control mice received DMSO.

In vivo metabolic tests

For the glucose tolerance test, male and female mice and male rats were fasted for 16 h. Unless otherwise stated all animals received an i.p. injection of 2 g of glucose per kg of body weight in (20% w/v glucose in saline). The insulin tolerance test was performed after 4 h (male and female mice) or 6 h (male rats) of fasting. This was followed by an i.p. injection of 0.5 U (mice) or 0.75 U (rats) of

© 2023 The Authors

insulin (Sanofi Aventis) per kg of body weight in saline solution (if not otherwise stated). To measure glucose-stimulated insulin secretion animals were fasted for 16 h followed by i.p. injection of 3 g (male and female mice) or 2 g (male rats) (Sunil *et al*, 2014) of glucose per kg of body weight (30% w/v glucose in saline). Blood samples were taken by tail vein puncture at indicated times. Blood glucose levels were measured by an automated glucometer (Accu-Chek, Roche) and serum insulin was measured by ELISA (Crystal Chem).

Platelet activation upon glucose injection

Male mice were fasted for 16 h and received an i.v. injection of glucose (2 g/kg in saline) or saline. After indicated timepoints, mice were bled into 300 µl heparin and platelet count was assessed for normalization. Finally, samples were centrifuged at 800 *g* at RT for 6 min and levels of PBP of the resulting supernatant were measured by ELISA (RayBiotech) as described in the manufacturer's manual.

Pancreas intravital imaging

The vasculature was visualized by injection of anti-CD105 Alexa Fluor 647 (clone MJ7/19, purified in-house, 0.4 µg per g of body weight), and platelets were visualized by injection of Alexa Fluor 594-labeled anti-GPIX derivative (Stegner *et al*, 2017) (0.6 µg per g of body weight). Male mice were anesthetized with 0.5 mg Medetomidine (Pfizer) per kg of body weight, 5 mg Midazolam (Roche) per kg of body weight, and 0.05 mg Fentanyl per kg of body weight (Janssen-Cilag GmbH), and access to the pancreas was generated by laparotomy of the upper abdominal below the costal arch. The intestine and stomach were carefully pushed aside with a cotton stick to expose a small part of the pancreas for further microscopy. To avoid drying out, the surrounding tissue was covered with a cloth soaked with physiological saline solution. Mice were placed on an inverted Leica SP8 confocal microscope, pancreatic islets were localized by morphology and vascular density, and imaging was performed using a 25× objective. Image stacks were processed, visualized, and analyzed using Fiji (Schindelin *et al*, 2012).

Image analysis

3D multicolor image stack files were preprocessed using Huygens Deconvolution software (Huygens Professional 20.10.0p1 64bit, Scientific Volume Imaging B.V., Hilversum, The Netherlands) to obtain higher contrast and better signal-to-noise ratio (SNR). Deconvolution was performed using the Classic Maximum Likelihood Estimation (CMLE) model, with a max. of 40 iterations, 10 SNR, and automatic background estimation. Results were converted to Imaris Classic format and segmented using Imaris (Imaris ×64, version 9.6, Bitplane AG, Zurich, Switzerland). Two segmentations were performed with each stack. First, the fluorescence intensity of CD105-stained cells served to determine blood vessel walls. Second, the fluorescence signal from GPIX-positive cells was used to identify platelets. Both findings were performed using the Surface Model tool in combination with shortest distance calculation, rolling ball background subtraction, and manual threshold adjustment. Vascular objects smaller than $< 100\ \mu m^3$ were filtered out. Adherent platelets were spread on the blood vessels and could be scanned in their full size. The rolling platelets appeared smaller in size due to volume underestimation (under sampling) resulting from the relatively low speed (compared to the blood circulation) of the microscope scanner. Hence, a size filter ($> 8\ \mu m^3$) was used to distinguish adherent platelets from motile ones. Furthermore, the manual removal of scan-related artifacts was necessary. Distance transformation was used to determine the blood platelets adhered to the vessel wall (distance = 0 µm). The volumes of these vessel walls and adherent platelets were exported, and the volume ratio was plotted as bar graphs. 3D images were generated using the "Snapshot" tool.

Generation of supernatant of activated platelets

To obtain mouse platelet-rich plasma (PRP) heparinized blood from males was centrifuged twice at 300 *g* for 6 min. PRP was supplemented with 0.02 U/ml apyrase (Sigma-Aldrich) and 0.1 µg/ml PGI_2 (Sigma-Aldrich) followed by pelleting the platelets at 800 *g* for 5 min by centrifugation. Platelets were washed twice by resuspending them in Tyrodes-HEPES buffer (134 mM NaCl (Roth), 12 mM $NaHCO_3$ (Roth), 5 mM HEPES (Roth), 2.9 mM KCl (Roth), 0.34 mM Na_2HPO_4 (Roth), 5 mM glucose (Roth), 0.35% w/v BSA (Sigma Aldrich), pH 7.4) containing 0.02 U/ml apyrase and 0.1 µg/ml PGI_2. The platelet count was adjusted to 500,000 platelets/µl in Tyrodes-HEPES buffer containing 2 mM $CaCl_2$ (Roth). Platelet activation was induced by 10 µg/ml collagen-related peptide (CRP, generated as previously described) (Knight *et al*, 1999). After 15 min of incubation, platelet debris were removed by 800 *g* centrifugation with subsequent sterile filtration. The generation of the control solution was identical except for the presence of platelets.

To obtain supernatant of activated human platelets blood was collected from healthy volunteers. Informed consent from blood donors was obtained in accordance with the ethical standards adhering to the local Institutional Review Boards and the Helsinki protocol. The ethics committee of the University of Würzburg Germany (reference: 167/17-sc) approved the study. PRP was prepared from 0.31 (w/v) citrate anticoagulated blood by centrifugation at 300 *g* for 20 min. Platelets were washed twice by resuspending them in Tyrodes-HEPES buffer containing 0.02 U/ml apyrase and 0.1 µg/ml PGI_2. The platelet count was adjusted to 1,000,000 platelets/µl in Tyrodes-HEPES buffer containing 2 mM $CaCl_2$ (Roth). Platelet activation was induced by 10 µg/ml collagen (Chrono-Log). After 15 min of incubation platelet debris were removed by 800 *g* centrifugation with subsequent sterile filtration. The generation of the control solution was identical except for the presence of platelets.

Modification of supernatant of activated platelets

To digest proteins and peptides supernatant of activated platelets and control solution were treated with 0.2 U/ml proteinase K (Sigma Aldrich) for 16 h at 37°C, followed by 1 h incubation with 0.2 mM proteinase K inhibitor (Calbiochem). Two fractions of substances smaller and bigger than 3 kDa were obtained using Amicon Ultra-0.5 ml centrifugal filters (Merck). Fraction bigger than 3 kDa was diluted in Tyrodes-HEPES buffer containing 2 mM $CaCl_2$ to obtain starting volume of the solution. To digest purines or to degrade histamine or serotonin supernatant of activated platelets

 © 2023 The Authors

and control solution were treated with 2×10^{-3} U/ml apyrase (Sigma Aldrich), 3×10^{-4} U/ml diamine oxidase (Sigma Aldrich), or 19 μg/ml monoamine oxidase A (Sigma Aldrich), respectively, for 16 h at 37°C. Treatment of monoamine oxidase A was followed by separation with a centrifugal filter unit and only a fraction of substances smaller than 3 kDa were used for further experiments. During the generation of modified solutions, additional vials of supernatant of activated platelets and control solution were subjected to the same treatment except for the presence of appropriate enzymes and inhibitors.

To deplete the supernatant of activated platelets from lipids solution was added to twice the volume of 2:1 v/v mixture of chloroform (Sigma Aldrich) and methanol (Poch), vortexed for 1 min. and centrifuged at 3,000 *g*, 4°C for 10 min. The upper, water phase was transferred to a new vial and the procedure was repeated two additional times. The final upper phase was used for further experiments.

Mouse islet isolation and GSIS

Pancreatic islets were isolated as previously described (Sumara *et al*, 2009). To measure insulin release, 8 size matching islets per 96-well were statically acclimated in 2.8 mM glucose Krebs-Ringer-Buffer (KRB, 135 mM NaCl, 20 mM HEPES, 5 mM KCl (Roth), 1 mM $MgSO_4$ (Roth), 1 mM $CaCl_2$, 0.4 mM K_2HPO_4 (Roth), 0.5% w/v BSA, pH 7.4) for 30 min. Afterward, islets were stimulated with a 1:1 mixture of KRB and supernatant of activated platelets or control solution supplemented with 0.5% w/v BSA and 2.8 mM or 16.7 mM glucose for 90 min. The supernatant was taken and insulin levels were measured by ELISA (Crystal Chem). Data were normalized to the basal secretion of control solution-treated islets.

Cell culture and GSIS

All cell lines were regularly tested for mycoplasma contamination.

MIN6 cells, a gift from Prof. R. Ricci (University of Strasbourg), were cultivated in Dulbecco's modified Eagle's medium (DMEM) (Thermo Scientific) supplemented with 15% v/v FBS, 20 mM HEPES (Sigma Aldrich), 50 μM β-mercaptoethanol (Sigma Aldrich), and 1% v/v PenStrep (Thermo Scientific). For insulin secretion assay, 30,000 cells/well were plated 48 h before on a MatriGel (Corning) coated 96-well plate. Before the experiment cells were kept for 2 h in 2.8 mM glucose KRB for acclimation. Afterward, cells were stimulated for 1 h with 2.8 mM glucose KRB followed by 1 h stimulation with a 1:1 mixture of KRB and supernatant of activated platelets or control solution containing 25 mM glucose and 0.5% w/v BSA. Supernatant from low and high-glucose-stimulated cells were taken and insulin levels were determined by ELISA (Crystal Chem).

INS-1 cells, a gift from prof. R. Ricci (University of Strasbourg), were cultivated in Roswell Park Memorial Institute (RPMI) 1640 medium (Thermo Scientific) supplemented with 10% v/v FBS, 10 mM HEPES, 1 mM sodium pyruvate, 2 mM glutamine, 50 μM β-mercaptoethanol and 1% v/v PenStrep. 100,000 cells/well were plated 48 h before the insulin secretion assay on a 48-well plate. After acclimation for 1 h in 2.8 mM glucose KRB, cells were stimulated for 30 min with a 1:1 mixture of KRB and supernatant of activated platelets or control solution, containing 2.8 mM or 15 mM glucose and 0.5% w/v BSA. Insulin levels of the medium were determined by ELISA (Crystal Chem). For co-culture of INS-1 cells with isolated human platelets after acclimation for 1 h in 2.8 mM glucose KRB, cells were stimulated for 30 min with a 1:1 mixture of KRB and suspension of 10^6/μl platelets in Tyrodes-HEPES buffer or control solution, containing 2.8 mM glucose and 0.5% w/v BSA. To inhibit Gαq/11, FFAR1, PKD1, and 20-HETE synthesis cells were treated with 5 nM YM-254890 (Tocris), 5 μM GW1100 (Cayman), 1 μM CRT0066101 (Tocris) and 30 μM HET0016 (Sigma-Aldrich), respectively, for 2 h prior to and during 30 min incubation with a 1:1 mixture of KRB and supernatant of activated platelets or control solution, containing 15 mM glucose and 0.5% w/v BSA. Insulin levels of the medium were determined by ELISA (Crystal Chem). The concentration of used drugs was chosen based on cytotoxicity analysis, the highest possible non-toxic dose was used for further experiments.

EndoC-βH1 cells were provided by Endocell and Raphael Scharfmann and cultivated as described previously (Hastoy *et al*, 2018). For the insulin secretion assay cells were seeded onto 24 well plates at a density of 300,000 cells/well. The night before the experiment, the cells were incubated in a 2.8 mM glucose culture medium. Prior to the experiment, the cells were incubated in a modified Krebs-Ringer buffer (modKRB) medium consisting of 138 mM NaCl, 3.6 mM KCl, 0.5 mM $MgSO_4$, 0.5 mM NaH_2PO_4, 5 mM $NaHCO_3$, 1.5 mM $CaCl_2$ and 5 mM HEPES and supplemented with 0.2% w/v BSA. The cells were preincubated for 15 min at 1 mM glucose before a 40 min test incubation in a 1:1 mixture of modKRB and either supernatant of activated platelets or control solution as indicated, supplemented with 0.2% w/v BSA and glucose (2.5 or 20 mM). Supernatants were taken for the determination of insulin release. Cellular insulin content was extracted by acid ethanol treatment. Insulin levels were determined by ELISA (Alpha Laboratories).

EndoC-βH5, non-proliferative mature human pancreatic cells, were provided by Human Cell Design. Cells were plated onto βCOAT (Human Cell Design) coated 96-well plates at a density of 100,000 cells/well and cultured in ULTIβ1 (Human Cell Design) medium for a week. Six days after seeding cells were fasted in ULTI-ST (Human Cell Design) for 24 h. Before the experiment, cells were preincubated in βKREBS (Human Cell Design) supplemented with 0.1% w/v BSA fraction V fatty acid-free (Sigma-Aldrich) for 1 h. Next, cells were incubated for 40 min in a 1:1 mixture of βKREBS and either supernatant of activated platelets or control solution as indicated, supplemented with 0.1% w/v BSA fraction V fatty acid-free and glucose (2.8 or 20 mM). Supernatants were collected for the determination of insulin release. Cellular insulin content was extracted by lysis with RIPA buffer (Sigma-Aldrich). Insulin levels were determined by ELISA (Demeditec Diagnostics).

HEK293 cells (ATCC) were cultivated in Dulbecco's modified Eagle's medium (DMEM) (Thermo Scientific) supplemented with 10% v/v FBS and 1% v/v PenStrep (Thermo Scientific).

3T3L1 cells (ATCC) were cultivated in Dulbecco's modified Eagle's medium (DMEM) (Thermo Scientific) supplemented with 10% v/v FCS and 0.4% v/v gentamicin (Thermo Scientific).

Reverse transfection with siRNA of INS1 cells

To silence FFAR1 expression in INS1 cells transfection with siRNA of cells in suspension was performed. siRNA and Dharmafect-Duo transfection reagent (Horizon) were diluted in Opti-MEM I medium

© 2023 The Authors

(Gibco) separately before being mixed and added to MatriGel-coated 48-well plates. Suspension of cells in an antibiotic-free medium was added on top of the preincubated siRNA-Dharmafect mix (100,000 cells/well). The final concentration of siRNA and Dharmafect was 0.1 μM and 7.7 μl/ml, respectively. The transfection was performed 48 h before GSIS experiments. siRNA sequences for FFAR1 were purchased as SMARTPool from Horizon. ON-TARGETplus Non-targeting Control Pool (Horizon) was used as a negative control.

Generation of cell supernatants

300,000 MIN6, HEK293, or 3T3L1 cells were plated on a MatriGel-coated 12-well plate for 48 h. Before stimulation cells were kept for 2 h in 2.8 mM glucose KRB for acclimation. For stimulation, 250 μl of 2.8 mM or 25 mM glucose KRB was added for 3 min. The supernatant was collected and centrifuged at 300 *g* for 5 min. Control supernatant was generated similarly, namely, KRB was incubated with Matrigel-coated 12-well plates. The supernatant was transferred to a new tube and centrifuged at 14,000 *g* for 10 min. Afterward, the supernatant was collected and stored at −80°C. For the generation of control supernatant, an identical procedure was applied without the use of cells.

Flow cytometry

Heparinized whole blood (20 U/ml, Ratiopharm) was washed twice with Tyrode-HEPES buffer and diluted (1:20) in Tyrode-HEPES buffer containing 2 mM $CaCl_2$. Blood was incubated in the presence of indicated agonist with saturating amounts of fluorophore-conjugated antibodies against αIIbβ3 (JON/A-PE, 1/7, Emfret Analytics) and P-selectin (WUG.E9-FITC, 1/7, Emfret Analytics) for 6 min at 37°C followed by 6 min at room temperature. In the case of the stimulation experiment with different cell supernatants, the supernatants were adjusted to 25 mM glucose and added in a ratio of 1:3 in the presence of 10 μg/ml CRP. Stimulation was stopped by the addition of 500 μl PBS and samples were quantified by FACSCelesta (BD Biosciences).

Platelet adhesion under flow conditions

To assess the impact of glucose on platelet adhesion 800 μl heparinized blood derived from human donors or male mice was mixed with 400 μl Tyrode-HEPES buffer and supplemented with glucose to obtain a concentration of 2.8 or 25 mM glucose. For experiments with cell supernatants, 200 μl heparinized mouse blood was mixed with 100 μl cell supernatant and 100 μl Tyrode-HEPES buffer supplemented with glucose to obtain a concentration of 25 mM. Platelets were fluorescently labeled for 5 min at 37°C using a Dylight488-labeled anti-GPIX antibody derivative (0.1 μg/ml, for mouse platelets) and a Dylight488-labeled anti-GPIbβ (0.1 μg/ml, for human platelets), respectively.

Coverslips (24 × 60 mm) were coated with 200 μg/ml collagen I in SFK buffer (Takeda) overnight at 37°C and blocked for 30 min with 1% w/v BSA in PBS at room temperature. The coated coverslip was inserted into a transparent flow chamber with a slit depth of 50 μm and rinsed with Tyrodes-HEPES buffer supplemented with 2 mM $CaCl_2$ and an equal glucose concentration as the blood sample. Perfusion of the blood sample occurred at room temperature using a pulse-free pump with a shear rate of 150/s for 8 min. This was followed by rinsing the chamber for 8 min at the same shear rate with Tyrodes-HEPES buffer with 2 mM $CaCl_2$ and sample matching glucose concentration. Microscope phase-contrast images were recorded in a real timer during the perfusion and rinsing process using a Zeiss Axiovert 200 inverted microscope (40×/0.60 objective) equipped with a CoolSNAP-EZ camera (Visitron). After rinsing, phase contrast and fluorescence images were recorded from at least 6 random microscope fields. Image quantification was done with Fiji (Schindelin *et al*, 2012) to obtain relative coverage of platelets as well as the integrated density of their fluorescence signal.

Calcium influx assay

INS1 cells were seeded on 24-well black plates with a clear bottom (Ibidi) 48 h prior to the experiment. To monitor the concentration of cytosolic calcium levels, cells were loaded with 2 μM fura-2/AM (Invitrogen) for 1 h in 2.8 mM glucose KRB. Afterward, cells were washed twice with PBS and cultured in 2.8 mM glucose KRB. After a few minutes of initial measurements of the basal level of cytosolic calcium, cells were stimulated with a 1:1 mixture of KRB and supernatant of activated platelets or a control solution, containing 15 mM glucose and 0.5% w/v BSA. Changes in the concentration of cytosolic calcium were measured using a microplate reader at Ex/Em 340/510 and 380/510 nm.

20-HETE quantification

For serum generation, mouse blood was kept at room temperature for 30 min followed by centrifugation at 2,000 *g* for 10 min to obtain serum. Human serum samples were generated and provided by in. vent Diagnostica in accordance with the ethical standards. 20-HETE concentrations were measured by ELISA (Abcam).

Immunohistochemical analysis

For all the immunohistochemical analyses perfusion of the male mice was performed before excision of the pancreas. Tissues were frozen in OCT on the dry ice. Cryosections with a thickness of 7 μM were fixed with 1% w/v para-formaldehyde lysine periodat (1% w/v para-formaldehyde, 28 mM NaH_2PO_4 (Roth), 9.4 mM Na_2HPO_4 (Roth), 0.9 M lysine (Roth), 12 mM $NaIO_4$ (Roth)) for 30 min at room temperature. After three times washing with PBS sections were blocked for 1 h at room temperature with blocking buffer (PBS, 5% v/v goat serum (Thermo Sciences)), 1% w/v cold water fish skin gelatin (Sigma Aldrich), 10 μg/ml Fc-Block (BD Biosciences). Primary rabbit anti-PECAM-1 antibody (Abcam) in blocking buffer (1/150) was added overnight at 4°C. After washing twice with washing buffer (PBS, 0.05% v/v Tween-20 (Roth)) and PBS, secondary rat-anti-rabbit-Alexa488 antibody (1/500, Thermo Sciences) and anti-GPIX-Alexa594 derivative (10 μg/ml, Stegner *et al*, 2017) were added and incubated for 1 h at room temperature. Sections were washed twice with washing buffer and PBS and covered with a coverslip using DAPI-mountant (Thermo Scientific). Fluorescence images of the exocrine and endocrine pancreas were recorded with a TCS SP8 confocal microscope (Leica Microsystems). For image analysis, background fluorescence was subtracted and

© 2023 The Authors

endothelial area, islet area, as well as platelet count, were determined by Fiji (Schindelin *et al*, 2012).

Islet size and pancreas insulin content measurements

For the determination of islet size paraffin and cryosections were used. Three different sections with a distance of 50 μm were taken per subject. For paraffin sections, islets were localized by standard hematoxylin–eosin staining, and pictures were taken in a Leica light microscope DM4000B. For cryosections, islets were visualized by prior described immunostaining using primary guinea pig anti-insulin antibody (1/180, Abcam) and secondary goat anti-guinea pig-Alexa-594 antibody (1/200, Thermo Scientific).

For insulin extraction pancreas was homogenized (Polytron) in 70% v/v ethanol (Roth) containing 1.5% v/v HCl (Sigma Aldrich) and kept at −20°C for 24 h. The homogenate was centrifuged and the supernatant was used to measure insulin content via ELISA (Crystal Chem). For normalization to protein, the content supernatant was neutralized with an equal amount of 1 M TRIS (pH 7.5, Sigma Aldrich), and protein content was determined by bicinchoninic acid assay (Thermo Scientific).

Western blot

Cells were lysed by a RIPA buffer (Thermo Scientific) supplemented with protease and phosphatase inhibitors mix (Thermo Scientific). BCA kit (Thermo Scientific) was used to determine the protein concentration of the extracts. Protein samples were loaded on 10% polyacrylamide gel, separated by electrophoresis, and transferred onto PVDF membranes (Merck). Membranes were blotted using appropriate antibodies. The signals were detected on autoradiography film with enhanced chemiluminescence solution (Bio-Rad). The following antibodies were used: anti-GAPDH (1/40,000, Sigma-Aldrich), secondary anti-rabbit (1/10,000, AMDEX), antibodies from Cell Signaling: anti-phospho-PKD Ser744/748 (1/1,000), anti-phospho-PKD Ser916 (1/1,000) and anti-phospho-(Ser/Thr) PKD substrate (1/1,000), pAkt Thr308 (1/1,000), Akt (1/1,000) and antibodies from Abcam: GLUT1 (1/1,000), GLUT3 (1/1,000), GLUT4 (1/1,000).

Real-time PCR analysis

RNA was isolated from cells using the Total RNA Mini kit (A&A Biotechnology) according to the manufacturer's protocol. cDNA was synthesized using 1 μg of RNA and a first-strand cDNA synthesis kit (Thermo-Scientific). Quantitative PCR was performed using 5 ng of cDNA, SYBR Green (Bio-Rad), and the respective pair of primer sequences. The level of FFAR1 was normalized to HPRT1 using the ΔΔCt method. Used genes and sequences were as follows: *Ffar1* (forward: 5′-CCCTTGGTTATCACTGCTTTCTG-3′; reverse: 5′-GAG CCTTCTAAGTCCGGGTTTAT-3′) and *Hprt1* (forward: 5′-GCAG ACTTTGCTTTCCTTGG-3′; reverse: 5′-CCGCTGTCTTTTAGGCTTTG -3′).

LC/MS Lipidomic analysis

Lipid analysis was performed as described in detail before (Bohnert *et al*, 2021) with minor modifications. Briefly, 10 μl samples were mixed with 170 μl 10 mM HCl, 190 μl methanol, and 20 μl external standards (100 μM D7-cholesterol, 50 μM D7-7DHC, 10 μM each of D31-hexadecanoic acid, LPA-(17:0) and LPC-(17:0), Merck, Darmstadt, Germany) in chloroform/methanol (1/1, v/v). Successively, 90 and 100 μl of chloroform were added with thorough mixing in between. The resulting upper phase was re-extracted with 300 μl synthetical lower phase. The combined lower phases were evaporated under a stream of nitrogen gas at 30°C. For further analysis, the resulting residues were dissolved in 50 μl 2-propanol. Three microliter samples were applicated to an Acclaim 120 C8 HPLC column (3 μm particles, 100 × 2.1 mm, Thermo Scientific). The LC separation (solvent A: MeOH/H2O/FA (5/94.9/0.1, v/v/v); solvent B: CH3CN/iPrOH/H2O/FA (45/45/9.9/0.1, v/v/v)) was performed at 45°C and a flow rate of 200 μl/min starting with 20% solvent B for 2 min followed by a linear increase to 100% solvent B within 7 min and maintaining it for 28 min, then returning to 20% solvent B within 1 min and keeping it for 5 min for equilibration before each sample injection. The eluent was directed to the HESI source of the Q Exactive mass spectrometer (Thermo Scientific) from 4 to 34 min after sample injection. Usage of a high-resolution orbitrap instrument allowed analysis of ions with a sub-ppm accuracy enabling a semi-targeted, semi-quantitative method. Chromatograms were recorded in alternating negative and positive mode at 70 k resolution with a scan range of 200–1,650 *m/z*. The peak areas originating from isomers differing in the position of double bonds were integrated as a whole. Peaks corresponding to the calculated monoisotopic metabolite masses (±3 mMU) were integrated using TraceFinder V3.3 software (Thermo Scientific). Retention time and *m/z* values of identified lipids are described in Table EV1.

Statistics

Results are presented as mean values ± standard error of the mean. For $n \geq 7$ data distribution was checked by the Shapiro–Wilk normality test. Unpaired *t*-test and one-way ANOVA followed by Sidak's multiple comparisons test were used for the analysis of the significance of data with the normal distribution of two groups and more than two groups, respectively. Whereas Mann–Whitney test and Kruskal–Wallis test followed by Mann–Whitney test as *post hoc* analysis with Benjamini–Hochberg correction for multiple comparisons were used for the analysis of the significance of data with not normal distribution of two groups and more than two groups, respectively. *P*-values lower than 0.05 were considered statistically significant. Sample sizes were defined by *a priori* power calculation with G-Power 3.1.9.4 software, considering a statistical power of 80% and $\alpha = 0.05$. Animals were assigned to the groups randomly. No blinding was assessed. No animal or sample was excluded from the analysis.

Human samples

For the generation of supernatant of activated human platelets, blood was collected from healthy volunteers of Caucasian race, both genders, in age 20–30 years old. Serum samples from healthy men in age 30–45 and 65–79 for 20-HETE analysis were purchased from invent Diagnostica GmbH. Experiments conformed to the principles set out in the WMA Declaration of Helsinki and the Department of Health and Human Services Belmont Report.

© 2023 The Authors

Study approval

All animal experiments were approved by the district government of Lower Frankonia (Bezirksregierung Unterfranken, reference 55.2.2-2532-2-1296, 55.2-2532-2-999, 55.2-2532-2-435 and 55.2-2532-2-746) and the local ethic committee of Warsaw (I Lokalna Komisja Etyczna ds. Doświadczeń na Zwierzętach, reference: 1054/2020 and 1074/2020). Experiments with donated human blood were approved by the ethics committee of the University of Würzburg Germany (reference: 167/17-sc). Informed consent from blood donors was obtained in accordance with the ethical standards adhering to the local Institutional Review Boards and the Helsinki protocol.

The paper explained

Problem

Diabetic patients demonstrate increased platelet activity. However, the mechanism underlying platelet hyperactivity during hyperglycemia is not fully explored. Moreover, the fact that elevated glucose levels impact platelet action indicates a potential link between platelet function and glucose metabolism.

Results

We showed that platelets' activity is directly regulated by glucose and pancreatic β cell-derived factors. We also demonstrated that a fraction of platelets localizes to the vasculature of pancreatic islets. Moreover, our findings indicate that platelets secrete lipid-based factors including 20-Hydroxyeicosatetraenoic acid (20-HETE) to stimulate insulin secretion. Finally, we demonstrated that platelets' impact on β cells declines with age.

Impact

For the first time, we demonstrated a cross-talk between platelets and pancreatic β cells therefore our findings open new areas of research. Our results indicate also new mechanisms and a group of substances that might be targeted to improve pancreatic β cell function.

Data availability

This study includes no data deposited in external repositories.

Expanded View for this article is available online.

Acknowledgements

We thank Dr. Stefan Offermanns from the Department of Pharmacology, Max-Planck-Institute for Heart and Lung Research, Ludwigstrasse 43, 61231 Bad Nauheim, Germany, for providing us with $G\alpha q^{fl/fl}$ and $G\alpha 13^{fl/fl}$ mice. We thank Dr. Olga Sumara and Dr. Izabela Sumara for their comments on the manuscript. This study was funded by Deutsche Forschungsgemeinschaft (DFG) project number 374031971–CRC/TR 240 project B10 (to GS), project A01, A07, B07 (to BN), project B06 (to DS and KGH) and project B08 (to DS); European Research Council (ERC) Starting Grant SicMetabol number 678119 (to TK, MCL, AL-V, JT-V, RE-M, and GS); Emmy Noether Grant Su 820/1-1 from Deutsche Forschungsgemeinschaft (DFG) (to JT-V, RE-M, and GS); European Molecular Biology Organization (EMBO) Installation Grant project number 4425 (2020-2024) (to KK, AD, and GS); Sonata bis grant 2020/38/E/NZ4/00314 from National Science Centre in Poland (Narodowe Centrum Nauki) (AB and GS); Diabetes UK RD Lawrence Fellowship (to BH), and the Dioscuri Centre of Scientific Excellence Grant number UMO-2018/01/H/NZ4/00002 (to KK, MW, FD, AD, AL-V, and GS) – the program initiated by the Max Planck Society (Max-Planc-Gesellschaft), managed jointly with the National Science Centre in Poland (Narodowe Centrum Nauki) and mutually funded by the Polish Ministry of Science and Higher Education (Ministerstwo Nauki i Szkolnictwa Wyższego) and the German Federal Ministry of Education and Research (Bundesministerium für Bildung und Forschung).

Author contributions

Till Karwen: Conceptualization; data curation; formal analysis; validation; investigation; visualization; methodology; writing – original draft; writing – review and editing. **Katarzyna Kolczynska-Matysiak:** Conceptualization; data curation; formal analysis; validation; investigation; visualization; methodology; writing – original draft; writing – review and editing. **Carina Gross:** Formal analysis; investigation; methodology. **Mona C Löffler:** Formal analysis; investigation; methodology. **Mike Friedrich:** Formal analysis; investigation; methodology. **Angel Loza-Valdes:** Formal analysis; investigation. **Werner Schmitz:** Formal analysis; investigation; methodology. **Magdalena Wit:** Investigation; visualization; methodology. **Filip Dziaczkowski:** Investigation; visualization. **Andrei Belykh:** Investigation; visualization. **Jonathan Trujillo-Viera:** Investigation. **Rabih El-Merahbi:** Investigation. **Carsten Deppermann:** Investigation; methodology. **Sameena Nawaz:** Methodology. **Benoit Hastoy:** Investigation; methodology. **Agnieszka Demczuk:** Investigation. **Manuela Erk:** Investigation. **Mariusz R Wieckowski:** Methodology. **Patrik Rorsman:** Conceptualization. **Katrin G Heinze:** Data curation; methodology. **David Stegner:** Conceptualization; data curation; formal analysis; supervision; validation; investigation; methodology; project administration; writing – review and editing. **Bernhard Nieswandt:** Conceptualization; data curation; formal analysis; supervision; validation; investigation; methodology; project administration; writing – review and editing. **Grzegorz Sumara:** Conceptualization; resources; data curation; formal analysis; supervision; funding acquisition; investigation; writing – original draft; project administration; writing – review and editing.

Disclosure and competing interests statement

The authors declare that they have no conflict of interest.

References

Ashcroft FM, Rorsman P (2012) Diabetes mellitus and the β cell: the last ten years. *Cell* 148: 1160 – 1171

Baht GS, Silkstone D, Vi L, Nadesan P, Amani Y, Whetstone H, Wei Q, Alman BA (2015) Exposure to a youthful circulation rejuvenates bone repair through modulation of β-catenin. *Nat Commun* 6: 1 – 10

Bender M, May F, Lorenz V, Thielmann I, Hagedorn I, Finney BA, Vögtle T, Remer K, Braun A, Bösl M (2013) Combined *in vivo* depletion of glycoprotein VI and C-type lectin-like receptor 2 severely compromises hemostasis and abrogates arterial thrombosis in mice. *Arterioscler Thromb Vasc Biol* 33: 926 – 934

Bergmeier W, Rackebrandt K, Schröder W, Zirngibl H, Nieswandt B (2000) Structural and functional characterization of the mouse von Willebrand factor receptor GPIb-IX with novel monoclonal antibodies. *Blood* 95: 886 – 893

 © 2023 The Authors

Bergmeier W, Schulte V, Brockhoff G, Bier U, Zirngibl H, Nieswandt B (2002) Flow cytometric detection of activated mouse integrin αIIbβ3 with a novel monoclonal antibody. *Cytometry* 48: 80 – 86

Bergmeier W, Piffath CL, Goerge T, Cifuni SM, Ruggeri ZM, Ware J, Wagner DD (2006) The role of platelet adhesion receptor GPIbα far exceeds that of its main ligand, von Willebrand factor, in arterial thrombosis. *Proc Natl Acad Sci USA* 103: 16900 – 16905

Bohnert S, Wirth C, Schmitz W, Trella S, Monoranu C-M, Ondruschka B, Bohnert M (2021) Myelin basic protein and neurofilament H in postmortem cerebrospinal fluid as surrogate markers of fatal traumatic brain injury. *Int J Leg Med* 135: 1525 – 1535

Burkard P, Vögtle T, Nieswandt B (2020) Platelets in thrombo-inflammation: concepts, mechanisms, and therapeutic strategies for ischemic stroke. *Hamostaseologie* 40: 153 – 164

Burns SM, Vetere A, Walpita D, Dančík V, Khodier C, Perez J, Clemons PA, Wagner BK, Altshuler D (2015) High-throughput luminescent reporter of insulin secretion for discovering regulators of pancreatic beta-cell function. *Cell Metab* 21: 126 – 137

Conboy IM, Conboy MJ, Wagers AJ, Girma ER, Weissman IL, Rando TA (2005) Rejuvenation of aged progenitor cells by exposure to a young systemic environment. *Nature* 433: 760 – 764

Deppermann C (2018) Platelets and vascular integrity. *Platelets* 29: 549 – 555

Duvernay MT, Matafonov A, Lindsley CW, Hamm HE (2015) Platelet lipidomic profiling: novel insight into cytosolic phospholipase A2α activity and its role in human platelet activation. *Biochemistry* 54: 5578 – 5588

El-Merahbi R, Löffler M, Mayer A, Sumara G (2015) The roles of peripheral serotonin in metabolic homeostasis. *FEBS Lett* 589: 1728 – 1734

El-Merahbi R, Viera JT, Valdes AL, Kolczynska K, Reuter S, Löffler MC, Erk M, Ade CP, Karwen T, Mayer AE (2020) The adrenergic-induced ERK3 pathway drives lipolysis and suppresses energy dissipation. *Genes Dev* 34: 495 – 510

Ferdaoussi M, Bergeron V, Zarrouki B, Kolic J, Cantley J, Fielitz J, Olson E, Prentki M, Biden T, MacDonald P (2012) G protein-coupled receptor (GPR) 40-dependent potentiation of insulin secretion in mouse islets is mediated by protein kinase D1. *Diabetologia* 55: 2682 – 2692

Ferreira IA, Eybrechts KL, Mocking AI, Kroner C, Akkerman J-WN (2004) IRS-1 mediates inhibition of Ca^{2+} mobilization by insulin via the inhibitory G-protein Gi. *J Biol Chem* 279: 3254 – 3264

Fidler TP, Marti A, Gerth K, Middleton EA, Campbell RA, Rondina MT, Weyrich AS, Abel ED (2019) Glucose metabolism is required for platelet hyperactivation in a murine model of type 1 diabetes. *Diabetes* 68: 932 – 938

Fotino C, Dal Ben D, Adinolfi E (2018) Emerging roles of purinergic signaling in diabetes. *Med Chem* 14: 428 – 438

Fujita H, Hashimoto Y, Russell S, Zieger B, Ware J (1998) *In vivo* expression of murine platelet glycoprotein Ibα. *Blood* 92: 488 – 495

Hall E, Jönsson J, Ofori JK, Volkov P, Perfilyev A, Dekker Nitert M, Eliasson L, Ling C, Bacos K (2019) Glucolipotoxicity alters insulin secretion via epigenetic changes in human islets. *Diabetes* 68: 1965 – 1974

Hastoy B, Godazgar M, Clark A, Nylander V, Spiliotis I, van de Bunt M, Chibalina MV, Barrett A, Burrows C, Tarasov AI *et al* (2018) Electrophysiological properties of human beta-cell lines EndoC-betaH1 and -betaH2 conform with human beta-cells. *Sci Rep* 8: 16994

Helman A, Klochendler A, Azazmeh N, Gabai Y, Horwitz E, Anzi S, Swisa A, Condiotti R, Granit RZ, Nevo Y (2016) p16Ink4a-induced senescence of pancreatic beta cells enhances insulin secretion. *Nat Med* 22: 412 – 420

Hogan MF, Hull RL (2017) The islet endothelial cell: a novel contributor to beta cell secretory dysfunction in diabetes. *Diabetologia* 60: 952 – 959

Holinstat M (2017) Normal platelet function. *Cancer Metastasis Rev* 36: 195 – 198

Hopkins BD, Goncalves MD, Cantley LC (2020) Insulin–PI3K signalling: an evolutionarily insulated metabolic driver of cancer. *Nat Rev Endocrinol* 16: 276 – 283

Ho-Tin-Noé B, Boulaftali Y, Camerer E (2018) Platelets and vascular integrity: how platelets prevent bleeding in inflammation. *Blood* 131: 277 – 288

Hu H, Hjemdahl P, Li N (2002) Effects of insulin on platelet and leukocyte activity in whole blood. *Thromb Res* 107: 209 – 215

Huang G, Ge G, Wang D, Gopalakrishnan B, Butz DH, Colman RJ, Nagy A, Greenspan DS (2011) α3 (V) collagen is critical for glucose homeostasis in mice due to effects in pancreatic islets and peripheral tissues. *J Clin Invest* 121: 769 – 783

Jain K, Tyagi T, Patell K, Xie Y, Kadado AJ, Lee SH, Yarovinsky T, Du J, Hwang J, Martin KA (2019) Age associated non-linear regulation of redox homeostasis in the anucleate platelet: implications for CVD risk patients. *EBioMedicine* 44: 28 – 40

Jonsson A, Hedin A, Müller M, Skog O, Korsgren O (2020) Transcriptional profiles of human islet and exocrine endothelial cells in subjects with or without impaired glucose metabolism. *Sci Rep* 10: 22315

Kanaji T, Russell S, Ware J (2002) Amelioration of the macrothrombocytopenia associated with the murine Bernard-Soulier syndrome. *Blood* 100: 2102 – 2107

Katsimpardi L, Litterman NK, Schein PA, Miller CM, Loffredo FS, Wojtkiewicz GR, Chen JW, Lee RT, Wagers AJ, Rubin LL (2014) Vascular and neurogenic rejuvenation of the aging mouse brain by young systemic factors. *Science* 344: 630 – 634

Knight CG, Morton LF, Onley DJ, Peachey AR, Ichinohe T, Okuma M, Farndale RW, Barnes MJ (1999) Collagen-platelet interaction: Gly-Pro-Hyp is uniquely specific for platelet Gp VI and mediates platelet activation by collagen. *Cardiovasc Res* 41: 450 – 457

Kolczynska K, Loza-Valdes A, Hawro I, Sumara G (2020) Diacylglycerol-evoked activation of PKC and PKD isoforms in regulation of glucose and lipid metabolism: a review. *Lipids Health Dis* 19: 1 – 15

Kristinsson H, Smith DM, Bergsten P, Sargsyan E (2013) FFAR1 is involved in both the acute and chronic effects of palmitate on insulin secretion. *Endocrinology* 154: 4078 – 4088

Laakso M, Lehto S (1998) Epidemiology of risk factors for cardiovascular disease in diabetes and impaired glucose tolerance. *Atherosclerosis* 137: S65 – S73

Laychock SG (1985) Effects of hydroxyeicosatetraenoic acids on fatty acid esterification in phospholipids and insulin secretion in pancreatic islets. *Endocrinology* 117: 1011 – 1019

Levoux J, Prola A, Lafuste P, Gervais M, Chevallier N, Koumaiha Z, Kefi K, Braud L, Schmitt A, Yacia A (2021) Platelets facilitate the wound-healing capability of mesenchymal stem cells by mitochondrial transfer and metabolic reprogramming. *Cell Metab* 33: e289

Lieschke F, Zheng Y, Schaefer JH, Van Leyen K, Foerch C (2020) Measurement of platelet function in an experimental stroke model with aspirin and clopidogrel treatment. *Front Neurol* 11: 85

Löffler MC, Mayer AE, Trujillo Viera J, Loza Valdes A, El-Merahbi R, Ade CP, Karwen T, Schmitz W, Slotta A, Erk M (2018) Protein kinase D1 deletion in adipocytes enhances energy dissipation and protects against adiposity. *EMBO J* 37: e99182

Loza-Valdes A, Mayer AE, Kassouf T, Trujillo-Viera J, Schmitz W, Dziaczkowski F, Leitges M, Schlosser A, Sumara G (2021) A phosphoproteomic approach reveals that PKD3 controls PKA-mediated glucose and tyrosine metabolism. *Life Sci Alliance* 4: e202000863

Mahmoodian R, Salimian M, Hamidpour M, Khadem-Maboudi AA, Gharehbaghian A (2019) The effect of mild agonist stimulation on the

platelet reactivity in patients with type 2 diabetes mellitus. *BMC Endocr Disord* 19: 62

Malachowska B, Tomasik B, Szadkowska A, Baranowska-Jazwiecka A, Wegner O, Mlynarski W, Fendler W (2015) Altered platelets' morphological parameters in children with type 1 diabetes–a case-control study. *BMC Endocr Disord* 15: 1 – 7

Malehmir M, Pfister D, Gallage S, Szydlowska M, Inverso D, Kotsiliti E, Leone V, Peiseler M, Surewaard BG, Rath D (2019) Platelet GPIbα is a mediator and potential interventional target for NASH and subsequent liver cancer. *Nat Med* 25: 641 – 655

Mancuso ME, Santagostino E (2017) Platelets: much more than bricks in a breached wall. *Br J Haematol* 178: 209 – 219

Manne BK, Xiang SC, Rondina MT (2017) Platelet secretion in inflammatory and infectious diseases. *Platelets* 28: 155 – 164

Massberg S, Gawaz M, Grüner S, Schulte V, Konrad I, Zohlnhöfer D, Heinzmann U, Nieswandt B (2003) A crucial role of glycoprotein VI for platelet recruitment to the injured arterial wall *in vivo*. *J Exp Med* 197: 41 – 49

Mayer AE, Löffler MC, Loza Valdés AE, Schmitz W, El-Merahbi R, Viera JT, Erk M, Zhang T, Braun U, Heikenwalder M (2019) The kinase PKD3 provides negative feedback on cholesterol and triglyceride synthesis by suppressing insulin signaling. *Sci Signal* 12: eaav9150

McKillop A, Moran B, Abdel-Wahab Y, Flatt P (2013) Evaluation of the insulin releasing and antihyperglycaemic activities of GPR55 lipid agonists using clonal beta-cells, isolated pancreatic islets and mice. *Br J Pharmacol* 170: 978 – 990

Moers A, Nieswandt B, Massberg S, Wettschureck N, Gruner S, Konrad I, Schulte V, Aktas B, Gratacap MP, Simon MI *et al* (2003) G13 is an essential mediator of platelet activation in hemostasis and thrombosis. *Nat Med* 9: 1418 – 1422

Moers A, Wettschureck N, Gruner S, Nieswandt B, Offermanns S (2004) Unresponsiveness of platelets lacking both $G\alpha_q$ and $G\alpha_{13}$. Implications for collagen-induced platelet activation. *J Biol Chem* 279: 45354 – 45359

Moran B, Abdel-Wahab Y, Flatt P, McKillop A (2014) Evaluation of the insulin-releasing and glucose-lowering effects of GPR120 activation in pancreatic β-cells. *Diabetes Obes Metab* 16: 1128 – 1139

Mylotte D, Kavanagh GF, Peace AJ, Tedesco AF, Carmody D, O'Reilly M, Foley DP, Thompson CJ, Agha A, Smith D *et al* (2012) Platelet reactivity in type 2 diabetes mellitus: a comparative analysis with survivors of myocardial infarction and the role of glycaemic control. *Platelets* 23: 439 – 446

Nakamura T, Yoshikawa T, Noguchi N, Sugawara A, Kasajima A, Sasano H, Yanai K (2014) The expression and function of histamine H3 receptors in pancreatic beta cells. *Br J Pharmacol* 171: 171 – 185

Nieswandt B, Schulte V, Bergmeier W, Mokhtari-Nejad R, Rackebrandt K, Cazenave J-P, Ohlmann P, Gachet C, Zirngibl H (2001) Long-term antithrombotic protection by *in vivo* depletion of platelet glycoprotein VI in mice. *J Exp Med* 193: 459 – 470

Peng B, Geue S, Coman C, Münzer P, Kopczynski D, Has C, Hoffmann N, Manke M-C, Lang F, Sickmann A (2018) Identification of key lipids critical for platelet activation by comprehensive analysis of the platelet lipidome. *Blood* 132: e1 – e12

Ravassard P, Hazhouz Y, Pechberty S, Bricout-Neveu E, Armanet M, Czernichow P, Scharfmann R (2011) A genetically engineered human pancreatic β cell line exhibiting glucose-inducible insulin secretion. *J Clin Invest* 121: 3589 – 3597

Richards-Williams C, Contreras JL, Berecek KH, Schwiebert EM (2008) Extracellular ATP and zinc are co-secreted with insulin and activate multiple P2X purinergic receptor channels expressed by islet beta-cells to potentiate insulin secretion. *Purinergic Signal* 4: 393 – 405

Rorsman P, Braun M (2013) Regulation of insulin secretion in human pancreatic islets. *Annu Rev Physiol* 75: 155 – 179

Savi P, Herbert J-M (2005) Clopidogrel and ticlopidine: P2Y12 adenosine diphosphate-receptor antagonists for the prevention of atherothrombosis. Seminars in thrombosis and hemostasis. Thieme Medical Publishers, Inc., New York, NY, pp. 174–183

Schindelin J, Arganda-Carreras I, Frise E, Kaynig V, Longair M, Pietzsch T, Preibisch S, Rueden C, Saalfeld S, Schmid B *et al* (2012) Fiji: an open-source platform for biological-image analysis. *Nat Methods* 9: 676 – 682

Schnell S, Schaefer M, Schöfl C (2007) Free fatty acids increase cytosolic free calcium and stimulate insulin secretion from β-cells through activation of GPR40. *Mol Cell Endocrinol* 263: 173 – 180

Siegel-Axel DI, Gawaz M (2007) Platelets and endothelial cells. Seminars in thrombosis and hemostasis. Thieme Medical Publishers, Inc., New York, NY, pp. 128–135

Stegner D, vanEeuwijk JMM, Angay O, Gorelashvili MG, Semeniak D, Pinnecker J, Schmithausen P, Meyer I, Friedrich M, Dutting S *et al* (2017) Thrombopoiesis is spatially regulated by the bone marrow vasculature. *Nat Commun* 8: 127

Stegner D, Göb V, Krenzlin V, Beck S, Hemmen K, Schuhmann MK, Schörg BF, Hackenbroch C, May F, Burkard P (2022) Foudroyant cerebral venous (sinus) thrombosis triggered through CLEC-2 and GPIIb/IIIa dependent platelet activation. *Nat Cardiovasc Res* 1: 132 – 141

Stritt S, Beck S, Becker IC, Vögtle T, Hakala M, Heinze KG, Du X, Bender M, Braun A, Lappalainen P (2017) Twinfilin 2a regulates platelet reactivity and turnover in mice. *Blood* 130: 1746 – 1756

Sudic D, Razmara M, Forslund M, Ji Q, Hjemdahl P, Li N (2006) High glucose levels enhance platelet activation: involvement of multiple mechanisms. *Br J Haematol* 133: 315 – 322

Sumara G, Formentini I, Collins S, Sumara I, Windak R, Bodenmiller B, Ramracheya R, Caille D, Jiang H, Platt KA (2009) Regulation of PKD by the MAPK p38δ in insulin secretion and glucose homeostasis. *Cell* 136: 235 – 248

Sumara G, Sumara O, Kim JK, Karsenty G (2012) Gut-derived serotonin is a multifunctional determinant to fasting adaptation. *Cell Metab* 16: 588 – 600

Sunil V, Verma MK, Oommen AM, Sadasivuni M, Singh J, Vijayraghav DN, Chandravanshi B, Shetty J, Biswas S, Dandu A (2014) CNX-011-67, a novel GPR40 agonist, enhances glucose responsiveness, insulin secretion and islet insulin content in n-STZ rats and in islets from type 2 diabetic patients. *BMC Pharmacol Toxicol* 15: 1 – 15

Szczerbinska I, Tessitore A, Hansson LK, Agrawal A, Ragel Lopez A, Helenius M, Malinowski AR, Gilboa B, Ruby MA, Gupta R (2022) Large-scale functional genomics screen to identify modulators of human β-cell insulin secretion. *Biomedicine* 10: 103

Tang WH, Stitham J, Gleim S, Di Febbo C, Porreca E, Fava C, Tacconelli S, Capone M, Evangelista V, Levantesi G *et al* (2011) Glucose and collagen regulate human platelet activity through aldose reductase induction of thromboxane. *J Clin Invest* 121: 4462 – 4476

Tiedt R, Schomber T, Hao-Shen H, Skoda RC (2007) Pf4-Cre transgenic mice allow the generation of lineage-restricted gene knockouts for studying megakaryocyte and platelet function *in vivo*. *Blood* 109: 1503 – 1506

Trujillo-Viera J, El-Merahbi R, Schmidt V, Karwen T, Loza-Valdes A, Strohmeyer A, Reuter S, Noh M, Wit M, Hawro I (2021) Protein Kinase D2 drives chylomicron-mediated lipid transport in the intestine and promotes obesity. *EMBO Mol Med* 13: e13548

© 2023 The Authors

Tunaru S, Bonnavion R, Brandenburger I, Preussner J, Thomas D, Scholich K, Offermanns S (2018) 20-HETE promotes glucose-stimulated insulin secretion in an autocrine manner through FFAR1. *Nat Commun* 9: 1–11

Turk J, Hughes JH, Easom RA, Wolf BA, Scharp DW, Lacy PE, McDaniel ML (1988) Arachidonic acid metabolism and insulin secretion by isolated human pancreatic islets. *Diabetes* 37: 992–996

Usui R, Yabe D, Fauzi M, Goto H, Botagarova A, Tokumoto S, Tatsuoka H, Tahara Y, Kobayashi S, Manabe T (2019) GPR40 activation initiates store-operated Ca^{2+} entry and potentiates insulin secretion via the IP3R1/STIM1/Orai1 pathway in pancreatic β-cells. *Sci Rep* 9: 1–11

Vettor R, Granzotto M, De Stefani D, Trevellin E, Rossato M, Farina MG, Milan G, Pilon C, Nigro A, Federspil G (2008) Loss-of-function mutation of the GPR40 gene associates with abnormal stimulated insulin secretion by acting on intracellular calcium mobilization. *J Clin Endocrinol Metabol* 93: 3541–3550

Villeda SA, Luo J, Mosher KI, Zou B, Britschgi M, Bieri G, Stan TM, Fainberg N, Ding Z, Eggel A (2011) The ageing systemic milieu negatively regulates neurogenesis and cognitive function. *Nature* 477: 90–94

Wettschureck N, Rütten H, Zywietz A, Gehring D, Wilkie TM, Chen J, Chien KR, Offermanns S (2001) Absence of pressure overload induced myocardial hypertrophy after conditional inactivation of Gα q/Gα 11 in cardiomyocytes. *Nat Med* 7: 1236–1240

Wit M, Trujillo-Viera J, Strohmeyer A, Klingenspor M, Hankir M, Sumara G (2022) When fat meets the gut—focus on intestinal lipid handling in metabolic health and disease. *EMBO Mol Med* 14: e14742

Wu J, Liu Q, Zhang X, Tan M, Li X, Liu P, Wu L, Jiao F, Lin Z, Wu X (2022) The interaction between STING and NCOA4 exacerbates lethal sepsis by orchestrating ferroptosis and inflammatory responses in macrophages. *Cell Death Dis* 13: 653

Yngen M, Li N, Hjemdahl P, Wallén NH (2001) Insulin enhances platelet activation *in vitro*. *Thromb Res* 104: 85–91

License: This is an open access article under the terms of the Creative Commons Attribution License, which permits use, distribution and reproduction in any medium, provided the original work is properly cited.

Expanded View Figures

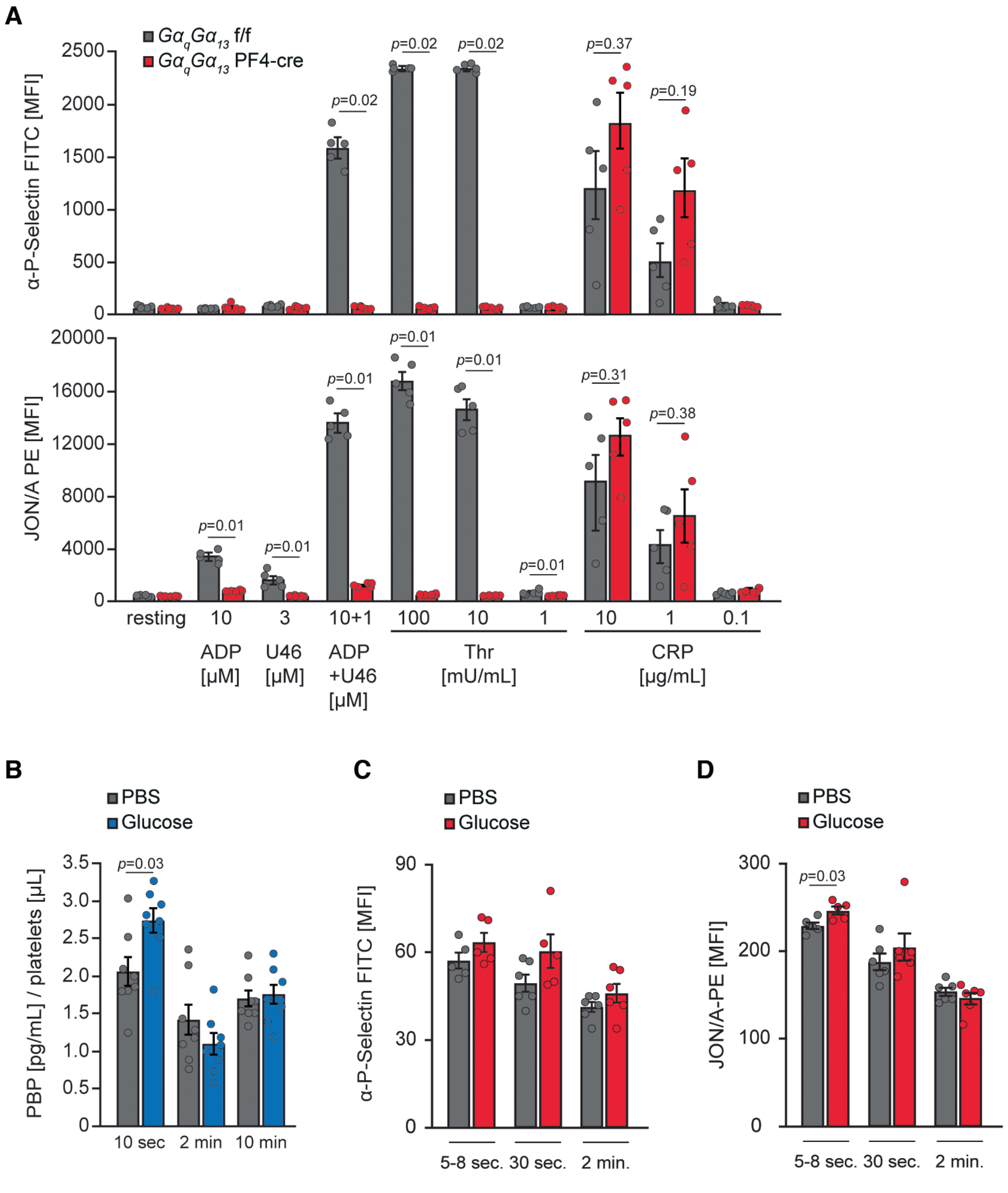

Figure EV1. Glucose promotes platelet activity.

A P-selectin exposure and integrin activation assessed by JONA PE antibody which recognizes an activated form of mouse platelet GPIIb/IIIa, of platelets from male GαqGα13 PF4 Δ/Δ and GαqGα13 f/f mice after stimulation with indicated agonists (determined by flow cytometry). GαqGα13 f/f, n = 5; GαqGα13 PF4 Δ/Δ, n = 5. U46, U46619 is a stable thromboxane A2 analog; ADP, Adenosine diphosphate; Thr, Thrombin; CRP, collagen-related peptide.

B–D Levels of platelet basic protein (PBP) assessed by specific ELISA (B), platelet surface exposure of P-Selectin (C) and integrins (D) (both assessed by flow cytometry using specific antibodies) in the blood of 10-week-old male C57BL/6JRj injected with glucose (2 g per kg of body weight) after indicated time points. PBP, n = 8; P-Selectin and integrins, n = 5.

Data information: Kruskal–Wallis test followed by Mann–Whitney test as *post hoc* analysis with Benjamini–Hochberg correction for multiple comparisons (A). Mann–Whitney test (B–D). Data are mean ± SEM. Each n represents the measurement of a sample from distinct mice.
Source data are available online for this figure.

 © 2023 The Authors

Figure EV2. Humoral factors and glucose define reciprocal relation between platelets and pancreatic β cells.

A Representative fluorescence and bright field microscopic images of platelet aggregates formed upon flow adhesion of whole blood from C57BL/6JRj male mice. Before perfusion, mouse blood was incubated for 5 min with supernatants of indicated cells or control supernatant (Ctr). For the generation of supernatants of the different cell types were incubated in the KRB containing 25 mM glucose for 3 min. Control medium have been generated by incubation of the same media on a cell-free culture dish coated with Matrigel.

B Representative bright-field microscopic image obtained during intra-vital imaging of C57BL/6JRj male mouse pancreas with highlighted localized pancreatic islet.

C Representative DAPI and immunostainings of the exocrine and endocrine C57BL/6JRj male mouse pancreas. Mice were i.v. injected with JAQ1 IgG 5 days before and with control IgG, p0p/B F(ab′)$_2$, JON/A F(ab′)$_2$, or R300 IgG 24 h before organ harvesting.

D Platelet count normalized to islet area. Control, n = 131 (six mice); JAQ1 IgG, n = 71 (six mice); p0p/B F(ab′)$_2$, n = 113 (six mice); JON/A F(ab′)$_2$, n = 77 (six mice), R300 IgG, n = 27 (three mice).

Data information: Each n represents an image of an islet. Kruskal–Wallis test followed by Mann–Whitney test as *post hoc* analysis with Benjamini–Hochberg correction for multiple comparisons. Data in boxplot: center line shows median; cross indicates mean; box defines first and third quartiles; whiskers indicate 1.5 × interquartile range; outliers are individually plotted.
Source data are available online for this figure.

© 2023 The Authors

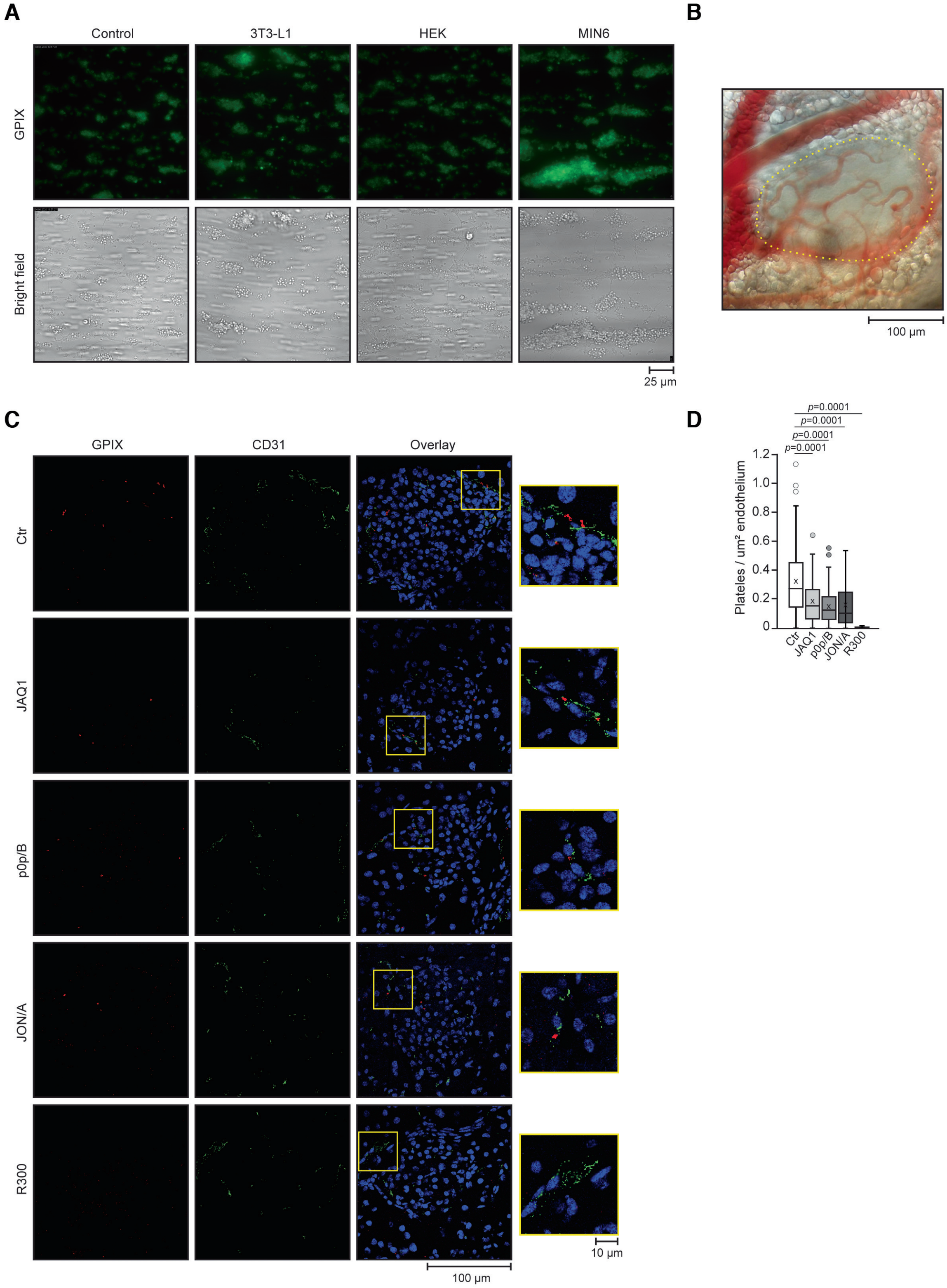

Figure EV2.

 © 2023 The Authors

Figure EV3. Platelets do not affect β cell mass.

A Glucose stimulated (3 g per kg of body weight) release of c-peptide in GαqGα13 f/f and GαqGα13 PF4 Δ/Δ (males, 10 weeks old, *n* = 4 for GαqGα13 f/f and 4 for GαqGα13 PF4 Δ/Δ).

B Quantification of area under the curve (AUC) from (A).

C Glucose tolerance test (2 g per kg of body weight) in GαqGα13 f/f and GαqGα13 PF4 Δ/Δ (females, 10 weeks old, *n* = 5 GαqGα13 f/f and 8 for GαqGα13 PF4 Δ/Δ).

D–F The ratio of islet area to whole pancreas area of BMC-Gp1bα$^{-/-,TG}$ (D), BMC-Gp6$^{-/-}$ (E), and GαqGα13 PF4 Δ/Δ (F) male mice relative to the respective wild-type control (ctr). BMC-Gp1bα$^{+/+}$, *n* = 32 (eight mice, 21 weeks old); BMC-Gp1bα$^{-/-,TG}$, *n* = 28 (seven mice, 21 weeks old D). BMC-Gp6$^{+/+}$, *n* = 24 (eight mice, 22 weeks old); BMC-Gp6$^{-/-}$, *n* = 18 (six mice, 22 weeks old E). GαqGα13 f/f, *n* = 15 (five mice, 15 weeks old); GαqGα13 PF4 Δ/Δ, *n* = 12 (four mice, 15 weeks old F). Each *n* represents the ratio of the islet area to the pancreas area of one tissue section.

G Insulin content of pancreas from GαqGα13 PF4 Δ/Δ and GαqGα13 f/f 9 weeks old male mice normalized to total protein. GαqGα13 f/f, *n* = 8; GαqGα13 PF4 Δ/Δ, *n* = 8. Each *n* represents the measurement of a sample from distinct mice.

H Glucagon serum levels of overnight fasted GαqGα13 PF4 Δ/Δ and GαqGα13 f/f 14 weeks old male mice. GαqGα13 f/f, *n* = 12; GαqGα13 PF4 Δ/Δ, *n* = 11. Each *n* represents the measurement of a sample from distinct mice.

I Insulin release per islet isolated from GαqGα13 PF4 Δ/Δ and GαqGα13 f/f 8–12-week-old male mice upon 2.8 mM (LG) and 16.7 mM glucose (HG). GαqGα13 f/f, *n* = 16; GαqGα13 PF4 Δ/Δ, *n* = 38. Each *n* represents an independent biological replicate.

J, K Body weight and composition of Gp6$^{-/-}$ 24 weeks old (J) and 15-week-old GαqGα13 PF4 Δ/Δ (K) mice with respective control. Gp6$^{+/+}$, *n* = 4; Gp6$^{-/-}$, *n* = 4 (J). GαqGα13 f/f, *n* = 9; GαqGα13 PF4 Δ/Δ, *n* = 4 (K). Each *n* represents the measurement of a sample from distinct mice.

Data information: Mann–Whitney test (A–H). Kruskal–Wallis test followed by Mann–Whitney test as *post hoc* analysis with Benjamini–Hochberg correction for multiple comparisons (I–K). Data are mean ± SEM. Data in boxplots: center line shows median; cross indicates mean; box defines first and third quartiles; whiskers indicate 1.5 × interquartile range; outliers are individually plotted (D–F, H, I).
Source data are available online for this figure.

© 2023 The Authors

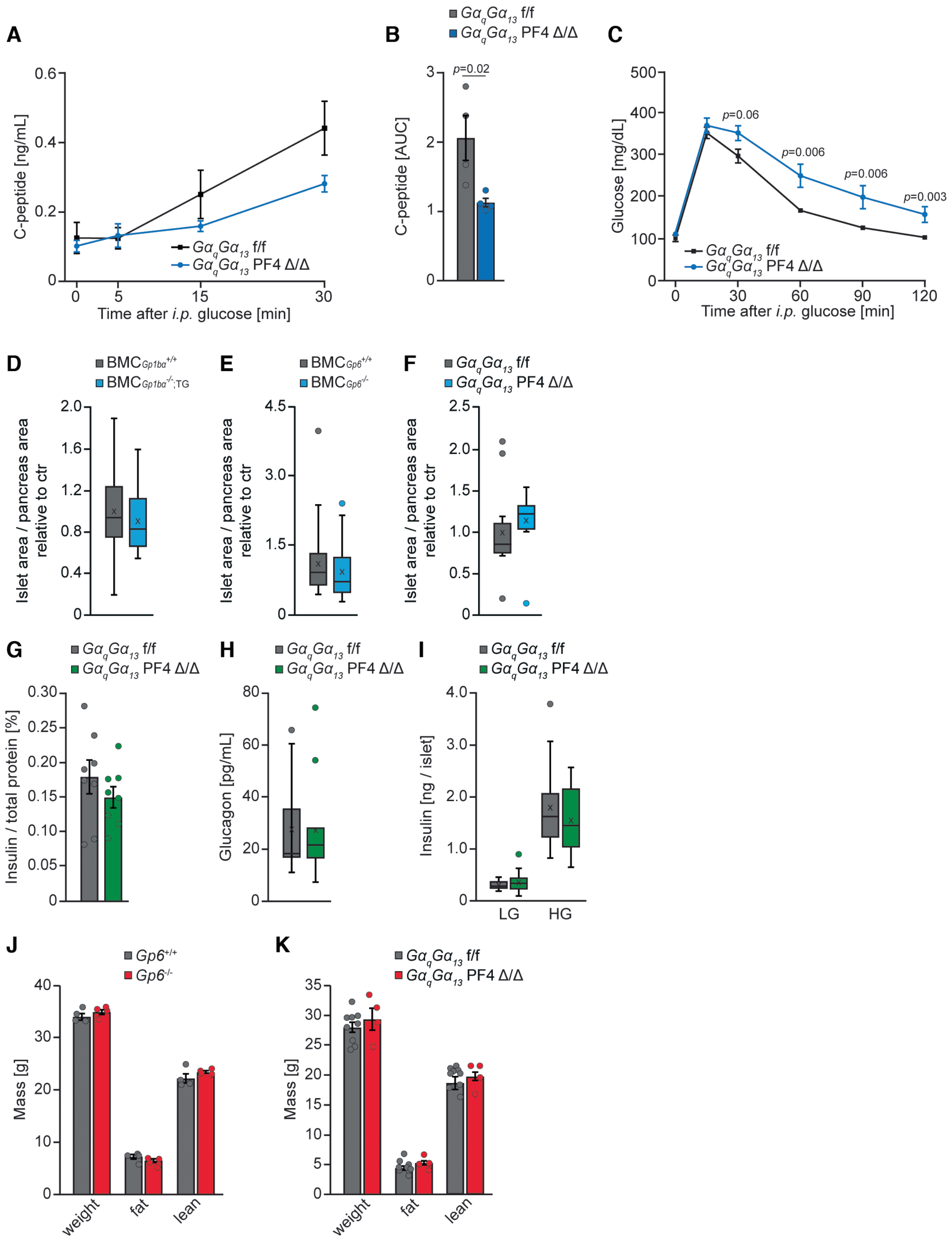

Figure EV3.

 © 2023 The Authors

Figure EV4. A platelet-derived factor stimulates insulin secretion.

A, B Cell surface expression of GPVI (A) and platelet count (B) of 6-week-old, male mice treated with JAQ1-IgG antibody for 5 days (*n* = 11). Each *n* represents one injected mouse.

C Cell surface expression of GPVI assessed by flow cytometry using JAQ1-FITC and GPIIb/IIIa verified by the same method using JON/A-FITC antibody in control animals and 6-week-old male mice injected with 4 mg per kg body weight JAQ1-F(ab)$_2$ or JON/A-F(ab)$_2$ 24 h before an experiment (*n* = 4). Each *n* represents one injected mouse.

D Platelet count in 6-week-old male mice treated with R300 antibody (2 mg per kg of body weight) 24 h before the experiment. Ctrl IgG, *n* = 11; R300, *n* = 10. Each *n* represents one injected mouse.

E Glucose tolerance test (2 g per kg of body weight) of 6-week-old female mice treated R300 or control IgG antibody (2 mg per kg of body weight) for 24 h before the experiment. Each *n* represents one injected mouse.

F, G Western blot (WB) analyses using indicated antibodies of extracts isolated from skeletal muscles (quadriceps) (F) and perigonadal adipose tissue (G) of 10-week-old male mice depleted from platelets (using R300 antibody) or corresponding age and sex-matched animals. Each band on the WB corresponds to the tissue isolated from one mouse.

H Insulin secretion from INS1 cells stimulated with a supernatant of activated human platelets (hPS) for indicated time points or control buffer in the presence of 2.8 mM glucose (*n* = 4). Each *n* represents an independent biological replicate.

I Insulin secretion of human EndoC-βH1 cells after 15 min of stimulation with supernatant of activated human platelets (hPS) or control buffer upon 2.5 mM glucose (*n* = 3). Each *n* represents an independent biological replicate.

Data information: Mann–Whitney test (A–E, I). Kruskal–Wallis test followed by Mann–Whitney test as *post hoc* analysis with Benjamini–Hochberg correction for multiple comparisons (H). Data are mean ± SEM.
Source data are available online for this figure.

© 2023 The Authors

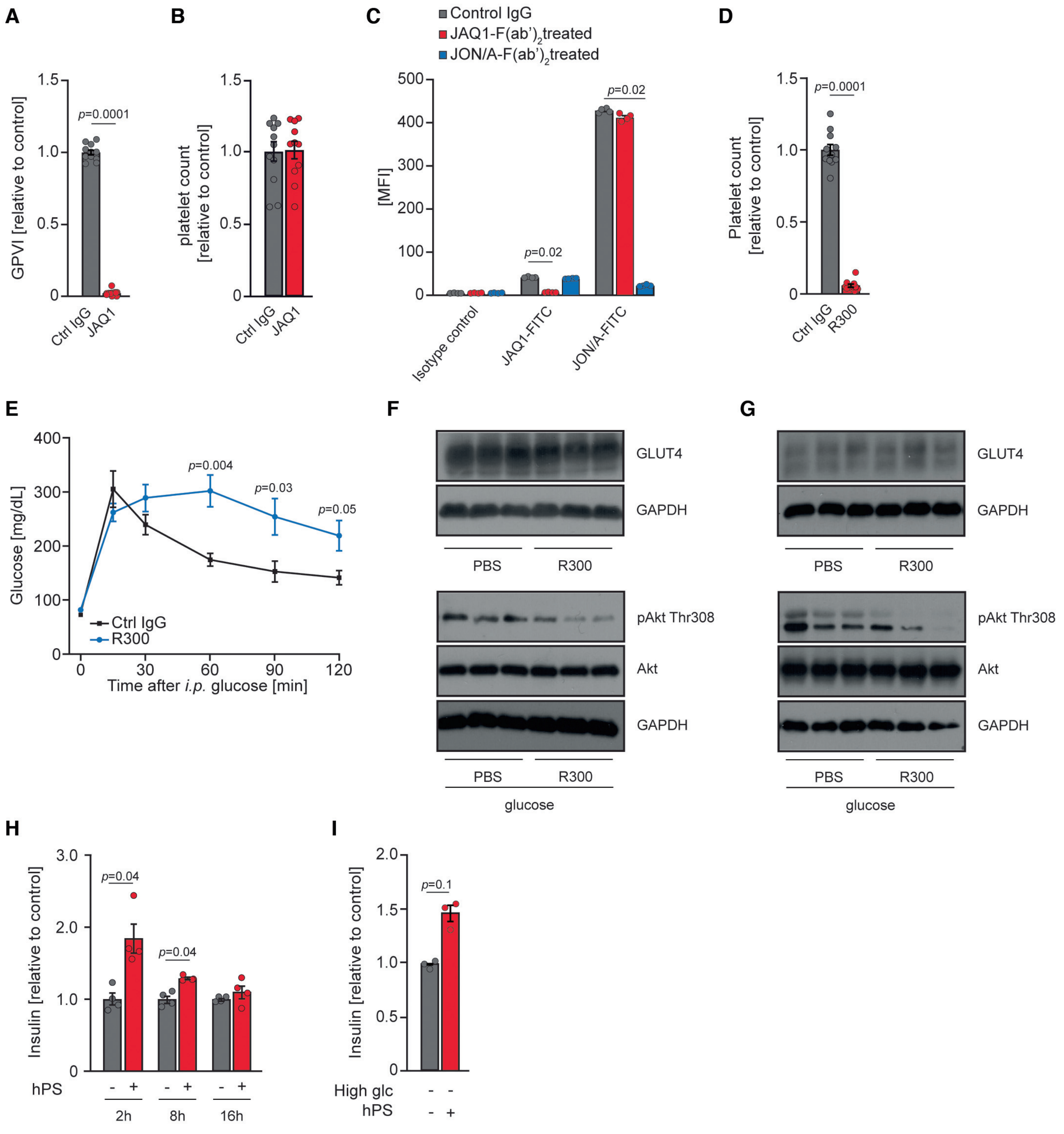

Figure EV4.

© 2023 The Authors

Figure EV5. The impact of platelets on insulin secretion declines with age.

A Insulin tolerance test of 13-week-old C57BL/6JRj male mice treated with clopidogrel for 7 weeks. Control, n = 7; Clopidogrel, n = 9.

B Western blot (WB) analysis using indicated antibodies on platelets isolated from 8 weeks control and clopidogrel-treated C57BL/6JRj male mice for 3 weeks. Each line represents a sample from distinct mice.

C Glucose tolerance test (2 g per kg of body weight) of 9-week-old C57BL/6JRj female mice treated with clopidogrel for 3 weeks. Control, n = 15; Clopidogrel, n = 14.

D Glucose tolerance test (2 g per kg of body weight) of 16-week-old WISTAR rats treated with clopidogrel for 8 weeks. Control, n = 7; Clopidogrel, n = 10.

E Glucose-stimulated insulin secretion (2 g per kg of body weight) of 17-week-old WISTAR rats treated with clopidogrel for 9 weeks. Control, n = 9; Clopidogrel, n = 9.

F Insulin tolerance test of 18-week-old WISTAR rats treated with clopidogrel for 10 weeks. Control, n = 8; Clopidogrel, n = 10.

G P-selectin exposure and integrin activation assessed by flow cytometry using specific antibodies on platelets isolated from young (9 weeks old, n = 4) and aged (60 weeks old, n = 4) male mice. U46, U46619 is a stable thromboxane A2 analog; ADP, Adenosine diphosphate; Thr, Thrombin; CRP, collagen-related peptide.

H–J Glucose tolerance test (2 g per kg of body weight) on treated with clopidogrel for 3 weeks or control mice. Fourteen weeks old male mice depleted from platelets (n = 10) and corresponding control animals (n = 9) (H) 13-week-old male mice treated with clopidogrel (n = 9) or control solution (n = 9) (I) and 14-week-old male GαqGα13 PF4 Δ/Δ mice (n = 5) as well as corresponding control GαqGα13 f/f animals (n = 4) (J). All animals were subjected to HFD feeding for 8 weeks before the glucose tolerance test.

Data information: Each n represents the measurement of a sample from distinct mice or rats. Mann–Whitney test. Data are mean $\pm$ SEM.
Source data are available online for this figure.

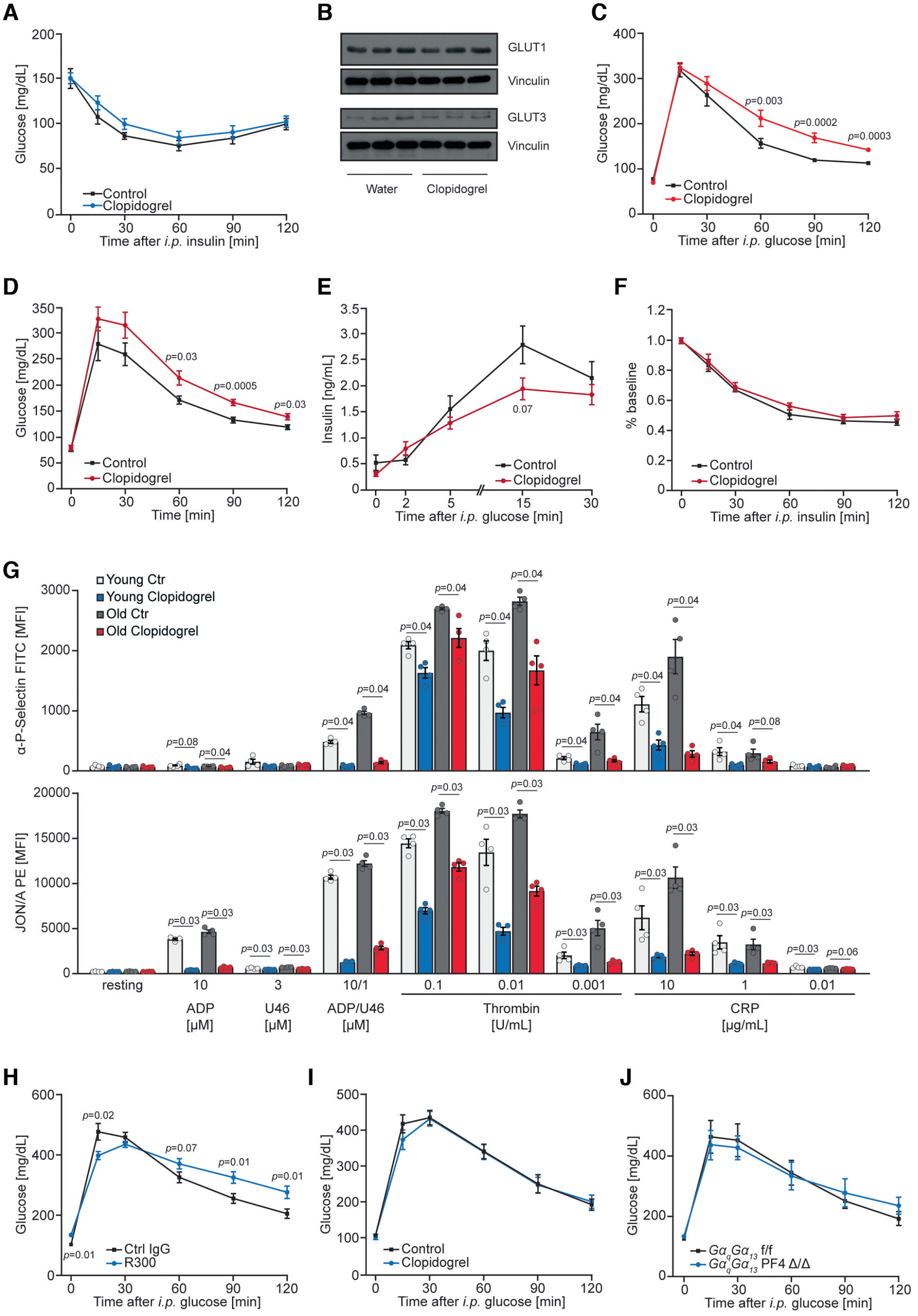

Figure EV5.

 © 2023 The Authors

Table of content

Appendix Figure S1 2
Appendix Figure S2. 4

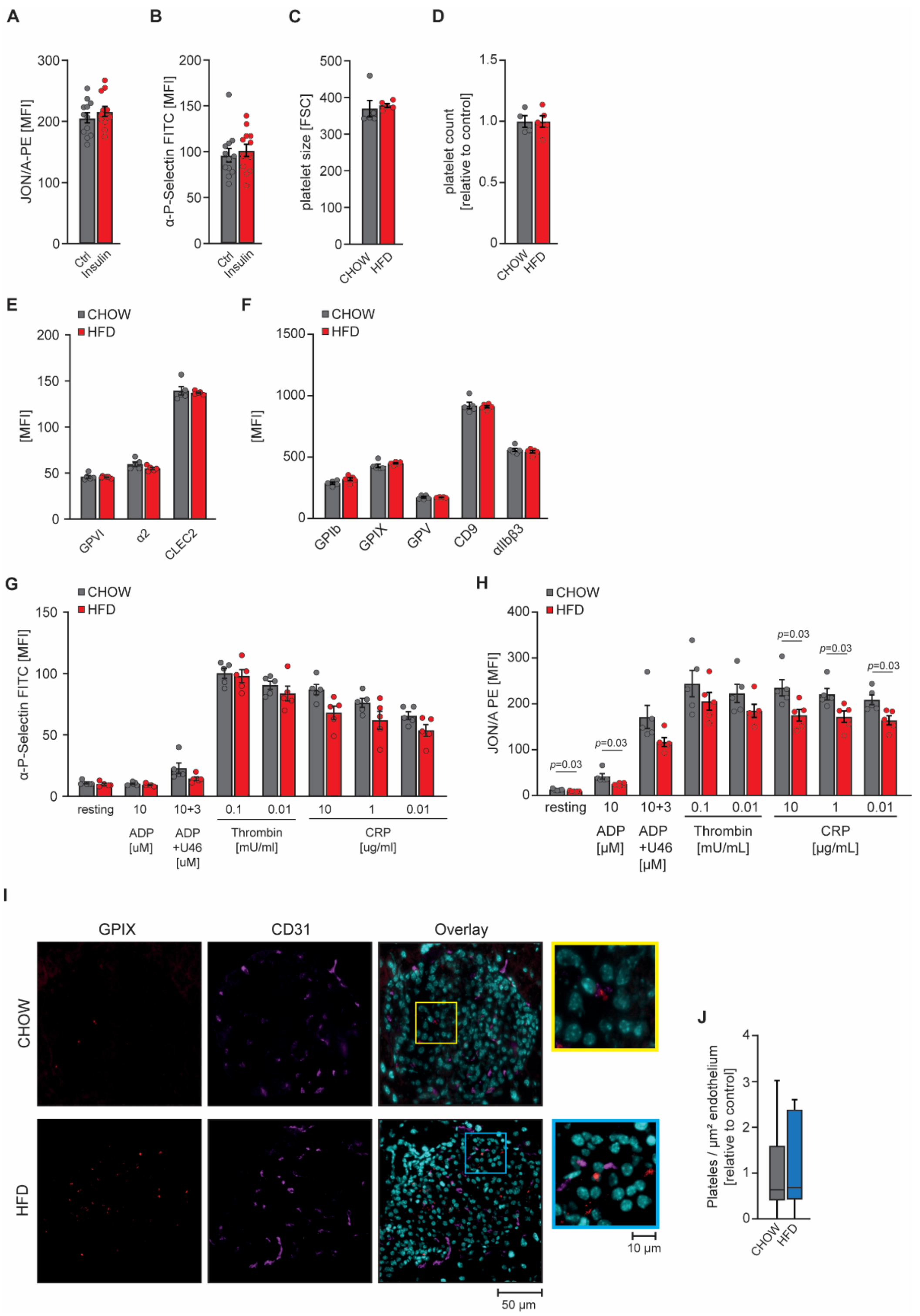

Appendix Figure S1. Insulin and high-fat diet (HFD) feeding do not affect platelet activity.

(**A** and **B**) Integrin activation (**A**) and P-selectin exposure (**B**) of C57BL/6JRj mouse platelets preincubated for 12 min. with 5 ng/ml insulin and 15 mM glucose determined by flow cytometry (n=12). Each n represents the measurement of a sample from a different experiment.

(**C** and **D**) Size (**C**)**,** and count (**D**) of platelets from 16 weeks old male C57BL/6JRj mice fed for the last 12 weeks with regular chow or high fat diet (HFD). (C) CHOW, n=4; HFD, n=5. (D) n=5. Each n represents the measurement of a sample from distinct mice.

(**E** and **F)** Surface expression of indicated integrins, glycoproteins, and receptors on platelets isolated from the mice described above (n=5). Each n represents the measurement of a sample from distinct mice. Glycoprotein Ib (GPIb), Glycoprotein IX (GPIX), Glycoprotein V (GPV), CD9 antigen (CD9), integrin alpha IIb/beta3 (GPIIb/IIIa), Glycoprotein VI (GPVI), Integrin alpha-2 (α2), C-type lectin-like receptor 2 (CLEC2).

(**G** and **H**) P-selectin exposure (**G**) and integrin (**H**) activation assessed by JON/A PE antibody which recognizes activated form of mouse platelet GPIIb/IIIa of platelets isolated from mice described in C and D (n=5). Each n represents the measurement of a sample from distinct mice. U46 - U46619 is a stable thromboxane A2 analog, ADP - Adenosine diphosphate, Thr -Thrombin, CRP - collagen-related peptide

(**I**) Staining of the pancreas from 15 weeks old male mice fed a normal chow diet (CHOW, n=3) or high hat diet (HFD, n=3) for 10 weeks. Antibody against Glycoprotein IX (GPIX) was used to visualize platelets (red), and the endothelium was visualized by CD31 antibody (magenta). Islets were identified by increased nuclear density (nucleus of the cells visualized by DAPI in blue). (**J**) Quantification of the platelets in islets corresponding to Figure I (n=30). Each n represents an image of randomly selected islet.

Mann Whitney test (A-D, J). Kruskal Wallis test followed by Mann Whitney test as post-hoc analysis with Benjamini-Hochberg correction for multiple comparisons (**E-H**). Data are mean ± SEM. Data in boxplots: the center line shows median; box defines first and third quartiles; whiskers indicate 1.5 × interquartile range; outliers are individually plotted (**J**).

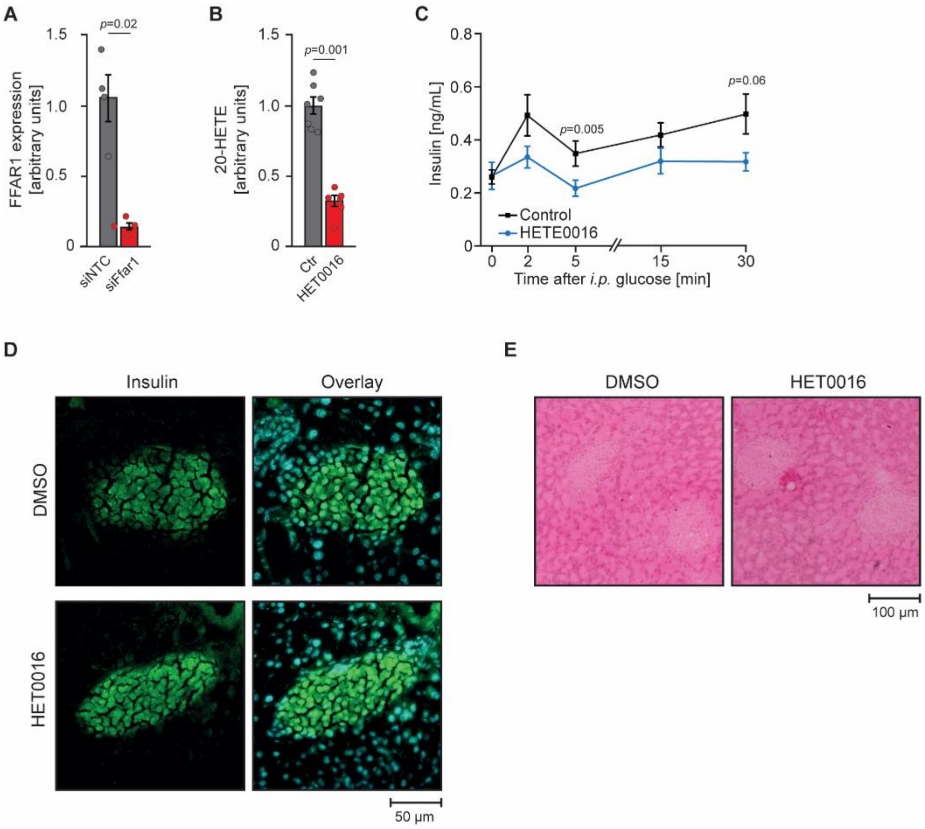

Appendix Figure S2. Inhibition of 20-HETE production reduces glucose-stimulated insulin secretion

(**A**) Relative FFAR1 expression in INS1 cells transfected with FFAR1 siRNA or non-targeting control (NTC) siRNA. (n=4) Each n represents an independent biological replicate.

(**B**) Relative serum levels of 20-HETE from C57BL/6JRj male mice injected i.p. with 10 mg per kg of body weight HET0016 72 h, 48 h, 24 h, and 30 min before the assay with respective control (Ctr). Ctr, n=7; HET0016, n=6. Each n represents the measurement of a sample from distinct mice.

(**C**) Glucose-stimulated insulin secretion on 8 weeks old male mice treated with 10 mg per kg of body weight HET0016 72 h, 48 h, 24 h, and 30 min before the experiment (n=11).

(**D** and **E**) Staining of pancreatic islets with insulin antibody (**D**) and pancreas with H&E staining (**E**) on mice described in figure C.

Mann Whitney test. Data are mean ± SEM.
