# Supplementary figures and images for: Platelet‐derived lipids promote insulin secretion of pancreatic β cells
Source: EMBO Mol Med. 2023 Jul 25;15(9):e16858. doi: 10.15252/emmm.202216858 (PMC10493578; doi:10.15252/emmm.202216858)

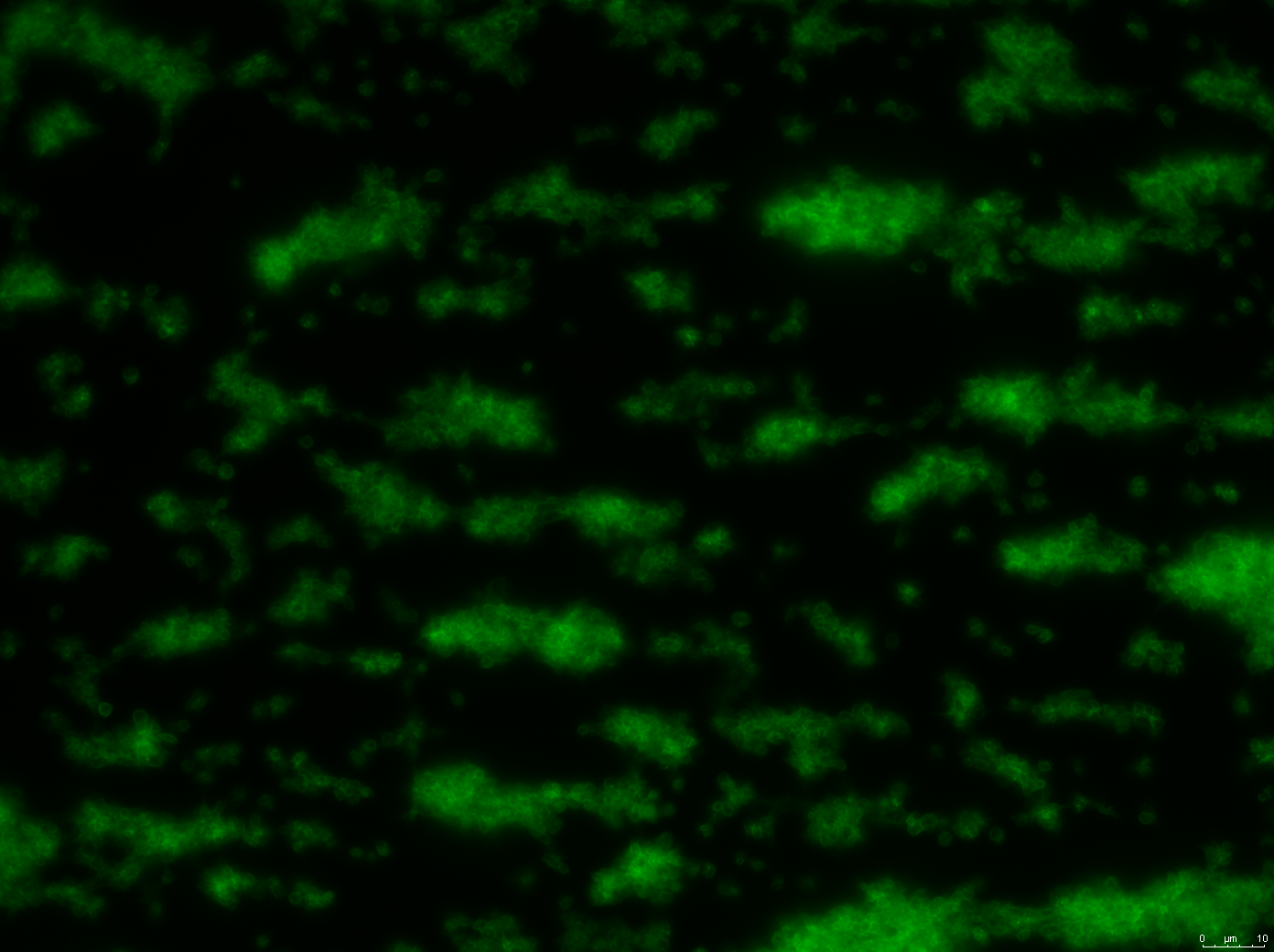

Supplement: Supplementary file 5 — Source Data for Figure 1 [file EMMM-15-e16858-s007.zip › Figure 1/1A/C57BL6J 2_8mM glucose GPIX.tif]

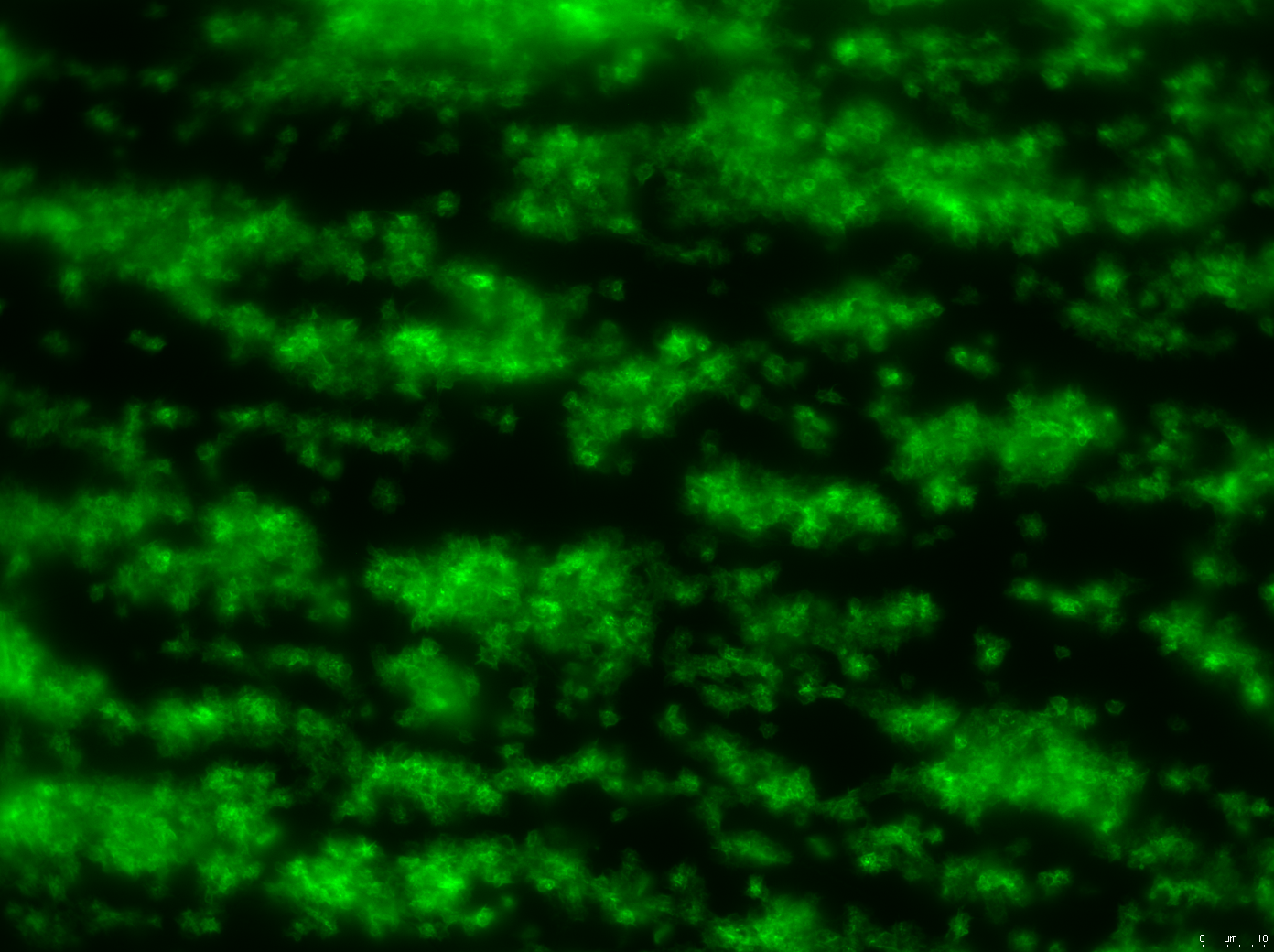

Supplement: Supplementary file 5 — Source Data for Figure 1 [file EMMM-15-e16858-s007.zip › Figure 1/1A/C57BL6J 25mM glucose GPIX.tif]

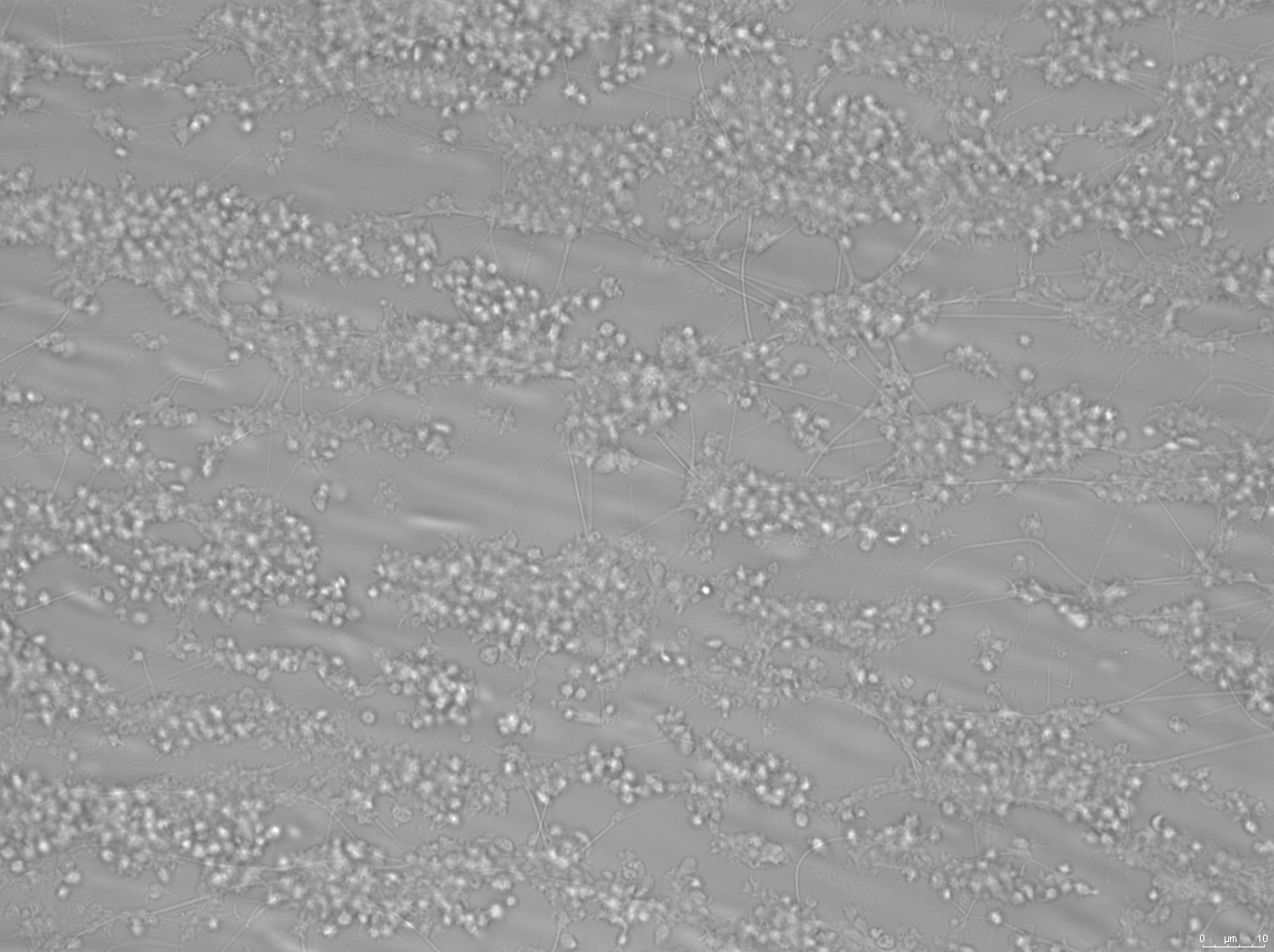

Supplement: Supplementary file 5 — Source Data for Figure 1 [file EMMM-15-e16858-s007.zip › Figure 1/1A/C57BL6J 25mM glucose Brightfield.tif]

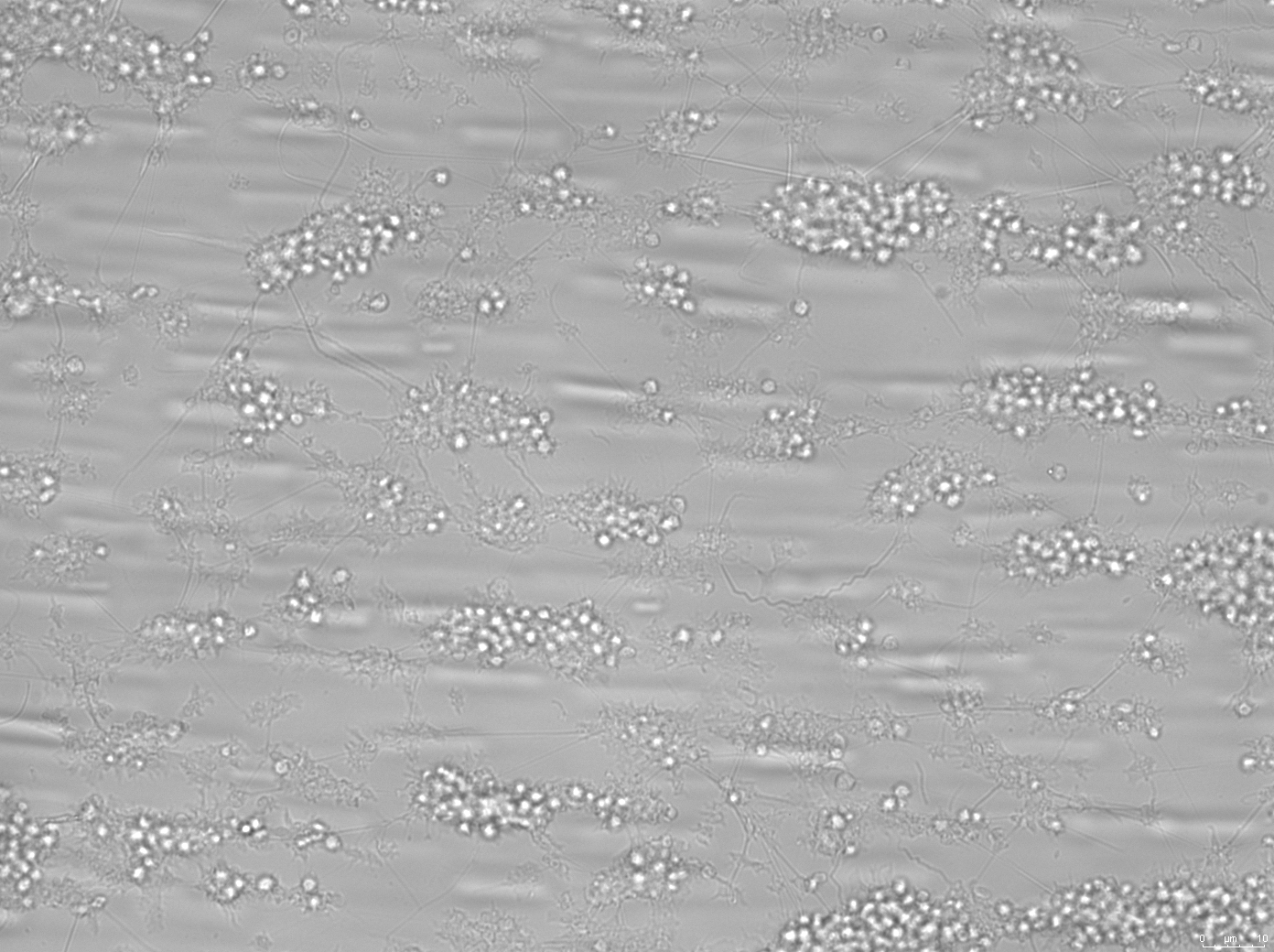

Supplement: Supplementary file 5 — Source Data for Figure 1 [file EMMM-15-e16858-s007.zip › Figure 1/1A/C57BL6J 2_8mM glucose Brightfield.tif]

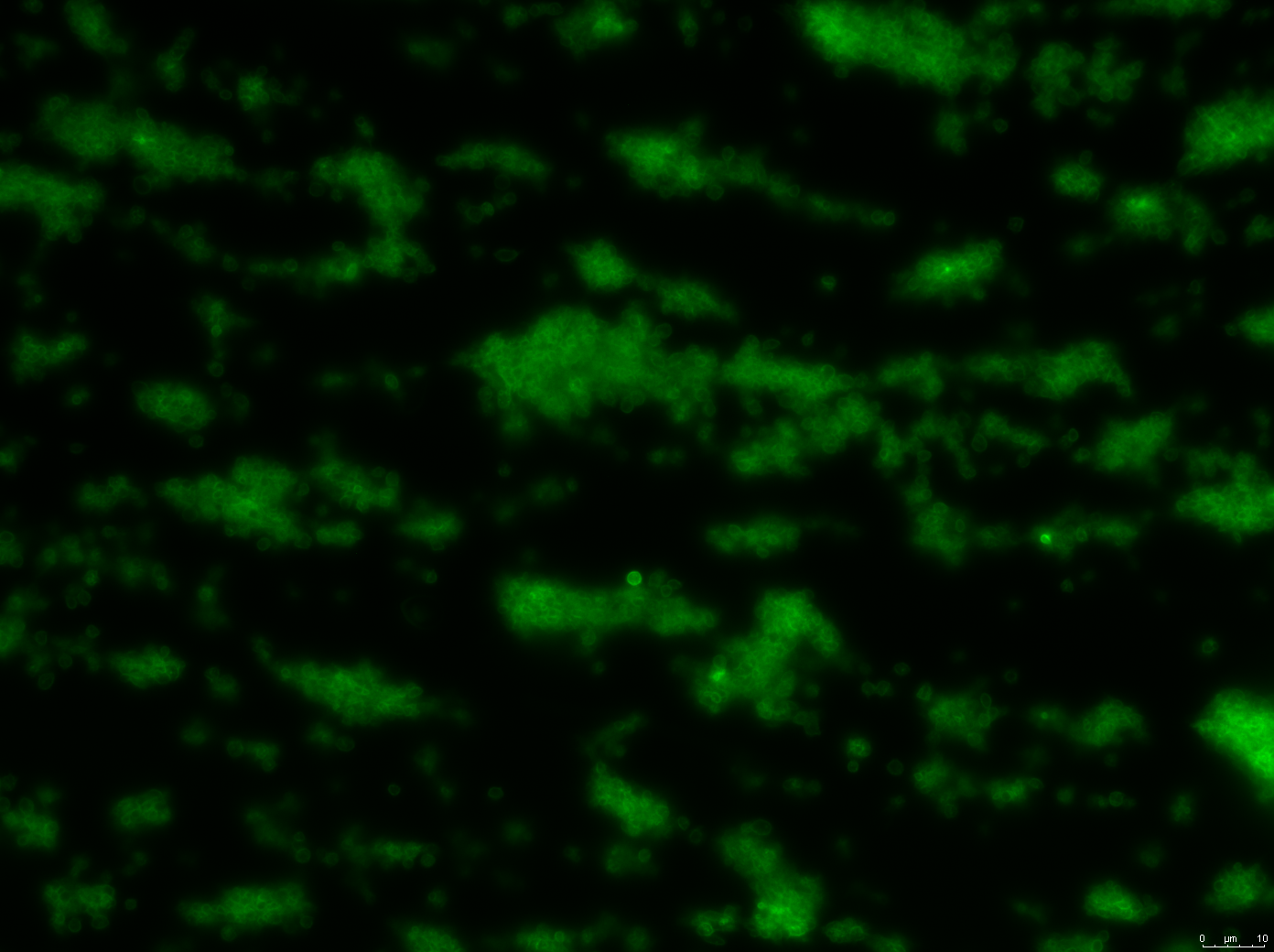

Supplement: Supplementary file 5 — Source Data for Figure 1 [file EMMM-15-e16858-s007.zip › Figure 1/1A/C57BL6J 5mM glucose GPIX.tif]

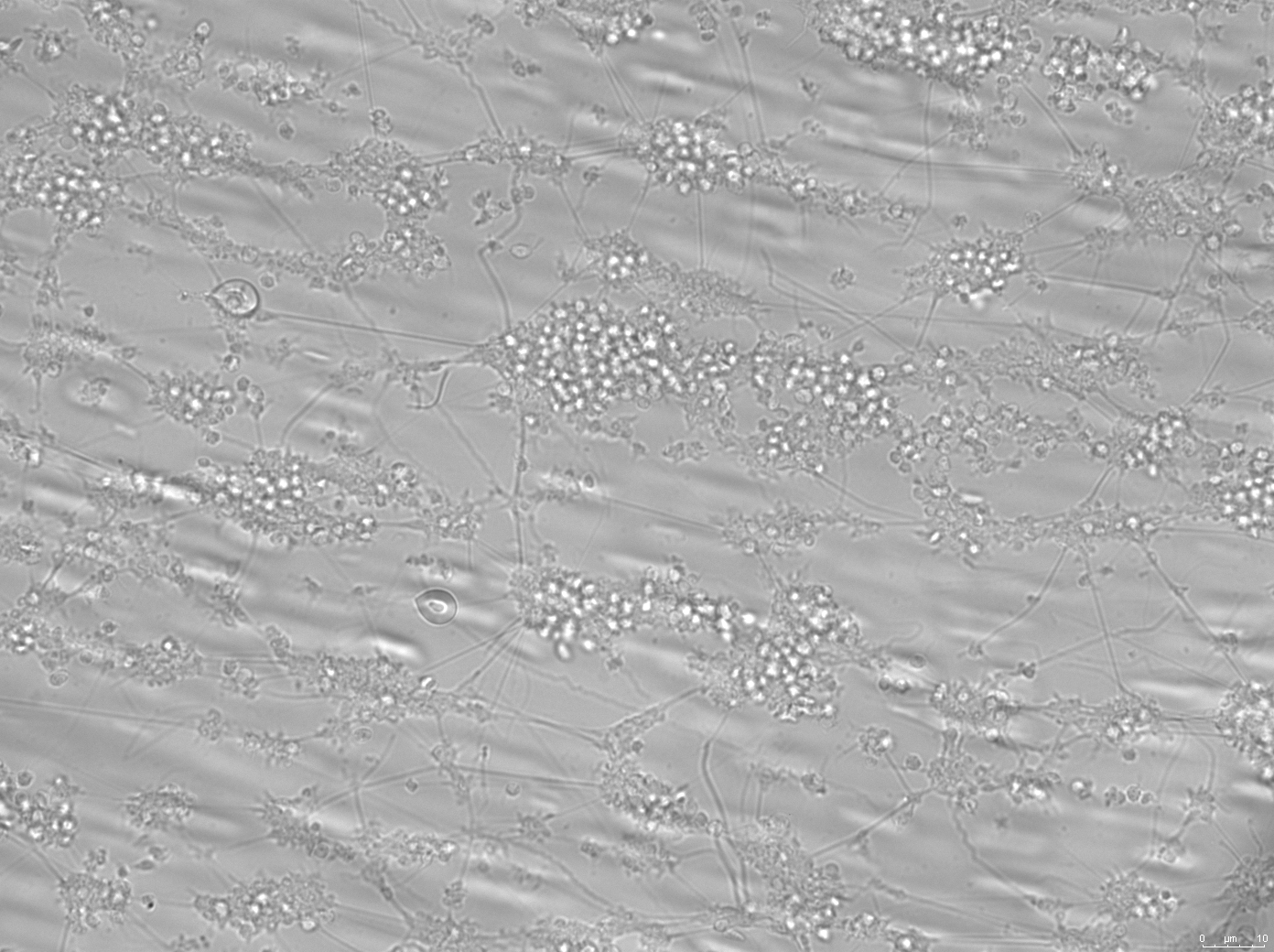

Supplement: Supplementary file 5 — Source Data for Figure 1 [file EMMM-15-e16858-s007.zip › Figure 1/1A/C57BL6J 5mM glucose Brightfield.tif]

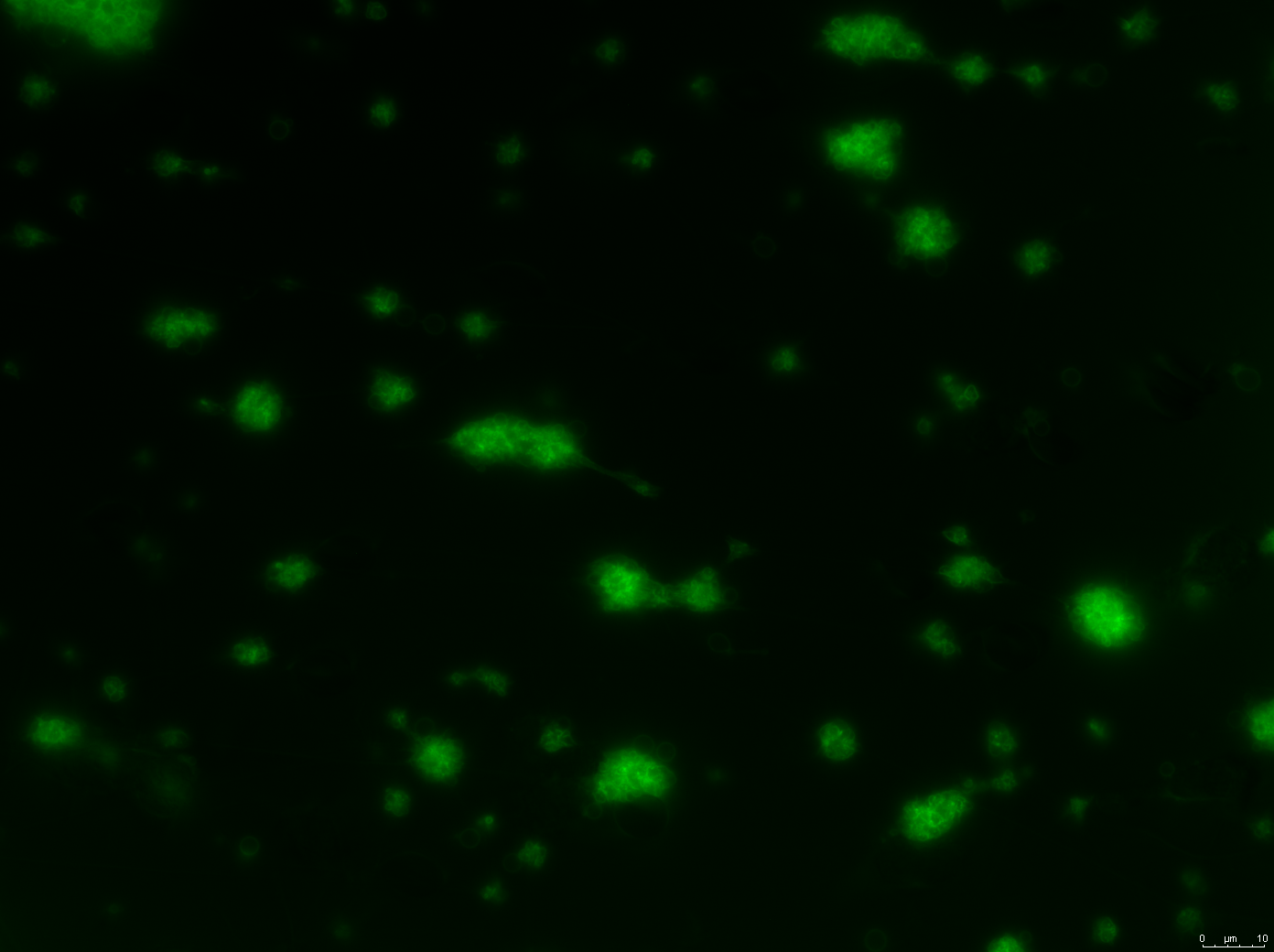

Supplement: Supplementary file 5 — Source Data for Figure 1 [file EMMM-15-e16858-s007.zip › Figure 1/1D/Human 2_8mMglucose GPIbBeta.tif]

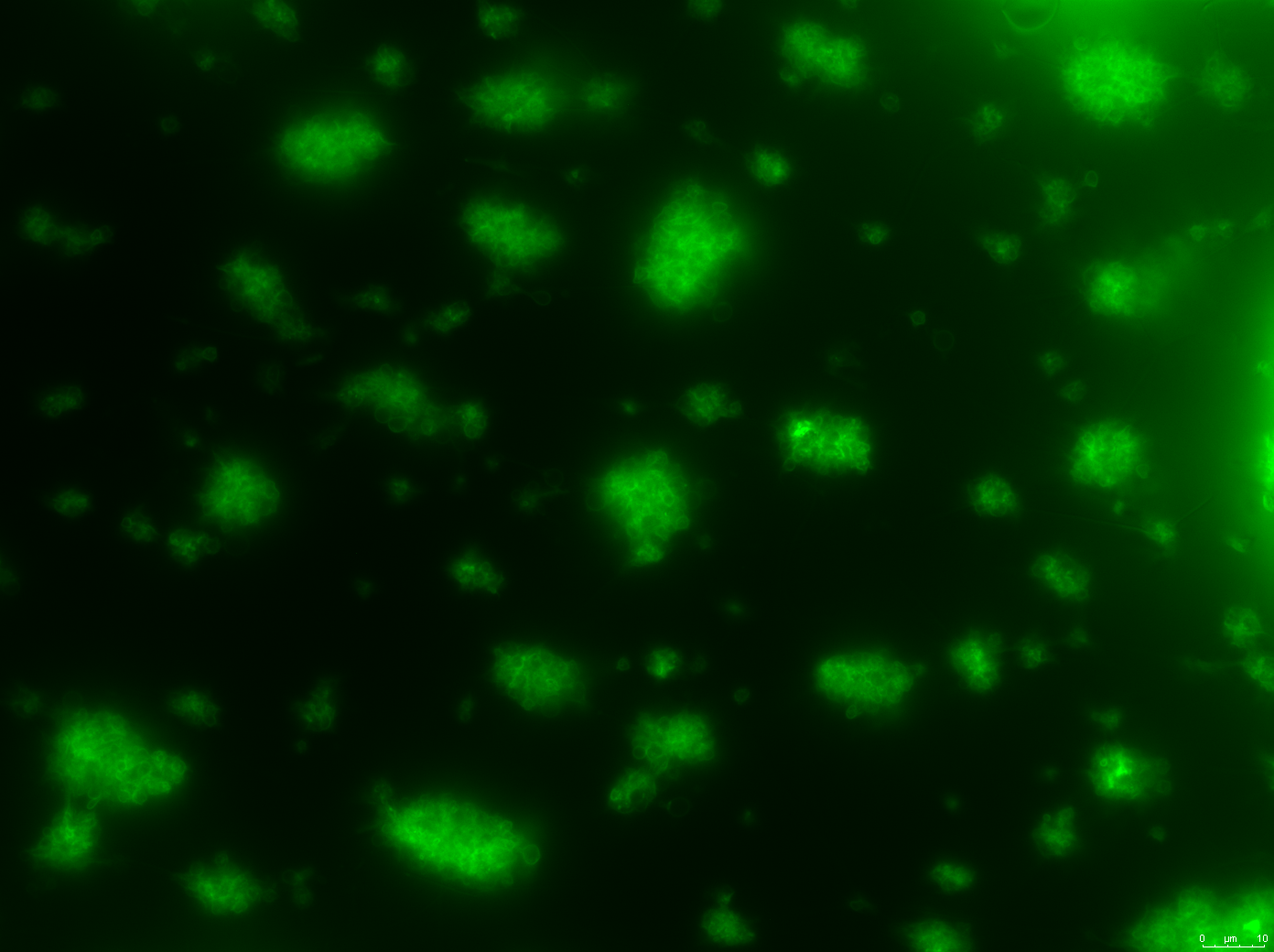

Supplement: Supplementary file 5 — Source Data for Figure 1 [file EMMM-15-e16858-s007.zip › Figure 1/1D/Human 25mMglucose GPIbBeta.tif]

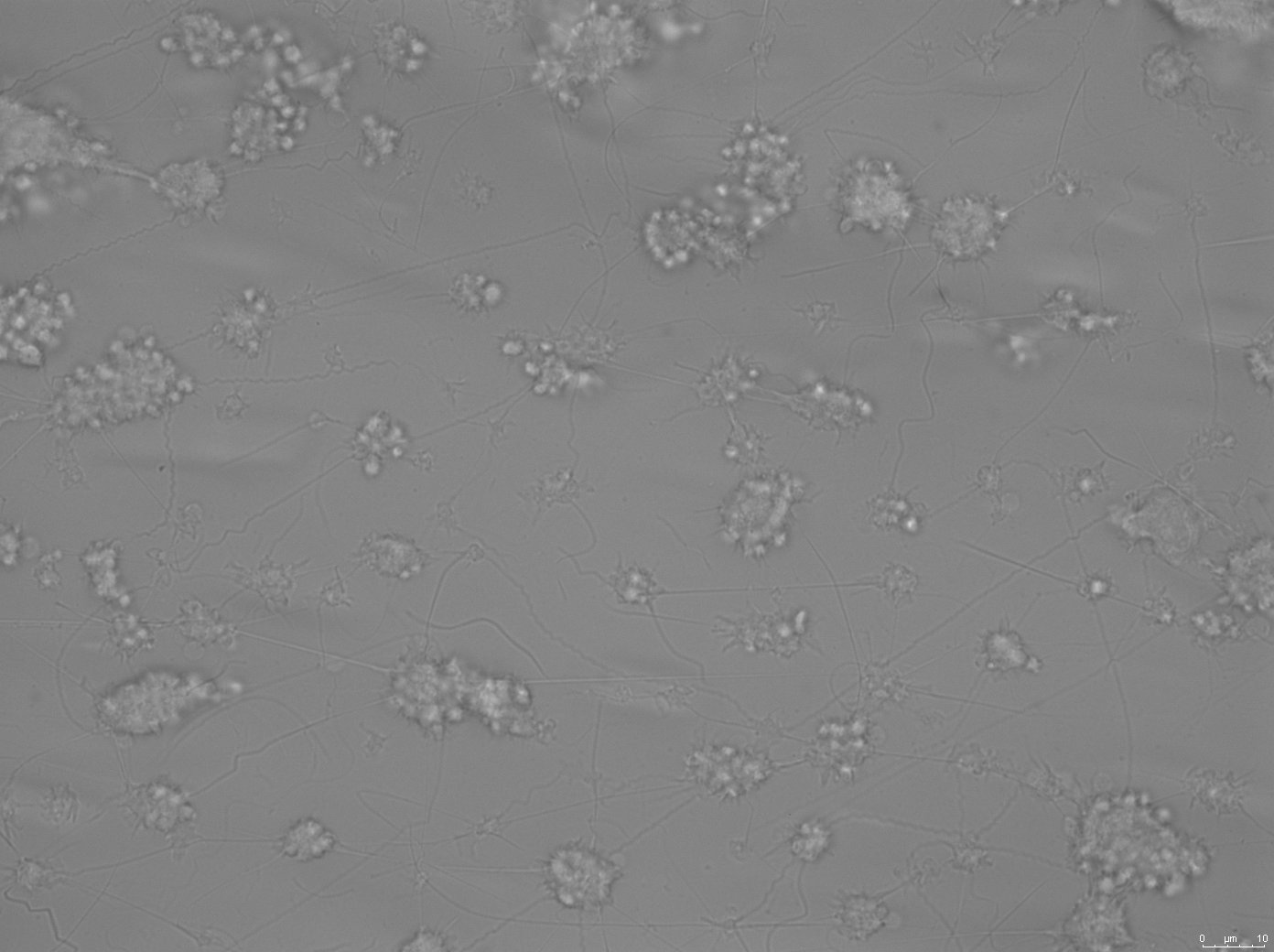

Supplement: Supplementary file 5 — Source Data for Figure 1 [file EMMM-15-e16858-s007.zip › Figure 1/1D/Human 25mMglucose Brightfield.tif]

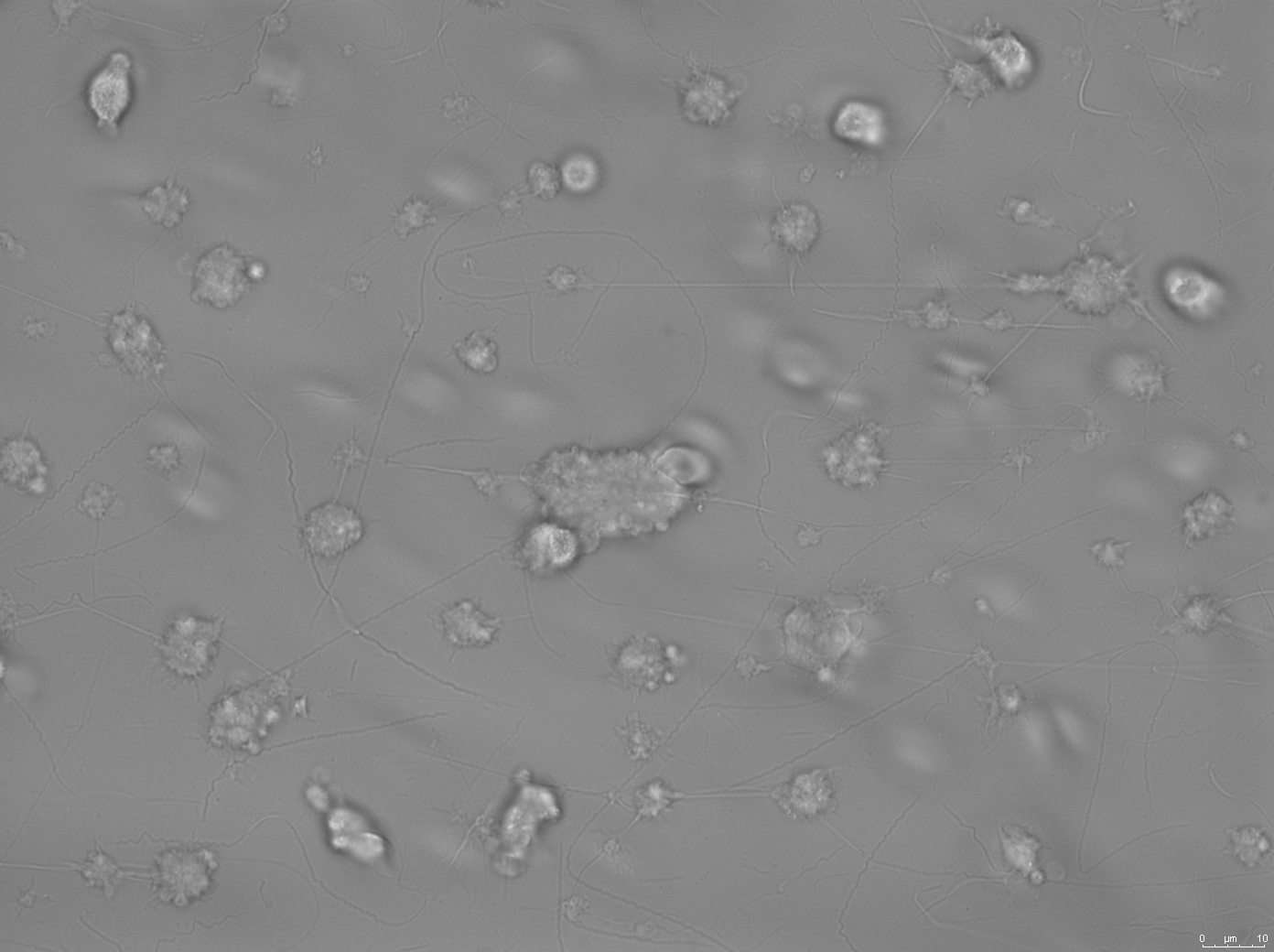

Supplement: Supplementary file 5 — Source Data for Figure 1 [file EMMM-15-e16858-s007.zip › Figure 1/1D/Human 5mMglucose Brightfield.tif]

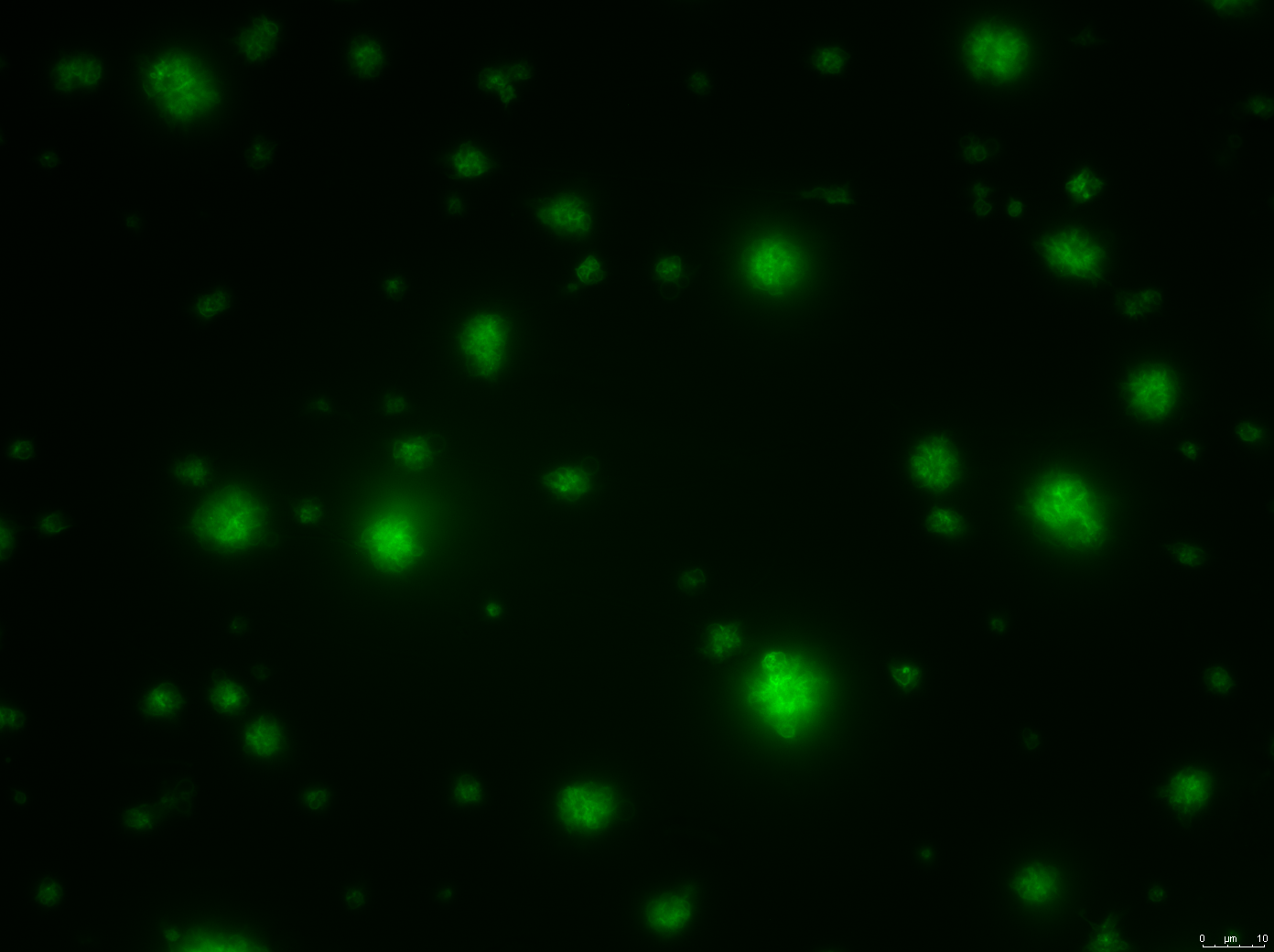

Supplement: Supplementary file 5 — Source Data for Figure 1 [file EMMM-15-e16858-s007.zip › Figure 1/1D/Human 5mMglucose GPIbBeta.tif]

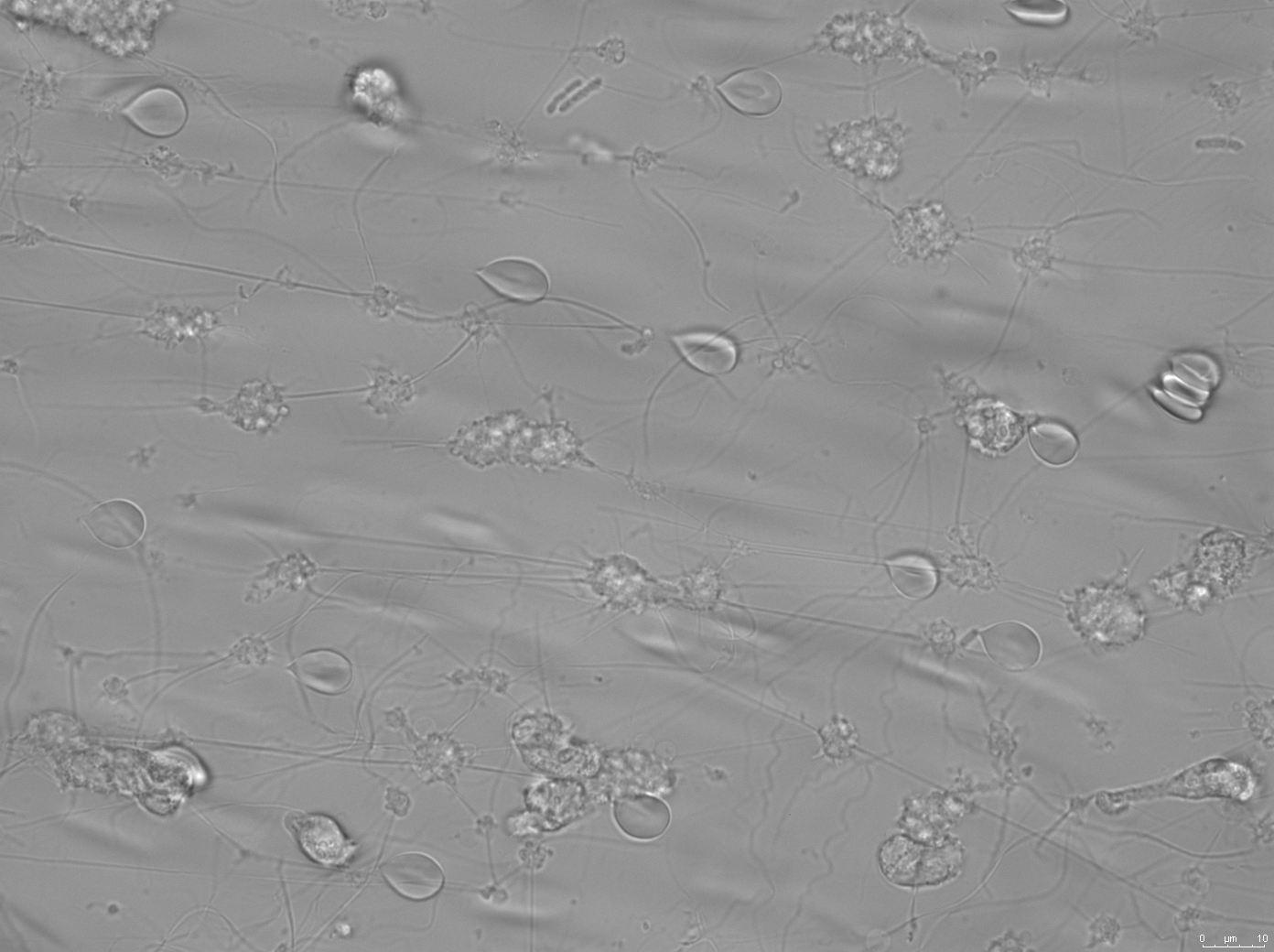

Supplement: Supplementary file 5 — Source Data for Figure 1 [file EMMM-15-e16858-s007.zip › Figure 1/1D/Human 2_8mMglucose Brightfield.tif]

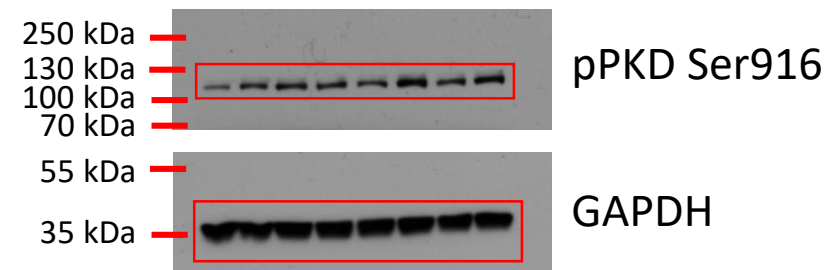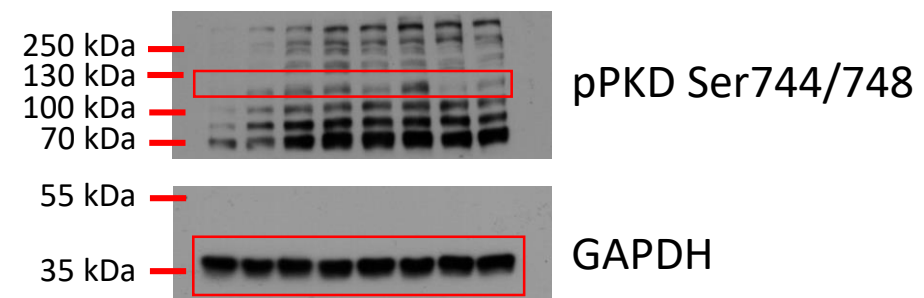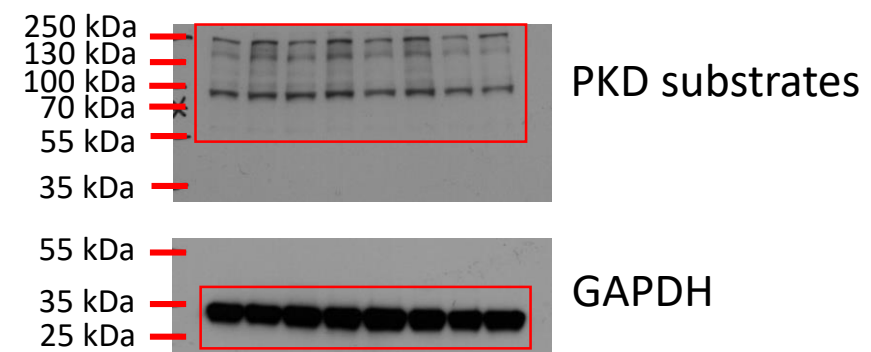

Supplement: Supplementary file 11 — Source Data for Figure 7 [file EMMM-15-e16858-s013.zip › Figure 7/7G.pdf]
