# Supplementary material for: Platelet‐derived lipids promote insulin secretion of pancreatic β cells
Source: EMBO Mol Med. 2023 Jul 25;15(9):e16858. doi: 10.15252/emmm.202216858 (PMC10493578; doi:10.15252/emmm.202216858)
Supplement: Supplementary file 14 [file EMMM-15-e16858-s012.pdf]

EMBO Molecular Medicine

Platelet-derived lipids promote insulin secretion of pancreatic β-cells

Till Karwen, Katarzyna Kolczynska, Carina Gross, Mona Löffler, Mike Friedrich, Angel Loza Valdes, Werner Schmitz, Magdalena Wit, Filip Dziaczkowski, Andrei Belykh, Jonathan Trujillo-Viera, Rabih El-Merahbi, Carsten Deppermann, Sameena Nawaz, Benoit Hastoy, Agnieszka Demczuk, Manuela Erk, Mariusz Wieckowski, Patrik Rorsman, Katrin Heinze, David Stegner, Bernhard Nieswandt, and Grzegorz Sumara
DOI: 10.15252/emmm.202216858

Corresponding author(s): Grzegorz Sumara (g.sumara@nencki.edu.pl) , David Stegner (stegner@virchow.uni-wuerzburg.de), Bernhard Nieswandt (bernhard.nieswandt@virchow.uni-wuerzburg.de)

Review Timeline:

Submission Date:	8th Sep 22
Editorial Decision:	4th Oct 22
Revision Received:	3rd Jun 23
Editorial Decision:	29th Jun 23
Revision Received:	4th Jul 23
Accepted:	7th Jul 23

Editor: Lise Roth

Transaction Report:

(Note: With the exception of the correction of typographical or spelling errors that could be a source of ambiguity, letters and reports are not edited. Depending on transfer agreements, referee reports obtained elsewhere may or may not be included in this compilation. Referee reports are anonymous unless the Referee chooses to sign their reports.)

4th Oct 2022

Dear Dr. Sumara,

Thank you for the submission of your manuscript to EMBO Molecular Medicine. We have now received feedback from the three reviewers who agreed to evaluate your manuscript. As you will see from the reports below, the reviewers raise substantial concerns on your work, which unfortunately preclude its publication in EMBO Molecular Medicine in its current form.

The reviewers find that the question addressed by the study is of potential interest, however they remain unconvinced that some of the major conclusions are sufficiently supported by the data. In particular, the manuscript should be re-written to be better structured, include more details on the methodology and better explain the rationale. The statistics should be revised, controls are missing, and the mechanistic insight should be deepened.

If you feel you can satisfactorily address these points and those listed by the referees, you may wish to submit a revised version of your manuscript. Please attach a covering letter giving details of the way in which you have handled each of the points raised by the referees. A revised manuscript will once again be subject to review and we cannot guarantee at this stage that the eventual outcome will be favorable. Given the extent of work required to address the referees' concerns, we are willing to extend the deadline for revisions to 6 months.

EMBO Molecular Medicine encourages a single round of revision only and therefore, acceptance or rejection of the manuscript will depend on the completeness of your responses included in the next, final version of the manuscript. For this reason, and to save you from any frustrations in the end, I would strongly advise against returning an incomplete revision.

When submitting your revised manuscript, please carefully review the instructions that follow below. We perform an initial quality control of all revised manuscripts before re-review; failure to include requested items will delay the evaluation of your revision.

We require:

1) A .docx formatted version of the manuscript text (including legends for main figures, EV figures and tables). Please make sure that the changes are highlighted to be clearly visible.

2) Individual production quality figure files as .eps, .tif, .jpg (one file per figure). For guidance, download the 'Figure Guide PDF' (https://www.embopress.org/page/journal/17574684/authorguide#figureformat).

3) A .docx formatted letter INCLUDING the reviewers' reports and your detailed point-by-point responses to their comments. As part of the EMBO Press transparent editorial process, the point-by-point response is part of the Review Process File (RPF), which will be published alongside your paper.

4) A complete author checklist, which you can download from our author guidelines (https://www.embopress.org/page/journal/17574684/authorguide#submissionofrevisions). Please insert information in the checklist that is also reflected in the manuscript. The completed author checklist will also be part of the RPF.

5) Please note that all corresponding authors are required to supply an ORCID ID for their name upon submission of a revised manuscript. An ORCID identified is currently missing for B. Nieswandt.

6) It is mandatory to include a 'Data Availability' section after the Materials and Methods. Before submitting your revision, primary datasets produced in this study need to be deposited in an appropriate public database, and the accession numbers and database listed under 'Data Availability'. Please remember to provide a reviewer password if the datasets are not yet public (see https://www.embopress.org/page/journal/17574684/authorguide#dataavailability).
In case you have no data that requires deposition in a public database, please state so in this section. Note that the Data Availability Section is restricted to new primary data that are part of this study.

7) For data quantification: please specify the name of the statistical test used to generate error bars and P values, the number (n) of independent experiments (specify technical or biological replicates) underlying each data point and the test used to calculate p-values in each figure legend. The figure legends should contain a basic description of n, P and the test applied. Graphs must include a description of the bars and the error bars (s.d., s.e.m.). Please provide exact p values.

8) We would also encourage you to include the source data for figure panels that show essential data. Numerical data should be provided as individual .xls or .csv files (including a tab describing the data). For blots or microscopy, uncropped images should be submitted (using a zip archive if multiple images need to be supplied for one panel). Additional information on source data and instruction on how to label the files are available at

.

9) Our journal encourages inclusion of *data citations in the reference list* to directly cite datasets that were re-used and obtained from public databases. Data citations in the article text are distinct from normal bibliographical citations and should directly link to the database records from which the data can be accessed. In the main text, data citations are formatted as follows: "Data ref: Smith et al, 2001" or "Data ref: NCBI Sequence Read Archive PRJNA342805, 2017". In the Reference list, data citations must be labeled with "[DATASET]". A data reference must provide the database name, accession number/identifiers and a resolvable link to the landing page from which the data can be accessed at the end of the reference. Further instructions are available at .

10) We replaced Supplementary Information with Expanded View (EV) Figures and Tables that are collapsible/expandable online. A maximum of 5 EV Figures can be typeset. EV Figures should be cited as 'Figure EV1, Figure EV2" etc... in the text and their respective legends should be included in the main text after the legends of regular figures.
- For the figures that you do NOT wish to display as Expanded View figures, they should be bundled together with their legends in a single PDF file called *Appendix*, which should start with a short Table of Content. Appendix figures should be referred to in the main text as: "Appendix Figure S1, Appendix Figure S2" etc.
- Additional Tables/Datasets should be labeled and referred to as Table EV1, Dataset EV1, etc. Legends have to be provided in a separate tab in case of .xls files. Alternatively, the legend can be supplied as a separate text file (README) and zipped together with the Table/Dataset file.
See detailed instructions here:
.

11) The paper explained: EMBO Molecular Medicine articles are accompanied by a summary of the articles to emphasize the major findings in the paper and their medical implications for the non-specialist reader. Please provide a draft summary of your article highlighting
- the medical issue you are addressing,
- the results obtained and
- their clinical impact.
This may be edited to ensure that readers understand the significance and context of the research. Please refer to any of our published articles for an example.

12) For more information: There is space at the end of each article to list relevant web links for further consultation by our readers. Could you identify some relevant ones and provide such information as well? Some examples are patient associations, relevant databases, OMIM/proteins/genes links, author's websites, etc...

13) Author contributions: CRediT has replaced the traditional author contributions section because it offers a systematic machine readable author contributions format that allows for more effective research assessment. Please remove the Authors Contributions from the manuscript and use the free text boxes beneath each contributing author's name in our system to add specific details on the author's contribution. More information is available in our guide to authors.

14) Conflict of interest: We updated our journal's competing interests policy in January 2022 and request authors to consider both actual and perceived competing interests. Please review the policy https://www.embopress.org/competing-interests and update your competing interests if necessary.

15) Every published paper now includes a 'Synopsis' to further enhance discoverability. Synopses are displayed on the journal webpage and are freely accessible to all readers. They include a short stand first (maximum of 300 characters, including space) as well as 2-5 one-sentences bullet points that summarizes the paper. Please write the bullet points to summarize the key NEW findings. They should be designed to be complementary to the abstract - i.e. not repeat the same text. We encourage inclusion of key acronyms and quantitative information (maximum of 30 words / bullet point). Please use the passive voice. Please attach these in a separate file or send them by email, we will incorporate them accordingly.
Please also suggest a striking image or visual abstract to illustrate your article as a PNG file 550 px wide x 300-600 px high.

16) As part of the EMBO Publications transparent editorial process initiative (see our Editorial at http://embomolmed.embopress.org/content/2/9/329), EMBO Molecular Medicine will publish online a Review Process File (RPF) to accompany accepted manuscripts.
In the event of acceptance, this file will be published in conjunction with your paper and will include the anonymous referee reports, your point-by-point response and all pertinent correspondence relating to the manuscript. Let us know whether you agree with the publication of the RPF and as here, if you want to remove or not any figures from it prior to publication.
Please note that the Authors checklist will be published at the end of the RPF.

EMBO Molecular Medicine has a "scooping protection" policy, whereby similar findings that are published by others during review or revision are not a criterion for rejection. Should you decide to submit a revised version, I do ask that you get in touch after three months if you have not completed it, to update us on the status.

I look forward to receiving your revised manuscript.

Yours sincerely,

Lise Roth

Lise Roth, PhD
Senior Editor
EMBO Molecular Medicine

***** Reviewer's comments *****

Referee #1 (Remarks for Author):

The study by Till Karwell and colleagues, entitled "Platelet-derived lipids promote insulin sercretion of pancreatic ß cells" describes the key role of 20-HETE secreted from platelets to stimulate insulin release, both in vitro and in vivo. Using a combination of genetically-engineered mouse models and pharmacological treatments, the authors identify here that platelets are present in the pancreas, close to pancreatic islets, that they play a key role in regulating glucose homeostasis (glucose tolerance more than insulin sensitivity) through stimulation of insulin secretion in response to glucose and, finally, that the platelet effect on insulin secretion declines with age, due to a decrease of 20-HETE in aged mice. They demonstrate a platelet-20-HETE-FFAR1-PKD1 signaling pathway that contributes to modulate insulin secretion. The authors also mention that ß-cell dervived factor(s) may contribute to activate platelet within the pancreas.
This is an interesting story that shed light on a new mechanism that regulates insulin secretion and, more importantly, that can have important implications for defining new therapeutic strategies for type 2 diabetes.

Although this is a well-designed story, several points should be addressed before publication.

Since high glucose concentration plays a role in platelet activation, what is the effect of chronic hyperglycemia regarding platelet activity? Does long term high fat diet feeding modulate platelet location/activity (for example, in chow diet versus HFD fed C57Bl6J mice)? Does HFD exacerbate glucose dyshomeostasis in their different mouse models of platelet deficiency?

Why were Min6 cells cultured in Matrigel? Does this have an impact per se on platelet activity?

In static GSIS experiments, acute treatment of ß cells with human or mouse platelets were performed. Does a longer treatment (16 hours for example) have the same effect on stimulating insulin secretion?

The experiment with HET0016 is convincing. Since Tunaru et al have previously demonstrated that 20-HETE may originate form ß cells, it might be interesting to verify that treating mouse isolated islets with HET0016 does not modify platelet-mediated increase of GSIS.

Minor :

At the end of the introduction section, it is mentioned that "20-HETE decline with age leading to the amelioration of platelets's action on ß-cells". This should be reformulated since the authors show that 20-HETE exerts beneficial effect on insulin secretion.

Referee #2 (Remarks for Author):

The manuscript by Karwen et al describes how platelets regulate insulin secretion by pancreatic beta cells through the production of a specific lipid. This interesting study shows inter-organ regulation and identify the molecular link between platelets and beta cells through a specific lipid that regulates insulin secretion. Complementary experiments, performed in vitro and in vivo, led to convincing results. The use of such lipids may represent new therapeutical avenues. Yet, several modifications are needed before the manuscript can be considered suitable for publication.

All along the manuscript, "metabolic homeostasis" should be replaced by "energy homeostasis". Metabolic homeostasis refers to much more molecules than just nutrients.
All along the manuscript, "beta cells" should be used instead of "beta-cells", except when it is used as an adjective. Same applies for alpha cells.
Page 3, last sentence, it refers to American Diabetes 2013 but the correct reference should be the journal, not the association.
Last sentence of the introduction, the authors mention that 20 HETE increases insulin secretion and that its levels are decreased with age. One would thus expect a decreased insulin secretion with age but the next sentence says that decreasing levels of 20 HETE leads to amelioration of platelet's action on beta cells. Please correct.
In the results, the whole description of the experiments shown on supplementary figure 1 is too short. Please describe panels one by one. Moreover, it is unusual to start with a supplementary figure.
Title of supp fig 1 is not correct. Glucose is involved but no evidence that humoral factors are required.
Is the medium used to grow min6 cells similar to medium used to grow 3T3L1 or Hek ? Please precise the media and concentration of glucose and serum. It is important when comparing platelet adhesion.
Please describe how motile and adherent platelet can be distinguished.
What is the use of cd105 ? GPIX ?
Page 7 "Platelets were detected more frequently at the endothelium of the endocrine than the exocrine pancreas (Figure 1, G and H)". Please show absence of colocalization with exocrine markers (such as amylase).
I am not sure I understood correctly the aim of the experiment presented on sup fig 2D. Treating mice with R300 antibody removes platelet from the whole body. Thus, it also logical that platelet number decrease in islets.
The conclusion of the experiment shown on sup fig 2 is "Thus, high glucose triggers the release of a factor from β-cells that locally stimulates platelets to interact with endothelium in the pancreatic islets". If this is to be shown, then why not studying platelets in islets of hyperglycemic mice ?
ITT curves on figure 2F are different from curves from panel C, when comparing control mice. Please comment on this difference.
Concerning fig 3, "GPVI depletion by JAQ1-IgG or GPVI-blockade by JAQ1-F(ab)2, blockage of GPIIb/IIIa by JON/A-F(ab)2, or systemic platelet depletion using R300 antibody also resulted in glucose intolerance resulting from decreased insulin secretion in response to glucose, while insulin sensitivity was not affected (Figure 3, A-I).". How do you control for the efficiency of treatments? Counting platelets? It is required.
Figure 4A shows secretion at low glucose but it should also show insulin secretion in response to high glucose. Especially since in vivo experiments show that insulin secretion is altered not at low glucose but a high glucose (after glucose injection).
On fig4F, why showing only ins secretion in high glucose and not in low glucose as shown for other panels ?
What is the specific goal of showing results obtained with endocbetaH1 and endocbetaH5 ?
On fig5, panels B and C could be added to panel A, no need to separate them.
The description of fig5 panel D is confusing. The authors say that 4 types of lipids were considerably decreased. What is the criteria to select these 4 lipids ? Is there a threshold ? Why only 4 ?
On figure 6, the stimulation factor by HPS is not important. Moreover, basal insulin secretion is impaired by the Gsq inhibitor, even in the absence of HPS.
The conclusion from fig6A is not correct, the use of GW100 indicates that FFAR1 is involved in hPS stimulated beta cells but the conclusion about HETE is far fetched since other FFAR1 ligands exist.
Concerning fig 6B, the response to hPS is still present even when siRNA for FFAR1 is applied. The authors should acknowledge that blocking is not complete.
After HET0016 treatment in mice, mice display glucose intolerance. Please show both basal and glucose induced plasma insulin levels.
The legend of figure 5 is not correct, it only comments on siRNA experiments and not on in vivo experiments.
Since several forms of PKD are shown on figure 6F, a complete description of the figure is required. Without that, fig 6F cannot be understood.
The authors should document and comment about the regulation of HETE by pancreatic beta cells. Do they have any data about what stimulates or inhibits HETE secretion ?
The efficiency of clopidogrel on platelets should be measured to evaluate efficiency of treatment. And more importantly, this should absolutely evaluated in young and old mice since treatment in old mice seems to have no effect in old mice.
Concerning HETE levels in humans, why selecting patients less than 65 and more than 65? Results could be different if groups are less than 40 and more than 65.
Could HETE be measured in the secretome of platelets obtained from young versus old subjects?
Discussion should include dat about proteins present at the surface of islet endothelial cells that could be responsible for platelet activation. Are there specific proteins to islet endothelium versus other endothelium?
Page 30: "With aging, the responsiveness of pancreatic β-cells to glucose declines". Not all studies show that result. Nat Med 2016 by Ben Porath and Dor show that induced senescence is associated with increased insulin secretion PMID: 26950362. Please acknowledge the controversy.

Referee #3 (Comments on Novelty/Model System for Author):

Technical quality: methodology is not clearly defined or referenced in many methods. No information is provided about rat

studies - this includes the Study approval and dosage details. Statistical analysis also needs to be evaluated carefully.
Novelty: adequate.
Medical impact: limited due to a small sub group of human studies and lack of mechanistic insights/therapeutic targets.
Adequacy of model system: difference transgenic strains are used ; however the significance of each is not clearly defined. Why the authors have chosen one model over another for certain experiments is also not evident. It is also not specified why only male mice were used in the study.

Referee #3 (Remarks for Author):

Platelet-derived lipids promote insulin secretion of pancreatic β-cells
EMM-2022-16858
The manuscript by Karwan et al. describes the effect of platelet depletion or loss of platelet functional pathways on insulin secretion The authors go on to describe platelet derived factors, with a focus on 20-HETE, as a major factor for regulating beta cell function and insulin secretion. The study focusses primarily on insulin secretion and glucose intolerance studies as markers of glucose metabolism; this provides some interesting findings using a combination of in vitro, in vivo and ex vivo studies. However, it lack in-depth mechanistic insights and presents major concerns with experimental design.

1. How does the level of soluble platelet activation markers such as P-selectin corelate with the insulin and circulating glucose levels?
2. Are there any differences observed in the Insulin receptor and/or insulin-like growth factor-1 receptor signaling which play a key role in responding to fluctuations in glucose and insulin levels?
3. Does incubation of GPIbα KO mice platelets with insulin lead to altered platelet aggregation?
4. The authors use varying concentrations of high glucose (25mM, 20 mM and 15mM) in the study (Fig1 and Fig 4) based on the cell line/samples used- the rationale for choosing these concentrations in the different experiments should be clearly indicated in the results section.
5. In the initial studies (Suppl Fig 1) for confirming the platelet activation with high glucose levels, the authors should also include results from incubation of the mouse and human platelets with 5mM glucose (normoglycemic) as an internal control to identify the effect in comparison with more physiological conditions.
6. The authors show that deletion of extracellular domain of GPIbα results in glucose intolerance; other additional markers such as C-peptide and glucose transporters should be evaluated in the mice samples.
7. The authors have chosen to use only male mice (and rats???) in their study? Why is this? This should be clearly stated in the manuscript & figure legends and also included as a limitation. Since, female mice have been shown to be less prone to insulin resistance and diabetes than male mice, the authors should include data to address whether there were any differences between male and female mice (glucose intolerance, insulin secretion , etc) in their models? Was there any difference in platelet counts and mean platelet volume?
8. For the studies using pharmacological inhibition of platelet function or platelet depletion, was there a change in expression of glucose transporters like Glut1 and Glut 3 in platelets.
9. Does inhibition of 20-HETE release from platelets (using in vivo studies in wild type mice) alter insulin secretion and/or glucose tolerance? Are there morphological changes observed in beta cells?
10. For studies on 'aging' (Figure 7), the authors use 61 week, 63 week,70 week and 74 week old mice ; ages of young mice used also vary from 8 weeks to 13 weeks. As was shown by Jain et al 2019 (PMID: 31130473) using mice longitudinal studies, platelet phenotype differs with age in mice. With a variation of as much as 4-13 weeks in the mice age, what is rationale for choosing these different ages. This should be clearly addressed in the results section or included as a limitation.
11. Why was clopidogrel administered for 3 weeks for glucose tolerance test and 5 weeks for insulin secretion studies (Fig 7)? Similar concern for treatment of rats with clopidogrel with 8 weeks, 9 weeks and 10 weeks for glucose tolerance, insulin secretion and insulin tolerance respectively.
12. Methods section: No information provided about rat studies (Supplemental Figure 6, B-D etc)
13. Statistical concerns:
(a) For sample sizes 6, non-parametric Mann Whitney test would be more appropriate than t-test as normal distribution cannot be assumed for small cohorts. Non parametric (ex. Kruskal Wallis) tests with appropriate multiple comparisons tests should also be used for more than two groups. Normality tests should be clearly indicated
(b) It is not clear why authors chose to represent the data as Mean SD in some figure panels and as Mean SEM in others. Please provide a rationale for the same in the statistical analysis section.
14. The figure legends should include more details about the experimental design and treatments. For most figures, it is difficult to ascertain the methodology/experimental setup used for the results shown.
15. Overall, the manuscript should overall be rewritten in a more organized manner to clearly convey the findings while also providing sufficient details for better understanding.

Dear Dr. Roth,

Thank you very much for all your efforts to improve our manuscript.

Enclosed you can find a revised version of our paper and a detailed response to all the comments of the reviewers. In our opinion, we addressed all the concerns of all three reviewers. Especially, we tested the impact of platelets on glucose tolerance in mouse models of type 2 diabetes and verified their impact on glucose homeostasis in female mice. We also clarified all the technical issues raised by reviewers including concerns regarding statistical analyzes raised by reviewer #3. Finally, we included all experiments to verify the efficiency of pharmacological mouse models used in our study. We feel that addressing all of the comments allowed us to greatly improve our manuscript.

If you would have any questions, please do not hesitate to contact me directly.

Sincerely Yours,
Grzegorz Sumara

Response to the reviewers comments:

Referee #1 (Remarks for Author):

"The study by Till Karwell and colleagues, entitled "Platelet-derived lipids promote insulin sercretion of pancreatic ß cells" describes the key role of 20-HETE secreted from platelets to stimulate insulin release, both in vitro and in vivo. Using a combination of genetically-engineered mouse models and pharmacological treatments, the authors identify here that platelets are present in the pancreas, close to pancreatic islets, that they play a key role in regulating glucose homeostasis (glucose tolerance more than insulin sensitivity) through stimulation of insulin secretion in response to glucose and, finally, that the platelet effect on insulin secretion declines with age, due to a decrease of 20-HETE in aged mice. They demonstrate a platelet-20-HETE-FFAR1-PKD1 signaling pathway that contributes to modulate insulin secretion. The authors also mention that ß-cell dervived factor(s) may contribute to activate platelet within the pancreas.
This is an interesting story that shed light on a new mechanism that regulates insulin secretion and, more importantly, that can have important implications for defining new therapeutic strategies for type 2 diabetes.
Although this is a well-designed story, several points should be addressed before publication."

We thank reviewer #1, for his/her constructive and helpful comments on our manuscript

"Since high glucose concentration plays a role in platelet activation, what is the effect of chronic hyperglycemia regarding platelet activity? Does long term high fat diet feeding modulate platelet location/activity (for example, in chow diet versus HFD fed C57Bl6J mice)?

Does HFD exacerbate glucose dyshomeostasis in their different mouse models of platelet deficiency?"

This is a very important point raised by reviewer #1. Our initial data indicated glucose stimulates platelet reactivity, at least *in vitro* (Current Figure 1 and Figure EV1 B-D). Following the reviewer's suggestion, we compared platelets from mice fed for 12 weeks with a high-fat diet (HFD) to induce hyperglycemia with age-matched mice presenting normal glucose tolerance (normal diet – ND). Platelets from mice fed HFD did not differ in size, number, or expression of surface glycoproteins, or reactivity compared to mice fed the chow diet (Appendix Figure S1 C-H). This might be caused by the fact that increased hyperlipidemia and hormonal imbalance present in animals fed HFD might mask the effect of hyperglycemia alone. Decreased platelet reactivity in a whole-blood might not be relevant for pancreatic β cell physiology. For these reasons, we also compared the number of attached platelets in the pancreatic islets of mice fed ND and HFD. Histological analyses revealed no significant changes in the attachment of the platelets to the endothelium of the pancreatic islets between normal and high-fat diet (HFD) fed animals (Appendix Figure S1 I and J).

In parallel, we investigated the impact of HFD feeding on glucose tolerance in mice presenting defective platelets activation. Platelet depletion in mice fed HFD resulted in a slight but significant reduction of glucose tolerance (Figure EV5 H). However, the administration of clopidogrel in mice fed HFD did not alter glucose tolerance (Figure EV5 I). Similarly, HFD-feeding ameliorated the effect on glucose tolerance observed in mice carrying a platelet-restricted deletion of Gαq and Gα13 proteins (Figure EV5 J).

"Why were Min6 cells cultured in Matrigel? Does this have an impact per se on platelet activity?"

We used Matrigel-coated plastic to culture all the cell types used in the experiments presented in Figure 1A-D (current Figure 2A-D). In these experiments, we also used it as a control medium incubated on Matrigel-coated plastic but without cells. We used Matrigel in our experiments to prevent the detachment of cells. Therefore, we assume that the effects of the specific cell types on platelet reactivity are caused by the substances secreted by presented cells. This is explained in the revised manuscript.

"In static GSIS experiments, acute treatment of ß cells with human or mouse platelets were performed. Does a longer treatment (16 hours for example) have the same effect on stimulating insulin secretion?"

We performed the experiment suggested by reviewer #1, we observed that PS effectively induce insulin secretion during 2h and 8h, but not 16h stimulation (Figure EV4 H).

"The experiment with HET0016 is convincing. Since Tunaru et al have previously demonstrated that 20-HETE may originate form ß cells, it might be interesting to verify that treating mouse isolated islets with HET0016 does not modify platelet-mediated increase of GSIS."

We thank this reviewer for proposing this experiment. To address the question we utilized pancreatic β cell line INS1. Following the preincubation of INS1 cells with a HET0016 inhibitor, and their stimulation with supernatants from activated platelets we measured insulin concentration in the medium. Pre-treatment of INS1 cells with HET0016 did not abolish the

effect of hPS on insulin secretion. Moreover, HET0016 also did not affect insulin secretion when cells were treated with a control buffer (Figure 7 E).

“Minor :

At the end of the introduction section, it is mentioned that "20-HETE decline with age leading to the amelioration of platelets's action on ß-cells". This should be reformulated since the authors show that 20-HETE exerts beneficial effect on insulin secretion.”

We corrected this sentence.

Referee #2 (Remarks for Author):

“The manuscript by Karwen et al describes how platelets regulate insulin secretion by pancreatic beta cells through the production of a specific lipid. This interesting study shows inter-organ regulation and identify the molecular link between platelets and beta cells through a specific lipid that regulates insulin secretion. Complementary experiments, performed in vitro and in vivo, led to convincing results. The use of such lipids may represent new therapeutical avenues. Yet, several modifications are needed before the manuscript can be considered suitable for publication.”

We were pleased to see that this Reviewer found the data ‘convincing’ and thank him/her for the helpful and constructive comments that help to improve the manuscript.

“All along the manuscript, "metabolic homeostasis" should be replaced by "energy homeostasis". Metabolic homeostasis refers to much more molecules than just nutrients.”

“All along the manuscript, "beta cells" should be used instead of "beta-cells", except when it is used as an adjective. Same applies for alpha cells.”

“Page 3, last sentence, it refers to American Diabetes 2013 but the correct reference should be the journal, not the association.”

“Last sentence of the introduction, the authors mention that 20 HETE increases insulin secretion and that its levels are decreased with age. One would thus expect a decreased insulin secretion with age but the next sentence says that decreasing levels of 20 HETE leads to amelioration of platelet's action on beta cells. Please correct.”

We corrected the text according to the Reviewer’s suggestions.

“In the results, the whole description of the experiments shown on supplementary figure 1 is too short. Please describe panels one by one. Moreover, it is unusual to start with a supplementary figure.”

We moved panels A, B, C, and D from Supplementary Figure 1 to Figure 1. We have also extended the description of experiments.

"Title of supp fig 1 is not correct. Glucose is involved but no evidence that humoral factors are required."

We modified the title of Supplementary Figure 1 (Current Figure EV1), it now reads "Glucose promotes platelet activity".

"Is the medium used to grow min6 cells similar to medium used to grow 3T3L1 or Hek ? Please precise the media and concentration of glucose and serum. It is important when comparing platelet adhesion."

The conditional medium (KRB) for all the cell types used in experiments presented on Figure 1A-D (Current Figure 2 A-D) was the same. This is now clarified in the relevant section of the Materials & Methods and on legends corresponding to Figure 2.

"Please describe how motile and adherent platelet can be distinguished. What is the use of cd105 ? GPIX ?"

We apologize for being rather brief on this point in the initial method section. We performed a series of 3D multicolor image stack series that allowed us to discriminate between adherent platelets (= present in at least 3 consecutive stacks) and motile platelets (= not stationary on consecutive stacks). When comparing the two platelet populations we realized that adherent platelets appeared bigger than motile platelets. One reason was the fact that adherent platelets appeared to be activated and to some extent spread on the blood vessel. Another reason was the under-sampling of motile platelets as their speed (= the blood flow) was faster than the scan rate of the microscope during image acquisition. The consequence of the size difference between the two platelet species was that a size filter of 8 μm^3 was the most convenient way to discriminate between both platelet populations. We have expanded our corresponding method section a bit to make this approach clearer. We used an anti-CD105 antibody to lebel blood vessel walls, and an anti-GPIX antibody was used to identify platelets. In the new version of the manuscript, we described it in the Materials & Methods. We also modified the text describing discrimination between motile and adhered platelets (please see a new paragraph in Materials & Methods).

"Page 7 "Platelets were detected more frequently at the endothelium of the endocrine than the exocrine pancreas (Figure 1, G and H)". Please show absence of colocalization with exocrine markers (such as amylase)."

We performed an experiment suggested by this Reviewer. We stained for amylase and insulin (please see below). Unfortunately, staining for amylase did not work together with staining for platelets but these experiments clearly indicate that this marker of the exocrine pancreas does not localize to the nuclear-dense areas of the pancreatic islets. In contrast, insulin localizes to the areas of the pancreas which are characterized by high nuclear density. Therefore, we assume that our localization of pancreatic islets is correct.

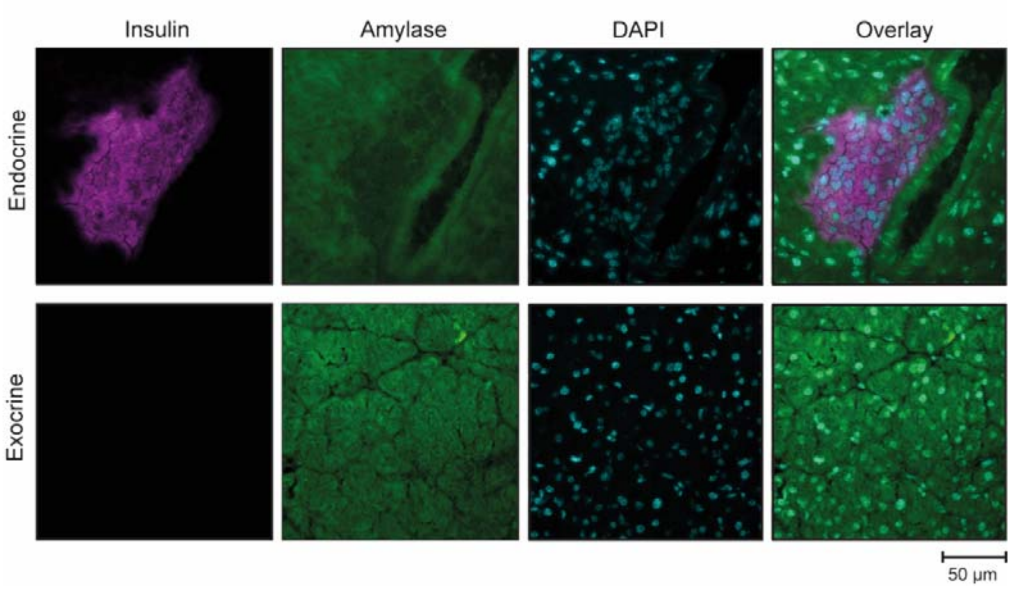

I am not sure I understood correctly the aim of the experiment presented on sup fig 2D. Treating mice with R300 antibody removes platelet from the whole body. Thus, it also logical that platelet number decrease in islets.

We agree that this is unclear and have tried to remedy this. The R300 antibody removes platelet from the whole body and therefore pancreas. We used this treatment to prove the specificity of our stainings. We made this clear in the current version of our manuscript.

The conclusion of the experiment shown on sup fig 2 is "Thus, high glucose triggers the release of a factor from β-cells that locally stimulates platelets to interact with endothelium in the pancreatic islets". If this is to be shown, then why not studying platelets in islets of hyperglycemic mice?

We modified this conclusion in the way that it refers to the results presented in current Figures 1 and 2:

"Taken together our data indicate that acute stimulation with glucose and yet unidentified factor derived from pancreatic β cells stimulates platelet reactivity. Moreover, our results suggest that a fraction of platelets pre-adhere to the endothelium of pancreatic islets."

We have also performed experiments on mice carrying defects of platelet activation or depleted from platelets and fed a high-fat diet (HFD), which induces hyperglycemia but also hyperlipidemia. The data are presented in current Figure EV5 H-J. Generally, HFD feeding largely ameliorated the effects of platelet dysfunction on pancreatic β cell function. This might be due to the prolonged protocol of HFD feeding, which results in the aging of mice. Alternatively, HFD-induced elevated levels of fatty acids in the circulation might result in the saturation of FFAR1 which might mask the effect of platelets-derived lipids.

"ITT curves on figure 2F are different from curves from panel C, when comparing control mice. Please comment on this difference."

As indicated in the legends for Figure 2 (current Figure 3), we used 0.25 U per kg of body weight of insulin for mice presented in the current Figure 3F and 0.5U per kg of body weight for animals presented in Figures 3 C and I. Thus the response to insulin is more pronounced in mice presented in Figuers 3C and 3I than in animals presented in Figure 3F.

Concerning fig 3, "GPVI depletion by JAQ1-IgG or GPVI-blockade by JAQ1-F(ab)2, blockage of GPIIb/IIIa by JON/A-F(ab)2, or systemic platelet depletion using R300 antibody also resulted in glucose intolerance resulting from decreased insulin secretion in response to glucose, while insulin sensitivity was not affected (Figure 3, A-I).". How do you control for the efficiency of treatments? Counting platelets? It is required.

We included these data in the new version of the manuscript. Briefly, depletion of the platelets using the R300 antibody caused more than a 95% reduction in platelet count. JAQ1 IgG almost completely depleted GPVI from the surface of the platelets but did not affect platelet count. Also, JON/A-F(ab)2 efficiently blocked GPIIb/IIIa, and JAQ1-F(ab)2 significantly blocked GPVI. Importantly, JAQ1-F(ab)2 did not affect the surface expression of GPIIb/IIIa, while JON/A-F(ab)2 did not alter the abundance of GPVI. All the data are now presented in Figure EV4 A-D.

"Figure 4A shows secretion at low glucose but it should also show insulin secretion in response to high glucose. Especially since in vivo experiments show that insulin secretion is altered not at low glucose but a high glucose (after glucose injection)."

In the new version of our manuscript, we included an experiment in which we co-culture INS1 cells with platelets under low and high glucose conditions. Importantly, under both conditions, platelets busted insulin secretion (Figure 5A).

"On fig4F, why showing only ins secretion in high glucose and not in low glucose as shown for other panels?"

We repeated this experiment including stimulation of human EndoC-βH5 with hPS in the presence of low and high glucose. This time, we also used a lower (more physiological) concentration of hPS. Importantly hPS increased insulin secretion in the presence of low and high glucose (Current Figure 5F).

What is the specific goal of showing results obtained with endocbetaH1 and endocbetaH5?

The purpose of these experiments was to extend the data in rodents to human beta cells. This is important as it is now quite clear that important differences exist between rodents and human islets. This was not stated. EndoC-βH5 is a newly established model of human β cells (Frorup *et al*, 2022). It was designed to overcome some functional limitations of EndoC-βH1 cell line which include low insulin production and secretory capacity, a tumorigenic phenotype, as well as chronic infection with a xenotropic murine virus (Frorup *et al.*, 2022; Kirkegaard *et al*, 2016; Tsonkova *et al*, 2018). However, EndoC-βH5 are not been fully characterized so far (Frorup *et al.*, 2022). Therefore, we decided to use in the study both cell lines.

"On fig5, panels B and C could be added to panel A, no need to separate them. The description of fig5 panel D is confusing. The authors say that 4 types of lipids were considerably decreased. What is the criteria to select these 4 lipids? Is there a threshold ? Why only 4 ?"

Results presented in Figure 5 A-C (current Figure 6A-C), were performed at different times and in slightly modified buffers. For treatment with monoaminoxidase A (MOA), to avoid the effect of this enzyme on serotonin produced by INS1 cells, proteins (including an added enzyme) were removed from human platelet supernatant (hPS) and control buffer by fractionation on columns. To compare the effect of MOA treatment control buffer and hPS fractionated on columns without enzyme treatment were used as a control. In the case of supernatants without lipid fraction, lipids were fractioned out from the control buffer and hPS by organic solvents. Therefore, we present these results on separate panels. For the selection of four lipid species, we used criteria of statistical significance. Only four lipids were significantly decreased in the serum of mice depleted from platelets, however after incorporating statistical analysis suggested by Reviewer #3 (Mann Whitney test instead of unpaired t-test) only HETEs were significantly decreased. We indicated this in the text and the figure.

"On figure 6, the stimulation factor by HPS is not important. Moreover, basal insulin secretion is impaired by the Gsq inhibitor, even in the absence of HPS. The conclusion from fig6A is not correct, the use of GW100 indicates that FFAR1 is involved in hPS stimulated beta cells but the conclusion about HETE is far fetched since other FFAR1 ligands exist."

We modified the text according to this Reviewer's comments.

"Concerning fig 6B, the response to hPS is still present even when siRNA for FFAR1 is applied. The authors should acknowledge that blocking is not complete."

We thank the Reviewer for this comment and have amended the manuscript accordingly

"After HET0016 treatment in mice, mice display glucose intolerance. Please show both basal and glucose induced plasma insulin levels."

We measured insulin production at basal and glucose-stimulated conditions in mice treated with HET0016 and control animals (Appendix Figure S2 C).

"The legend of figure 5 is not correct, it only comments on siRNA experiments and not on in vivo experiments."

Since no siRNA-based experiments were presented in Figure 5, we assumed that reviewer #2 means Supplementary Figure 5 (current Appendix Figure S2). Indeed its title was not correct and has been modified.

"Since several forms of PKD are shown on figure 6F, a complete description of the figure is required. Without that, fig 6F cannot be understood."

We agree with reviewer #2. In the current version of the manuscript, we included a detailed description of Figure 6F (current Figure 7H).

"The authors should document and comment about the regulation of HETE by pancreatic beta cells. Do they have any data about what stimulates or inhibits HETE secretion ?"

According to the suggestion of the reviewer, we tested if the stimulation of platelets with high glucose influences 20-HETE secretion. In fact, high glucose levels increase 20-HETE release from platelets upon stimulation with collagen related protein (Figure 6G).

"The efficiency of clopidogrel on platelets should be measured to evaluate efficiency of treatment. And more importantly, this should absolutely evaluated in young and old mice since treatment in old mice seems to have no effect in old mice."

Treatment of young adult and aged mice with clopidogrel was equally efficient in the reduction of P-selectin surface exposure and integrin activation in response to different platelet agonists (Figure EV5 G).

"Concerning HETE levels in humans, why selecting patients less than 65 and more than 65? Results could be different if groups are less than 40 and more than 65."

We re-analyzed our data according to reviewer #2 suggestion. The conclusions remain the same.

Could HETE be measured in the secretome of platelets obtained from young versus old subjects?

Our new data indicate that 20-HETE is markedly reduced in the secretome of platelets isolated from old compared to young mice (Figure 8G).

Discussion should include data about proteins present at the surface of islet endothelial cells that could be responsible for platelet activation. Are there specific proteins to islet endothelium versus other endothelium?

We thank the Reviewer for this suggestion. Little is known about specific features of endothelial cells in the endocrine pancreas. To underline this fact we included in the discussion the following paragraph:

Also, the specific organization of islets vasculature might determine the abundance of the platelets in pancreatic islets. Vascular density is approximately ten times higher in the endo- versus exocrine pancreas, the same applies to the density of fenestration. This correlates with a larger blood supply to the endocrine pancreas which receives about 15% of the total blood volume pumped to the pancreas, even though islets constitute 1-2% of the total mass of the pancreas (Hogan & Hull, 2017). Of note, the dysfunction of islets endothelium contributes to β cell failure during the development of diabetes (Hogan & Hull, 2017). The specific composition of the vasculature in the pancreatic islets suggests that endothelial cells from the endocrine pancreas possess special features (manifested also at the molecular level) distinct from the exocrine part of the pancreas and perhaps other tissues. However, despite clear morphological differences between endothelial cells from the endo- and exocrine pancreas little is known about molecular features that distinguish these populations of cells. Endothelial cells at different anatomical locations express many proteins that can modulate the adhesion

of platelets. This includes thrombomodulin (CD141), von Willebrand factor (vWF), coagulation factor V, and tissue factor (TF) (Siegel-Axel & Gawaz, 2007). To our knowledge, the relative abundance of these proteins in the endothelium of the endo- and exocrine pancreas have not been investigated so far. Recently, transcriptional profiles of human endothelial cells from the endo- and exocrine pancreas were determined. More than 1200 genes were differentially expressed in these populations of cells. Several functional gen sets were enriched in endothelial cell populations isolated either from the endo- or exocrine pancreas (Jonsson *et al*, 2020). However, based on existing data is not possible to predict if differences between molecular signatures between endothelial cells from the endo- and exocrine pancreas contribute to platelets adhesion in the vasculature of islets.

"Page 30: "With aging, the responsiveness of pancreatic β-cells to glucose declines". Not all studies show that result. Nat Med 2016 by Ben Porath and Dor show that induced senescence is associated with increased insulin secretion PMID: 26950362. Please acknowledge the controversy."

We thank reviewer #2 to draw our attention to this publication. We discussed this issue in our manuscript.

Referee #3 (Comments on Novelty/Model System for Author):

"Technical quality: methodology is not clearly defined or referenced in many methods. No information is provided about rat studies - this includes the Study approval and dosage details. Statistical analysis also needs to be evaluated carefully."

We apologize for this omission in a previous version of our manuscript. In the current version of our manuscript, we included all the details about rat studies including information about ethical approval. We have also reviewed our statistical analyzes according to the suggestions of reviewer #3 (please see our response to the detailed question of reviewer #3).

"Novelty: adequate."

We thank reviewer #3 for his/her acknowledgment of the novelty of our findings.

"Medical impact: limited due to a small sub group of human studies and lack of mechanistic insights/therapeutic targets."

We agree with reviewer #3 that our study has no immediate medical impact. However, as acknowledged by reviewer #3 our study implicated for the first time platelets in the regulation of β cell function and glucose homeostasis. In the longer perspective, this might lead to the development of new therapeutic agents. In the current version of our manuscript, we discussed this issue in the relevant section.

Adequacy of model system: difference transgenic strains are used ; however the significance of each is not clearly defined. Why the authors have chosen one model over another for certain experiments is also not evident. It is also not specified why only male mice were used in the study.

To better define the meaning of the results obtained in each of the in vivo models, we summarized the major findings after their detailed technical description. The following sentences have been added to the text:

“These data indicated that chronic impairment in platelet adhesion results in glucose intolerance associated with declined insulin secretion.”

“These results indicate that activation of platelets is required for glucose-stimulated insulin secretion.”

“Therefore, short-term blockage of platelet adhesion or platelet ablation results in similar glucose intolerance to long-term ablation of platelet function.”

We also agree with reviewer #3 that our in vivo experiments have been performed only in males and we could not exclude the possibility that our conclusions apply only to one gender. Therefore, we repeated a key experiment (glucose tolerance test) in one genetic and two pharmacological mouse models of impairment of the platelet function or platelet loss. The results are presented in Figure EV3 C, Figure EV4 E, and Figure EV5 C.

Referee #3 (Remarks for Author):

“Platelet-derived lipids promote insulin secretion of pancreatic β-cells EMM-2022-16858
The manuscript by Karwan et al. describes the effect of platelet depletion or loss of platelet functional pathways on insulin secretion The authors go on to describe platelet derived factors, with a focus on 20-HETE, as a major factor for regulating beta cell function and insulin secretion. The study focusses primarily on insulin secretion and glucose intolerance studies as markers of glucose metabolism; this provides some interesting findings using a combination of in vitro, in vivo and ex vivo studies. However, it lack in-depth mechanistic insights and presents major concerns with experimental design.”

We thank this Reviewer for his/her interest in our findings. In our opinion, with the help of all the comments of reviewers we provided a better description of the underlining mechanisms, improved the experimental design at the relevant parts, and addressed all the other concerns of the reviewers.

“1. How does the level of soluble platelet activation markers such as P-selectin corelate with the insulin and circulating glucose levels?”

We measured exposure of P-selectin, integrins, and the levels of Platelet basic protein (PBP) in mice injected with glucose. In fact, glucose transiently primed the activation of platelets (Figure EV1 B-D).

“2. Are there any differences observed in the Insulin receptor and/or insulin-like growth factor-1 receptor signaling which play a key role in responding to fluctuations in glucose and insulin levels?”

Decreased insulin levels upon stimulation with glucose in mice depleted from platelets correlated with lower activity of AKT in skeletal muscles and perigonadal adipose tissue (Figure EV4 F and G).

"3. Does incubation of GPIbα KO mice platelets with insulin lead to altered platelet aggregation?"

Previous studies on the role of insulin in the activation of platelets do not give a clear answer about the mode of action of this hormone on platelets. While one study indicates that insulin inhibits the activation of platelets other results lead to the conclusion that insulin might rather stimulate platelet aggregation (Ferreira *et al*, 2004; Hu *et al*, 2002; Yngen *et al*, 2001). Our new data indicate that insulin does not promote platelet activation as assessed by the surface exposure of P-selectin and integrin activation (Appendix Figure S1 A and B). Studying GPIbα-mediated platelet activation is challenging, as the primary function of the receptor is platelet tethering, which can only be investigated under shear conditions. However, previous studies established also that the deletion of GPIbα in platelets leads to a severe defect in thrombus formation that goes even beyond platelet tethering but indicates a role of GPIbα in platelet incorporation in thrombi (Bergmeier *et al*, 2006). Thus, we consider it unlikely that insulin has a dramatic effect in GPIbα-mice, but we do agree with the reviewer that further investigations are required to precisely define the role of insulin in the regulation of platelet action and the role of GPIbα in this process. However, in our opinion, this is beyond the scope of this manuscript.

"4. The authors use varying concentrations of high glucose (25mM, 20 mM and 15mM) in the study (Fig1 and Fig 4) based on the cell line/samples used- the rationale for choosing these concentrations in the different experiments should be clearly indicated in the results section."

We apologize for this omission. In fact, in some of the figure legends, the exact concentration of glucose was missing. In the current version of the manuscript, we fixed this error. The differences between the levels of glucose used for stimulation or culture of different cell lines arose based on commonly used glucose levels for culture or stimulation of different cell lines (culture condition for HEK cell – 25 mM, 3T3L1 – 25 mM, stimulatory conditions for MIN6 cells 25mM glucose, stimulatory conditions for INS1 cells 15 mM glucose). We also indicated clearly in the relevant parts of Materials and Methods.

"5. In the initial studies (Suppl Fig 1) for confirming the platelet activation with high glucose levels, the authors should also include results from incubation of the mouse and human platelets with 5mM glucose (normoglycemic) as an internal control to identify the effect in comparison with more physiological conditions."

We agree with reviewer #3. Therefore, we repeated this experiment using a more physiological concentration of glucose (5mM). The conclusion remained the same (Figure 1A-F).

"6. The authors show that deletion of extracellular domain of GPIbα results in glucose intolerance; other additional markers such as C-peptide and glucose transporters should be evaluated in the mice samples."

In fact, using different mouse models of platelet dysfunction we showed that these cells promote insulin levels and glucose tolerance. To further confirm obtained results, we have

chosen mice Gαq and Gα13 proteins in the platelets and animals depleted from platelets using the R300 antibody. As predicted by this Reviewer, c-peptide levels were reduced in mice with the deletion of Gαq and Gα13 in platelets (Figure EV3 A and B). However, the levels of Glucose transporter 4 (Glut4), responsible for insulin-stimulated glucose transport in skeletal muscle and adipose tissue, were not affected in both of these organs by platelet depletion (Figure EV4 F and G). As mentioned above activation of insulin signaling in skeletal muscle and adipose tissue is impaired in the absence of platelets. Stimulation of adipose tissue or skeletal muscle with insulin promotes Glut4 translocation to the plasma membrane but does not affect total Glut4 levels. Therefore, our overall conclusion that platelets promote insulin secretion which activates the insulin-dependent signaling cascade to promote glucose uptake in the peripheral organs is in line with the obtained results.

"7. The authors have chosen to use only male mice (and rats???) in their study? Why is this? This should be clearly stated in the manuscript & figure legends and also included as a limitation. Since, female mice have been shown to be less prone to insulin resistance and diabetes than male mice, the authors should include data to address whether there were any differences between male and female mice (glucose intolerance, insulin secretion , etc) in their models? Was there any difference in platelet counts and mean platelet volume?"

Previous studies have shown that male mice have slightly higher platelet counts than female mice, but mean platelet volumes do not differ between both sexes (Aurbach *et al*, 2019). However, the differences in platelet count are so small, that it is very unlikely that they do affect glucose tolerance, as platelet counts had to drop below 20% of normal to affect hemostasis or thrombosis (Morowski *et al*, 2013). Nevertheless, as mentioned above repeated key in vivo experiments in females. The results are presented in Figure EV3 C, Figure EV4 E, and Figure EV5 C. We also agree with reviewer #3 that information about the gender of the studied animals was missing in the previous version of our manuscript. We included it now in the figure legends.

"8. For the studies using pharmacological inhibition of platelet function or platelet depletion, was there a change in expression of glucose transporters like Gut1 and Glut 3 in platelets."

Treatment of mice with clopidogrel did not affect Glut1 and Glu3 levels in platelets (Figure EV5 B).

"9. Does inhibition of 20-HETE release from platelets (using in vivo studies in wild type mice) alter insulin secretion and/or glucose tolerance? Are there morphological changes observed in beta cells?"

Treatment of mice with an inhibitor of 20-HETE production (HET0016) reduced glucose-stimulated insulin secretion but did not affect pancreatic islet morphology (Appendix Figure S2 C-E).

"10. For studies on 'aging' (Figure 7), the authors use 61 week, 63 week,70 week and 74 week old mice ; ages of young mice used also vary from 8 weeks to 13 weeks. As was shown by Jain et al 2019 (PMID: 31130473) using mice longitudinal studies, platelet phenotype differs with age in mice. With a variation of as much as 4-13 weeks in the mice age, what is rationale for choosing these different ages. This should be clearly addressed in the results section or included as a limitation."

We thank the Reviewer for drawing our attention to this interesting study. We included it in our discussion as this paper is relevant to our study. In fact, we considered in our study 8 to 13 weeks (2-3 months) old mice as young. In the publication mentioned by reviewer #3, 6 to 18 months old animals were analyzed. We did not find studies in which significant changes in platelet action would be observed between 2 and 3 months of age. According to the paper cited by this Reviewer, the changes in platelet action between 14 and 17 months in mice were indeed observed. However, we used for all our studies experimental mice with age-matched control animals. The differences in the age of animals between different experiments came from the fact that the same cohorts of mice have been subjected to multiple metabolic tests. We act according to the 3R principle recommended by all ethical committees in Europe and the United States of America. However, we agree that this information was missing in the previous version of our manuscript, we have included it in the revised version in the relevant figure legends.

11. Why was clopidogrel administered for 3 weeks for glucose tolerance test and 5 weeks for insulin secretion studies (Fig 7)? Similar concern for treatment of rats with clopidogrel with 8 weeks, 9 weeks and 10 weeks for glucose tolerance, insulin secretion and insulin tolerance respectively.

This was done for similar reasons as outlined above, we used the same cohorts of mice for different experiments. We have also included this information in the relevant figure legend.

"12. Methods section: No information provided about rat studies (Supplemental Figure 6, B-D etc)"

We included the information requested.

"13. Statistical concerns:

(a) For sample sizes≤6, non-parametric Mann Whitney test would be more appropriate than t-test as normal distribution cannot be assumed for small cohorts. Non parametric (ex. Kruskal Wallis) tests with appropriate multiple comparisons tests should also be used for more than two groups. Normality tests should be clearly indicated"

As the reviewer requested for $n \leq 6$ and when data had not normal distribution parametrical tests (t-test and One-way ANOVA) were replaced by non-parametrical tests (Mann Whitney test and Kruskal Wallis test followed by Mann Whitney test as post-hoc analysis with Benjamini-Hochberg correction for multiple comparisons). For $n \geq 7$ normality of the data distribution was checked by the Shapiro-Wilk normality test. p-values were corrected according to the new statistical analysis (detailed changes are presented in the figures). The type of used test is clearly indicated in the description of the figures. The statistical analysis section in Material&Methods was corrected. The overall outcome of experiments presented in the paper and conclusions were not changed upon changed statistical analysis.
New statistical analysis was applied to figure (numbering according to the old version of the manuscript): S1 C-E, 1A-F, S2D, 2A-I, S3A-H, 3A-I, 4B, 4D, 4F, S4A, 5A-C, 5E-F, S5A-B, 6A-E, 7A-E, S6A-D. These correspond to figures 1B, C, E, F; S1A; 2A-D, F; S2D; 3A-I; S4D-K; 4A-I; 5B, D, F; S5I; 6A-C, E, G; S6A-B; 7A-D, F; S7A, C, D-F. in the new version of the manuscript.

"(b) It is not clear why authors chose to represent the data as Mean ± SD in some figure panels and as Mean ± SEM in others. Please provide a rationale for the same in the statistical analysis section."

All of the data in the figure panels were changed for Mean ± SEM. Where it is possible individual values are presented on the graph.

"14. The figure legends should include more details about the experimental design and treatments. For most figures, it is difficult to ascertain the methodology/experimental setup used for the results shown."

We thank reviewer #3 for drawing our attention to this problem. We reviewed the figure legends throughout our manuscript. We added the details such as the number of replicates used in each of the experiments, information about the gender and age of animals used, as well as the concentration of substances used for different treatments.

"15. Overall, the manuscript should overall be rewritten in a more organized manner to clearly convey the findings while also providing sufficient details for better understanding."

We reorganized a large part of the manuscript according to the suggestion of all three reviewers. We thank all of the suggestions of reviewer #3 as it was helping us to improve our manuscript.

References:

Aurbach K, Spindler M, Haining EJ, Bender M, Pleines I (2019) Blood collection, platelet isolation and measurement of platelet count and size in mice—a practical guide. *Platelets* 30: 698-707

Bergmeier W, Piffath CL, Goerge T, Cifuni SM, Ruggeri ZM, Ware J, Wagner DD (2006) The role of platelet adhesion receptor GPIbα far exceeds that of its main ligand, von Willebrand factor, in arterial thrombosis. *Proceedings of the National Academy of Sciences* 103: 16900-16905

Ferreira IA, Eybrechts KL, Mocking AI, Kroner C, Akkerman J-WN (2004) IRS-1 mediates inhibition of Ca2+ mobilization by insulin via the inhibitory G-protein Gi. *Journal of Biological Chemistry* 279: 3254-3264

Frorup C, Gerwig R, Sondergaard Svane CA, Mendes Lopes de Melo J, Floyel T, Pociot F, Kaur S, Storling J (2022) Characterisation of the functional and transcriptomic effects of pro-inflammatory cytokines on human EndoC-βH5 beta cells. *bioRxiv*: 2022.2011. 2029.518315

Hogan MF, Hull RL (2017) The islet endothelial cell: a novel contributor to beta cell secretory dysfunction in diabetes. *Diabetologia* 60: 952-959

Hu H, Hjemdahl P, Li N (2002) Effects of insulin on platelet and leukocyte activity in whole blood. *Thrombosis Research* 107: 209-215

Jonsson A, Hedin A, Müller M, Skog O, Korsgren O (2020) Transcriptional profiles of human islet and exocrine endothelial cells in subjects with or without impaired glucose metabolism. *Scientific Reports* 10: 22315

Kirkegaard JS, Ravassard P, Ingvarsen S, Diedisheim M, Bricout-Neveu E, Grønborg M, Frogne T, Scharfmann R, Madsen OD, Rescan C (2016) Xenotropic retrovirus Bxv1 in human pancreatic β cell lines. *The Journal of Clinical Investigation* 126: 1109-1113

Morowski M, Vögtle T, Kraft P, Kleinschnitz C, Stoll G, Nieswandt B (2013) Only severe thrombocytopenia results in bleeding and defective thrombus formation in mice. *Blood, The Journal of the American Society of Hematology* 121: 4938-4947

Siegel-Axel DI, Gawaz M, 2007. Platelets and endothelial cells, Seminars in thrombosis and hemostasis. Copyright© 2007 by Thieme Medical Publishers, Inc., 333 Seventh Avenue, New …, pp. 128-135.

Tsonkova VG, Sand FW, Wolf XA, Grunnet LG, Ringgaard AK, Ingvorsen C, Winkel L, Kalisz M, Dalgaard K, Bruun C (2018) The EndoC-βH1 cell line is a valid model of human beta cells and applicable for screenings to identify novel drug target candidates. *Molecular metabolism* 8: 144-157

Yngen M, Li N, Hjemdahl P, Wallén NH (2001) Insulin enhances platelet activation in vitro. *Thrombosis research* 104: 85-91

29th Jun 2023

Dear Dr. Sumara,

Thank you for the submission of your revised manuscript to EMBO Molecular Medicine. We have now received the reports from the 3 referees who re-reviewed your manuscript. As you will see, they are supportive of publication, and we will therefore be able to accept your manuscript once the following points will be addressed:

1/ Referees' comments: please address the remaining minor concerns/questions from referee #3.

2/ Main manuscript text:
- Please address the queries from our data editors in the related "Data edited MS file", accept the previous changes and only keep in track changes any new modification.
- We note that you currently have together with you a total of 3 co-corresponding authors. Is that correct? Do you confirm equal contribution of these 3 people, able to take full responsibility for the paper and its content?
Please note that all corresponding authors are required to supply an ORCID ID for their name upon submission of a revised manuscript (an ORCID identifier is currently missing for B. Nieswandt).
- We scanned your text for similarities with previously published material and similarities were found (see screenshot attached). I could unfortunately not check the website/source ("storage.grenadine.co") and thus see whether this was from a conference or published manuscript. Could you please clarify?
- Materials and methods:
o Animal experiments: please specify the gender of the animals.
o Human samples: please add the full statement that the experiments conformed to the principles set out in the WMA Declaration of Helsinki and the Department of Health and Human Services Belmont Report.
o Please provide antibody dilutions.
o Statistics: please include a statement about inclusion/exclusion criteria and update the checklist accordingly.
- Please move the Data Availability section after the Materials and Methods.
- Acknowledgements: The funding information should match the information provided in the submission system: please include the recipients of projects A01, A07, B07, B06 and B08. Diabetes UK grant is deleted in manuscript but is still in the submission system
- Please remove the conflict-of-interest statement from the second page and include it after the acknowledgements with the heading "Disclosure statement and competing interests". Please review the policy https://www.embopress.org/competing-interests and update your competing interests if necessary.

3/ Figures:
- Table EV1 should be removed from the manuscript and uploaded as a separate file, with its legend.

4/ Thank you for providing the Source Data. Please provide a completed SD checklist.

5/ Please provide "The paper explained": EMBO Molecular Medicine articles are accompanied by a summary of the articles to emphasize the major findings in the paper and their medical implications for the non-specialist reader. Please provide a draft summary of your article highlighting
- the medical issue you are addressing,
- the results obtained and
- their clinical impact.
This may be edited to ensure that readers understand the significance and context of the research. Please refer to any of our published articles for an example.

5/ Synopsis:
- Thank you for providing a nice synopsis image. Please resize it to 550 px wide x 300-600 px high (the text should remain legible).
- Please also provide a synopsis text: Synopses are displayed on the journal webpage and are freely accessible to all readers. They include a short stand first (maximum of 300 characters, including space) as well as 2-5 one-sentences bullet points that summarizes the paper. They should be designed to be complementary to the abstract - i.e. not repeat the same text. We encourage inclusion of key acronyms and quantitative information (maximum of 30 words / bullet point). Please use the passive voice. Please attach these in a separate file or send them by email, we will incorporate them accordingly.

6/ As part of the EMBO Publications transparent editorial process initiative (see our Editorial at http://embomolmed.embopress.org/content/2/9/329), EMBO Molecular Medicine will publish online a Review Process File (RPF) to accompany accepted manuscripts.
This file will be published in conjunction with your paper and will include the anonymous referee reports, your point-by-point

response and all pertinent correspondence relating to the manuscript. Let us know whether you agree with the publication of the RPF and as here, if you want to remove or not any figures from it prior to publication.
Please note that the Authors checklist will be published at the end of the RPF.

I look forward to receiving your revised manuscript as soon as possible.

With kind regards,

Lise Roth

Lise Roth, PhD
Senior Editor
EMBO Molecular Medicine

***** Reviewer's comments *****

Referee #1 (Comments on Novelty/Model System for Author):

The revised version of the study by Till Karwell and colleagues, entitled "Platelet-derived lipids promote insulin sercretion of pancreatic ß cells" describes the key role of 20-HETE secreted from platelets to stimulate insulin release, both in vitro and in vivo. In this study, the authors have used several experimental strategies, from genetically-engineered mouse models to pharmacological treatments. The technical quality of these experiments is high, well controlled, and the model systems and statistical analysis used are adequate. The ethical issues are described in the material and method section.

The authors identify here that platelets play a key role in regulating glucose homeostasis and that the platelet effect on insulin secretion declines with age, due to a decrease of 20-HETE in aged mice, which is novel and important for the diabetes field with a potential medical impact.

Referee #1 (Remarks for Author):

The authors have replied to all my concerns, adding some additional key experiments that improve the quality of the manuscript.

Referee #2 (Remarks for Author):

All the issues raised have been addressed and the new version of the manuscript is clearly improved. The results are stronger. The manuscript can now be accepted for publication.

Referee #3 (Comments on Novelty/Model System for Author):

Statistical concerns have largely been addressed in the revised manuscript. The rationale for the use of Wistar rats in the study has still not been clearly defined.

Referee #3 (Remarks for Author):

The authors have made significant efforts in addressing the concerns raised by this Reviewer, effectively alleviating most of the key issues. The revised manuscript and response provide adequate clarification for several concerns, particularly in terms of statistical considerations. However, some minor points need to be addressed prior to acceptance in the view of this Reviewer:
1. In the first Result section, suitable references should be provided for "While 2.8mM glucose is commonly considered .... occurring in untreated diabetes". It is not sufficient to mention 'commonly considered'.
2. "Platelet function is altered in the course of type 1 and type 2 diabetes, indicating that hyperglycemia might directly influence platelet reactivity" : please provide appropriate references.
3. Clarification is needed regarding the dosage of glucose injected in rats for the glucose tolerance test (2g/kg body weight) and glucose-stimulated insulin secretion (3g/kg body weight) as stated in the Methods section. The authors should provide either specific references or a rationale justifying the chosen protocol.
4. It is still not clear from the revised manuscript, why the authors have chosen to study mice and rats in parallel for the glucose tolerance and clopidogrel experiments ("The impact of platelets on insulin secretion declines with age')?

5. To enhance clarity, authors can choose to include a statement such as, "glucose intolerance resulting from glucose depletion (etc.) is independent of gender," emphasizing the comparable characteristics exhibited by male and female mice. Such information would be pertinent for metabolic studies focusing on sex-specific differences; this may also be highlighted in the discussion.
6. The authors should ensure appropriate referencing for all drugs (e.g., YM-25489, HET0016, CRT 0066101) and interventions utilized in the study. In case the particular drug or experimental strategy has not been previously employed, the authors should provide optimization data or a rationale justifying the selected dosage.

Response to the comments from Referee #3 (in blue):

Referee #3 (Remarks for Author):

The authors have made significant efforts in addressing the concerns raised by this Reviewer, effectively alleviating most of the key issues. The revised manuscript and response provide adequate clarification for several concerns, particularly in terms of statistical considerations. However, some minor points need to be addressed prior to acceptance in the view of this Reviewer:

1. In the first Result section, suitable references should be provided for "While 2.8mM glucose is commonly considered .... occurring in untreated diabetes". It is not sufficient to mention 'commonly considered'.

Suitable references have been added to the manuscript.

2. "Platelet function is altered in the course of type 1 and type 2 diabetes, indicating that

hyperglycemia might directly influence platelet reactivity" : please provide appropriate references.

Suitable references have been added to the manuscript.

3. Clarification is needed regarding the dosage of glucose injected in rats for the glucose tolerance test (2g/kg body weight) and glucose-stimulated insulin secretion (3g/kg body weight) as stated in the Methods section. The authors should provide either specific references or a rationale justifying the chosen protocol.

As written in the Materials and Methods section dosage of glucose injected in rats for the glucose tolerance test and for glucose-stimulated insulin secretion was 2g/kg of body weight. The reference for the used protocol has been added to the manuscript.

4. It is still not clear from the revised manuscript, why the authors have chosen to study mice and rats in parallel for the glucose tolerance and clopidogrel experiments ("The impact of platelets on insulin secretion declines with age')?

We implemented a rat model of platelet dysfunction to confirm that observed phenomena are conserved across species. It is known that the metabolism of mice and rats differs. Suitable information has been added to the manuscript.

5. To enhance clarity, authors can choose to include a statement such as, "glucose intolerance resulting from glucose depletion (etc.) is independent of gender," emphasizing the comparable characteristics exhibited by male and female mice. Such information would be pertinent for metabolic studies focusing on sex-specific differences; this may also be highlighted in the discussion.

Suggested information has been added to the discussion section of the manuscript.

6. The authors should ensure appropriate referencing for all drugs (e.g., YM-25489, HET0016, CRT 0066101) and interventions utilized in the study. In case the particular drug or experimental strategy has not been previously employed, the authors should provide optimization data or a rationale justifying the selected dosage.

The requested information has been added to the manuscript.

7th Jul 2023

Dear Dr. Sumara,

Thank you for submitting your revised files. I am pleased to inform you that your manuscript is now accepted for publication in EMBO Molecular Medicine!

I included minor modifications to your Paper Explained and Synopsis, please let me know if you agree with the following or amend as you see fit. Once I get your feedback, I'll include them in the manuscript files which will be sent out to production.

The paper Explained:
Problem
Diabetic patients demonstrate increased platelet activity, but the mechanism underlying platelet hyperactivity during hyperglycemia is not fully explored. Moreover, the fact that elevated glucose levels impact platelet action indicates a potential link between platelet function and glucose metabolism.
Results
We show that platelet activity is directly regulated by glucose and pancreatic β cell-derived factors. Moreover, a fraction of platelets localizes to the vasculature of pancreatic islets. Our findings further indicate that platelets secrete lipid-based factors including 20-Hydroxyeicosatetraenoic acid (20-HETE) to stimulate insulin secretion. Finally, we demonstrated that platelets impact on β cells declines with age.
Impact
We identify a cross-talk between platelets and pancreatic β cells. Based on elucidation of new mechanisms, our results highlight a group of substances that might be targeted to improve pancreatic β cell function.

Synopsis:
Platelet exhibit hyperactivity during hyperglycemia in diabetic patients. In this study, an unexpected role for platelets in the regulation of insulin secretion from pancreatic β cells was identified.
- Platelet function was regulated by glucose and β cells-derived factor/s
- A fraction of platelets preferentially localized to the vasculature of pancreatic islets
- Platelets released lipid-based factors such as 20-Hydroxyeicosatetraenoic acid (20-HETE) to stimulate insulin secretion
- The impact of platelets on pancreatic β cells declined with age

We would like to remind you that as part of the EMBO Publications transparent editorial process initiative, EMBO Molecular Medicine will publish a Review Process File online to accompany accepted manuscripts. If you do NOT want the file to be published or would like to exclude figures, please immediately inform the editorial office via e-mail.

Please read below for additional IMPORTANT information regarding your article, its publication and the production process.

Congratulations on your interesting work!

With kind regards,

Lise Roth

Lise Roth, Ph.D
Senior Editor
EMBO Molecular Medicine

Follow us on Twitter @EmboMolMed
Sign up for eTOCs at embopress.org/alertsfeeds

*** *** *** IMPORTANT INFORMATION *** *** ***

SPEED OF PUBLICATION
The journal aims for rapid publication of papers, using using the advance online publication "Early View" to expedite the process: A properly copy-edited and formatted version will be published as "Early View" after the proofs have been corrected.

Please help the Editors and publisher avoid delays by providing e-mail address(es), telephone and fax numbers at which author(s) can be contacted.

Should you be planning a Press Release on your article, please get in contact with embomolmed@wiley.com as early as possible, in order to coordinate publication and release dates.

LICENSE AND PAYMENT:

All articles published in EMBO Molecular Medicine are fully open access: immediately and freely available to read, download and share.

EMBO Molecular Medicine charges an article processing charge (APC) to cover the publication costs. You, as the corresponding author for this manuscript, should have already received a quote with the article processing fee separately. Please let us know in case this quote has not been received.

Once your article is at Wiley for editorial production you will receive an email from Wiley's Author Services system, which will ask you to log in and will present you with the publication license form for completion. Within the same system the publication fee can be paid by credit card, an invoice, pro forma invoice or purchase order can be requested.

Payment of the publication charge and the signed Open Access Agreement form must be received before the article can be published online.

PROOFS

You will receive the proofs by e-mail approximately 2 weeks after all relevant files have been sent o our Production Office. Please return them within 48 hours and if there should be any problems, please contact the production office at embopressproduction@wiley.com.

Please inform us if there is likely to be any difficulty in reaching you at the above address at that time. Failure to meet our deadlines may result in a delay of publication.

All further communications concerning your paper proofs should quote reference number EMM-2022-16858-V3 and be directed to the production office at embopressproduction@wiley.com.

Corresponding Author Name: Grzegorz Sumara, Bernhard Nieswandt, David Stegner
Journal Submitted to: EMBO Molecular Medicine
Manuscript Number: EMM-2022-16858

USEFUL LINKS FOR COMPLETING THIS FORM
The EMBO Journal - Author Guidelines
EMBO Reports - Author Guidelines
Molecular Systems Biology - Author Guidelines
EMBO Molecular Medicine - Author Guidelines

Reporting Checklist for Life Science Articles (updated January
This checklist is adapted from Materials Design Analysis Reporting (MDAR) Checklist for Authors. MDAR establishes a minimum set of requirements in transparent reporting in the life sciences (see Statement of Task: 10.31222/osf.io/9sm4x). Please follow the journal's guidelines in preparing your manuscript.
Please note that a copy of this checklist will be published alongside your article.

Abridged guidelines for figures
1. Data
The data shown in figures should satisfy the following conditions:
- the data were obtained and processed according to the field's best practice and are presented to reflect the results of the experiments in an accurate and unbiased manner.
- ideally, figure panels should include only measurements that are directly comparable to each other and obtained with the same assay.
- plots include clearly labeled error bars for independent experiments and sample sizes. Unless justified, error bars should not be shown for technical
- if n<5, the individual data points from each experiment should be plotted. Any statistical test employed should be justified.
- Source Data should be included to report the data underlying figures according to the guidelines set out in the authorship guidelines on Data

2. Captions
Each figure caption should contain the following information, for each panel where they are relevant:
- a specification of the experimental system investigated (eg cell line, species name).
- the assay(s) and method(s) used to carry out the reported observations and measurements.
- an explicit mention of the biological and chemical entity(ies) that are being measured.
- an explicit mention of the biological and chemical entity(ies) that are altered/varied/perturbed in a controlled manner.
- the exact sample size (n) for each experimental group/condition, given as a number, not a range;
- a description of the sample collection allowing the reader to understand whether the samples represent technical or biological replicates (including how many animals, litters, cultures, etc.).
- a statement of how many times the experiment shown was independently replicated in the laboratory.
- definitions of statistical methods and measures:
  - common tests, such as t-test (please specify whether paired vs. unpaired), simple χ2 tests, Wilcoxon and Mann-Whitney tests, can be unambiguously identified by name only, but more complex techniques should be described in the methods section;
  - are tests one-sided or two-sided?
  - are there adjustments for multiple comparisons?
  - exact statistical test results, e.g., P values = x but not P values < x;
  - definition of 'center values' as median or average;
  - definition of error bars as s.d. or s.e.m.

Please complete ALL of the questions below.
Select "Not Applicable" only when the requested information is not relevant for your study.

Materials

Newly Created Materials	Information included in the manuscript?	In which section is the information available? (Reagents and Tools Table, Materials and Methods, Figures, Data Availability Section)
New materials and reagents need to be available; do any restrictions apply?	Not Applicable	

Antibodies	Information included in the manuscript?	In which section is the information available? (Reagents and Tools Table, Materials and Methods, Figures, Data Availability Section)
For **antibodies** provide the following information:<br>- Commercial antibodies: RRID (if possible) or supplier name, catalogue number and or/clone number<br>- Non-commercial: RRID or citation	Yes	Materials and Methods

DNA and RNA sequences	Information included in the manuscript?	In which section is the information available? (Reagents and Tools Table, Materials and Methods, Figures, Data Availability Section)
**Short novel DNA or RNA including primers, probes**: provide the sequences.	Yes	Materials and Methods

Cell materials	Information included in the manuscript?	In which section is the information available? (Reagents and Tools Table, Materials and Methods, Figures, Data Availability Section)
**Cell lines:** Provide species information, strain. Provide accession number in repository **OR** supplier name, catalog number, clone number, and/**OR** RRID.	Yes	Materials and Methods
**Primary cultures:** Provide species, strain, sex of origin, genetic modification status.	Yes	Materials and Methods
Report if the cell lines were recently **authenticated** (e.g., by STR profiling) and tested for mycoplasma contamination.	Yes	Materials and Methods

Experimental animals	Information included in the manuscript?	In which section is the information available? (Reagents and Tools Table, Materials and Methods, Figures, Data Availability Section)
**Laboratory animals or Model organisms:** Provide species, strain, sex, age, genetic modification status. Provide accession number in repository **OR** supplier name, catalog number, clone number, **OR** RRID.	Yes	Materials and Methods and Figure legend
**Animal observed in or captured from the field:** Provide species, sex, and age where possible.	Not Applicable	
Please detail **housing and husbandry conditions**.	Yes	Materials and Methods

Plants and microbes	Information included in the manuscript?	In which section is the information available? (Reagents and Tools Table, Materials and Methods, Figures, Data Availability Section)
**Plants:** provide species and strain, ecotype and cultivar where relevant, unique accession number if available, and source (including location for collected wild specimens).	Not Applicable	
**Microbes:** provide species and strain, unique accession number if available, and source.	Not Applicable	

Human research participants	Information included in the manuscript?	In which section is the information available? (Reagents and Tools Table, Materials and Methods, Figures, Data Availability Section)
If collected and within the bounds of privacy constraints report on age, sex and gender or ethnicity for all study participants.	Yes	Materials and Methods

Core facilities	Information included in the manuscript?	In which section is the information available? (Reagents and Tools Table, Materials and Methods, Figures, Data Availability Section)
If your work benefited from core facilities, was their service mentioned in the acknowledgments section?	Not Applicable	

Design

Study protocol	Information included in the manuscript?	In which section is the information available? (Reagents and Tools Table, Materials and Methods, Figures, Data Availability Section)

If study protocol has been **pre-registered, provide DOI in the manuscript**. For clinical trials, provide the trial registration number **OR** cite DOI.	Not Applicable	
Report the **clinical trial registration number** (at ClinicalTrials.gov or equivalent), where applicable.	Not Applicable	

Laboratory protocol	Information included in the manuscript?	In which section is the information available? (Reagents and Tools Table, Materials and Methods, Figures, Data Availability Section)
Provide DOI OR other citation details if **external detailed step-by-step protocols** are available.	Yes	Materials and Methods and References

Experimental study design and statistics	Information included in the manuscript?	In which section is the information available? (Reagents and Tools Table, Materials and Methods, Figures, Data Availability Section)
Include a statement about **sample size** estimate even if no statistical methods were used.	Yes	Materials and Methods
Were any steps taken to minimize the effects of subjective bias when allocating animals/samples to treatment (e.g. **randomization procedure**)? If yes, have they been described?	Yes	Materials and Methods
Include a statement about **blinding** even if no blinding was done.	Yes	Materials and Methods
Describe **inclusion/exclusion criteria** if samples or animals were excluded from the analysis. Were the criteria pre-established?<br><br>If sample or data points were omitted from analysis, report if this was due to attrition or intentional exclusion and provide justification.	Yes	Materials and Methods
For every figure, are **statistical tests** justified as appropriate? Do the data meet the assumptions of the tests (e.g., normal distribution)? Describe any methods used to assess it. Is there an estimate of variation within each group of data? Is the variance similar between the groups that are being statistically compared?	Yes	Materials and Methods and Figure legend

Sample definition and in-laboratory replication	Information included in the manuscript?	In which section is the information available? (Reagents and Tools Table, Materials and Methods, Figures, Data Availability Section)
In the figure legends: state number of times the experiment was **replicated** in laboratory.	Yes	Figure legend
In the figure legends: define whether data describe **technical or biological replicates**.	Yes	Figure legend

Ethics

Ethics	Information included in the manuscript?	In which section is the information available? (Reagents and Tools Table, Materials and Methods, Figures, Data Availability Section)
Studies involving **human participants**: State details of **authority granting ethics approval** (IRB or equivalent committee(s), provide reference number for approval.	Yes	Materials and Methods
Studies involving **human participants**: Include a statement confirming that **informed consent** was obtained from all subjects and that the experiments conformed to the principles set out in the WMA Declaration of Helsinki and the Department of Health and Human Services Belmont Report.	Yes	Materials and Methods
Studies involving **human participants:** For publication of **patient photos**, include a statement confirming that consent to publish was obtained.	Not Applicable	
Studies involving experimental **animals**: State details of **authority granting ethics approva**l (IRB or equivalent committee(s), provide reference number for approval. Include a statement of compliance with ethical regulations.	Yes	Materials and Methods
Studies involving **specimen and field samples:** State if relevant **permits** obtained, provide details of authority approving study; if none were required, explain why.	Not Applicable	

Dual Use Research of Concern (DURC)	Information included in the manuscript?	In which section is the information available? (Reagents and Tools Table, Materials and Methods, Figures, Data Availability Section)
Could your study fall under dual use research restrictions? Please check biosecurity documents and list of **select agents and toxins** (CDC): https://www.selectagents.gov/sat/list.htm	Not Applicable	
If you used a select agent, is the security level of the lab appropriate and reported in the manuscript?	Not Applicable	
If a study is subject to dual use research of concern regulations, is the name of the **authority granting approval and reference number** for the regulatory approval provided in the manuscript?	Not Applicable	

Reporting

The MDAR framework recommends adoption of discipline-specific guidelines, established and endorsed through community initiatives. Journals have their own policy about requiring specific guidelines and recommendations to complement MDAR.

Adherence to community standards	Information included in the manuscript?	In which section is the information available? (Reagents and Tools Table, Materials and Methods, Figures, Data Availability Section)
State if relevant guidelines or checklists (e.g., **ICMJE, MIBBI, ARRIVE, PRISMA**) have been followed or provided.	Not Applicable	
For **tumor marker prognostic studies,** we recommend that you follow the **REMARK** reporting guidelines (see link list at top right). See author guidelines, under 'Reporting Guidelines'. Please confirm you have followed these guidelines.	Not Applicable	
For **phase II and III randomized controlled trials**, please refer to the **CONSORT** flow diagram (see link list at top right) and submit the CONSORT checklist (see link list at top right) with your submission. See author guidelines, under 'Reporting Guidelines'. Please confirm you have submitted this list.	Not Applicable	

Data Availability

Data availability	Information included in the manuscript?	In which section is the information available? (Reagents and Tools Table, Materials and Methods, Figures, Data Availability Section)
Have **primary datasets** been deposited according to the journal's guidelines (see 'Data Deposition' section) and the respective accession numbers provided in the Data Availability Section?	Not Applicable	
Were **human clinical and genomic datasets** deposited in a public access-controlled repository in accordance to ethical obligations to the patients and to the applicable consent agreement?	Not Applicable	
Are **computational models** that are central and integral to a study available without restrictions in a machine-readable form? Were the relevant accession numbers or links provided?	Not Applicable	
If publicly available data were reused, provide the respective **data citations in the reference list**.	Not Applicable	
