# Supplementary material for: Platelet‐derived lipids promote insulin secretion of pancreatic β cells
Source: EMBO Mol Med. 2023 Jul 25;15(9):e16858. doi: 10.15252/emmm.202216858 (PMC10493578; doi:10.15252/emmm.202216858)
Supplement: Supplementary file 13 — Source Data for Figure Expanded View and Appendix [file EMMM-15-e16858-s001.zip › EV Figures.zip!/Figure EV4/EV4 F.pdf]

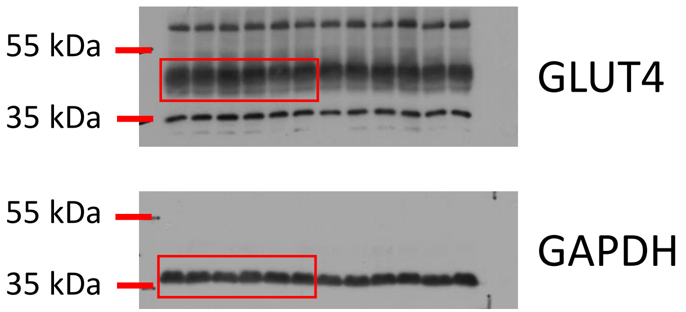
55 kDa
35 kDa
GLUT4
55 kDa
35 kDa
GAPDH

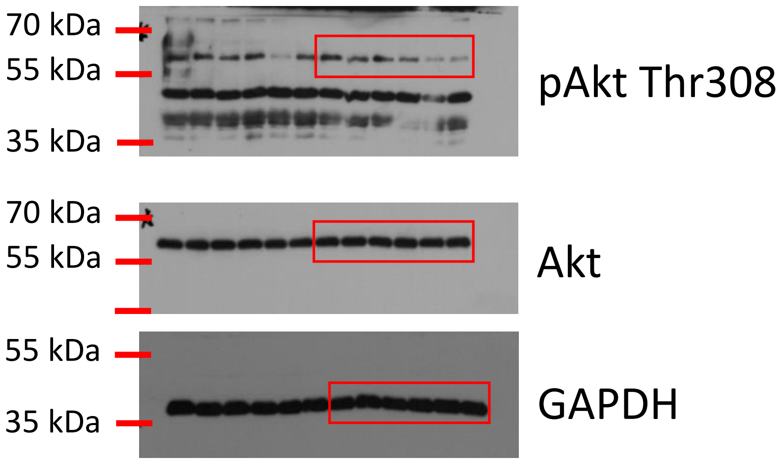
70 kDa
55 kDa
35 kDa
pAkt Thr308
70 kDa
55 kDa
Akt
55 kDa
35 kDa
GAPDH
